# Ranking network mechanisms by how they fit diverse experiments and deciding on *E. coli*'s ammonium transport and assimilation network

**DOI:** 10.1038/s41540-019-0091-6

**Published:** 2019-04-12

**Authors:** Kazuhiro Maeda, Hans V. Westerhoff, Hiroyuki Kurata, Fred C. Boogerd

**Affiliations:** 1 Frontier Research Academy for Young Researchers, Kyushu Institute of Technology, Kitakyushu, Fukuoka, Japan; 2 Department of Bioscience and Bioinformatics, Kyushu Institute of Technology, Iizuka, Fukuoka Japan; 3 Department of Molecular Cell Biology, Faculty of Science, VU University Amsterdam, O|2 building, Amsterdam, Netherlands; 4 Manchester Centre for Integrative Systems Biology, Manchester Interdisciplinary Biocentre, School of Chemical Engineering and Analytical Science, The University of Manchester, Manchester, UK; 5 Synthetic Systems Biology and Nuclear Organization, Swammerdam Institute for Life Sciences, University of Amsterdam, Amsterdam, Netherlands; 6 Biomedical Informatics R&D Center, Kyushu Institute of Technology, Iizuka, Fukuoka Japan

**Keywords:** Computer modelling, Differential equations, Numerical simulations

## Abstract

The complex ammonium transport and assimilation network of *E. coli* involves the ammonium transporter AmtB, the regulatory proteins GlnK and GlnB, and the central N-assimilating enzymes together with their highly complex interactions. The engineering and modelling of such a complex network seem impossible because functioning depends critically on a gamut of data known at patchy accuracy. We developed a way out of this predicament, which employs: (i) a constrained optimization-based technology for the simultaneous fitting of models to heterogeneous experimental data sets gathered through diverse experimental set-ups, (ii) a ‘rubber band method’ to deal with different degrees of uncertainty, both in experimentally determined or estimated parameter values and in measured transient or steady-state variables (training data sets), (iii) integration of human expertise to decide on accuracies of both parameters and variables, (iv) massive computation employing a fast algorithm and a supercomputer, (v) an objective way of quantifying the plausibility of models, which makes it possible to decide which model is the best and how much better that model is than the others. We applied the new technology to the ammonium transport and assimilation network, integrating recent and older data of various accuracies, from different expert laboratories. The kinetic model objectively ranked best, has *E. coli'*s AmtB as an active transporter of ammonia to be assimilated with GlnK minimizing the futile cycling that is an inevitable consequence of intracellular ammonium accumulation. It is 130 times better than a model with facilitated passive transport of ammonia.

## Introduction

Ammonium is the preferred nitrogen source for *E. coli*,^1^ which has two ammonium-assimilating routes: the glutamate dehydrogenase (GDH) pathway and the glutamine synthetase (GS)/glutamate synthase (GOGAT) cycle. The affinity of GS for ammonium (~0.1 mM) exceeds the affinity of GDH for ammonium (~1 mM).^2,3^ GS is intensively regulated via covalent modification and gene expression. Glutamate and glutamine are precursors to most cellular nitrogen.^4^ Notwithstanding its complexity, the regulation of ammonium assimilation is understood,^5^ except for an abyss in the understanding of the energetics, mechanisms and regulation of the transport.

*E. coli* is capable of growing in media with ammonium present in the low μM range because of the transporter AmtB, a member of the Amt/MEP/Rh transporter superfamily.^5^ The energetics of the transport remains a matter of debate (reviewed in refs. ^5–10^). Based on indirect structural information, AmtB was claimed to conduct uncharged NH_3_ through a channel.^11,12^ Accordingly, none of the cell’s free energy should be needed for nitrogen import across the cytoplasmic membrane. Boogerd et al.^8^ argued however that AmtB-mediated NH_3_ transport must be driven by some free energy input in order to accumulate NH_4_^+^ sufficiently for the growth observed at low extracellular ammonium concentrations; AmtB transporting NH_4_^+^ rather than NH_3_ would do the job. Computational modelling efforts have been devoted to revealing the complicated regulations in the ammonium assimilation network function as a whole^13–19^ (see also Section 11.2 of Supplementary Information). Although the existing models captured qualitative or semi-quantitative behaviours known to exist at the time, they have not been challenged with more recent quantitative experimental data, such as by Yuan et al.^19^

A quantitative model including transport, taking all relevant data sets into account simultaneously, consistent with fundamental thermodynamic and kinetic limitations and then at sufficient accuracy, is necessary for a decision on the ammonium transport controversy. Such integral models are still impossible however, now because of heterogeneity of the data sets in terms of quality, relevance, and completeness. Where the concentrations of most RNAs and proteins can be measured quantitatively, only a limited number of kinetic parameters have been measured experimentally, and then at rather diverse accuracies. Unmeasured parameters are to be estimated such that the model reproduces experimental observations accurately, but not too accurately as those observations are themselves subject to limited accuracy. The highly important expertise of biological domain experts should but cannot be taken into account neither robustly nor objectively. In order to identify parameter values uniquely by fitting the model to experimental data,^20–23^ much experimental data is required, particularly of the types that matter most, such as concentration time series. In some cases the most pertinent experimental data cannot be obtained because the experimental methodologies are unavailable or impossible. Given these limitations, how can modellers develop models that are sufficiently realistic to test hypotheses about the more complex underlying biology and to then engage in engineering?

The multiple and diverse experimental data sets, the kinetic and thermodynamic considerations of both transport and subsequent assimilation of ammonium, the knowledge about the complex regulatory network around GS, and the expert knowledge on parameter values, all come with uncertainties. This suggested to us that rather than to come to a binary decision as to which of the two models of ammonium transport and its regulation is right, we should develop a methodology that ranks the models in terms of their relative likelihood given all data and knowledge uncertainties. We used our five-pronged technology to achieve this: we quantitatively rank the two competing models of *E. coli's* ammonium transport and assimilation network in which direct experimental assays are impossible due to the high permeability of membranes to ammonia (NH_3_). The model that is 130 times more likely than its runner-up has the AmtB-mediated ammonium transport consumes cellular free energy and the regulator protein GlnK minimizes the futile cycling inevitably associated with the active transport of NH_3_.

## Results

### Parameter estimation and model plausibility

For kinetic models to be considered convincing, they should be capable of fitting experimentally measured variables. If the models require unrealistic parameter values for a good fit, they fail to comply with reality. Each individual model parameter comes with a certain level of uncertainty however. Accordingly, we divided model parameters into three classes (I–III) and a special class.

Class I parameters are considered most trustworthy, since their values have been directly experimentally determined (informed guesses). Class II parameters are somewhat less reliable, since they were not directly measured and they are therefore to be estimated to the best of our (current) biochemical or physiological knowledge relevant to the parameters at stake (educated guesses). In contrast, there are neither experimental data nor particular knowledge available for Class III parameters, and these are given reference values based on common sense and general knowledge (rough guesses). Finally, we use parameters that have reference values that are not allowed to be changed during the parameter estimation (special class), i.e. these are unsearched (US) parameters; their values are taken to be constant for obvious reasons or because there is firm evidence for their invariableness (constants).

The acceptable deviation of model parameter values from the corresponding reference values differ between individual parameters. Obviously, the last-mentioned special class harbours model parameters that are not allowed to change whatsoever, their reference value does not alter during the entire modelling exercise (no rubber bands). Next, we argue that class III parameters should be allowed to change freely from their reference values which holds the implication for modelling that there is no penalty for changing these values (infinitely flexible rubber bands). However, there are good reasons to trust the class I and II reference parameter values and as a consequence, altering their values should come with a certain penalty. We therefore used penalty weights for model parameter deviations that differed between class I and II parameters, i.e. ‘rubber bands’ of differing strengths were used; the penalty for a class I was heavier than for a class II parameter (For details, see Methods).

Now, the latter two classes of parameters enable us to quantify the overall model plausibility (MP). The procedure is a constrained optimization problem with different strengths of ‘rubber-bands’ applying to class I–III model parameters. Here the objective function (*f*) to be minimized is the weighted deviation of model parameter values from their reference values (informed, educated, and rough guesses) [Eq. (3a)], subject to constraint functions (*g*_1_, *g*_2_, …) [Eq. (3b)] and to lower and upper bounds on model parameter values [Eq. (3c)]. The constraint functions are squared residuals between experimental values and simulated values with certain allowable errors. The *g*_*i*_ values of >0 indicate the fitting is not sufficient. Therefore, we consider only models that exhibit *g*_*i*_ values of ≤0 for all constraints, without exception. Under this condition, the model will fit the experimentally observed behaviours. For such models, the objective function *f* will have certain values, which are nearly always >0. With this knowledge, we are able to develop a method to quantify MP based on the deviation of model parameters from reference class I and II parameter values. In short, we assume that a class I or II parameter follows the normal distribution in which the mean represents the reference value. The more the model needs to change parameters from their reference values, the less plausible the model is. We formulated *f* as the natural logarithm of the inverse of MP. Therefore, minimization of *f* is equal to maximization of MP (see Methods).

### Model construction

**Fig. 1:**
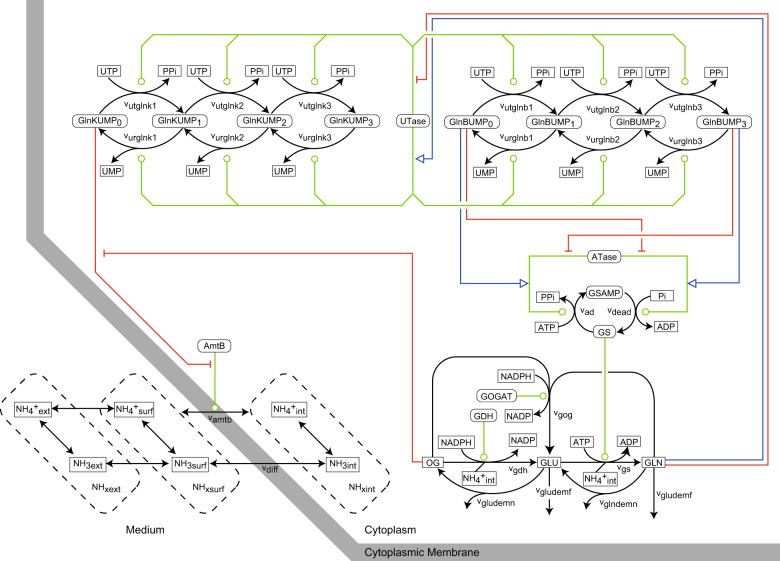
Schematic diagram of the *E. coli* ammonium transport and assimilation network. For simplicity, CADLIVE notation^14,24,25^ was used. Blue lines: activation, red lines: inhibition, green lines: catalytic action. The thick grey line indicates cytoplasmic membrane. The following abbreviations were used. GS: glutamine synthetase, GDH: glutamate dehydrogenase, GOGAT: glutamate synthase, ATase: adenylyltransferase/adenylyl-removing enzyme, UTase: uridylyltransferase/uridylyl-removing enzyme, GlnB and GlnK: nitrogen regulatory proteins, AmtB: ammonium transporter, OG: 2-oxoglutarate, GLU: glutamate, GLN: glutamine

The *E. coli* ammonium transport and assimilation network is shown in Fig. 1, using CADLIVE notation.^14,24,25^ The mathematical model is described in Tables S1–S4. We developed two models based either on the active or on the passive transporter hypothesis. Both models include the unmediated diffusion of NH_3_ and the AmtB-mediated ammonium transport (either active or passive) through the cytoplasmic membrane, and the regulation of AmtB by GlnK. For both the active and the passive transporter models, we assume that the driving force of the transport is the electro-chemical potential of NH_4_^+^ or NH_3_. The only difference between the active and passive transporter models is the theoretical accumulation factor of NH_4_^+^ (i.e. the ratio of the intracellular to the extracellular NH_4_^+^ concentration at the transporter equilibrium) denoted as *φ*.

**Fig. 2:**
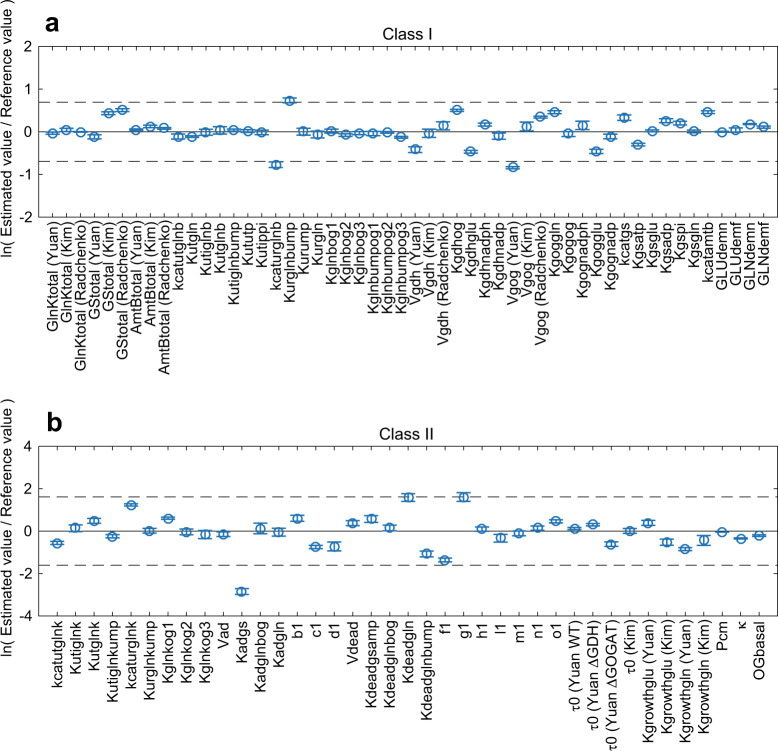
Deviation of estimated parameter values from their reference values for the refined active transporter model. **a** Class I parameters. **b** Class II parameters. We repeated parameter estimation five times. Circles represent mean values (*n* = 5). Error bars represent ± standard deviation. Dashed lines show the boundaries of a twofold change in (**a**) and a fivefold change in (**b**) above and below the reference values. The parameter values used for the figures are shown in Table S10

For the active transporter model, we assume AmtB is an active transporter of NH_3_ by which ammonium is transported as NH_4_^+^ or NH_3_ + H^+^ (See our Fig. S1a and Fig. 2CD of van Heeswijk et al.^5^). Because of the positive charge, NH_4_^+^ can accumulate inside cells up to a maximum concentration ratio *φ* determined by the membrane potential (inside negative). In the active transporter model, *φ* is for that reason a function of the membrane potential (Δ*ψ*):

1 $$\varphi = \exp \left( {\frac{{ - F \cdot \Delta \psi }}{{RT}}} \right),$$

where Δ*ψ* is the transmembrane electrical potential, *F* is the Faraday constant, *R* is the gas constant, *T* is the absolute temperature. Given Δ*ψ* = −150 mV, *φ* = 275 (or 313) at *T* = 310 K (or 303 K).

For the passive transporter model, we assume that AmtB is a facilitating passive transporter of NH_3_ (See our Fig. S1c and Fig. 2B of van Heeswijk et al.^5^), and thus only the concentration gradient of NH_3_ is the driving force of transport, and NH_3_ cannot accumulate inside cells. However, at equilibrium, NH_4_^+^ can then still be accumulated in or expelled from cells if the internal pH is lower or higher, respectively, than the external pH. Accordingly, in the passive transporter model, *φ* is a function of pH difference:

2 $$\varphi = 10^{\mathrm{pH}_{\mathrm{ext}} - \mathrm{pH}_{\mathrm{int}}},$$

where pH_ext_ and pH_int_ are extracellular and intracellular pH, respectively. Given pH_int_ = 7.6, *φ* = 0.25 (or 0.63) at pH_ext_ = 7.0 (or 7.4).

To solve the constrained optimization problem, we employed the real-coded genetic algorithm (GA) named IS-SR-REX^star^/JGG (see Section 4.3 of Supplementary Information). We performed the parameter estimation on the supercomputer Shirokane3. A single run for the parameter estimation took 12 h using 21 cores of Intel Xeon E5-2670 v3. Using a single core of a standard PC, such a single run would have taken some 10 days. Throughout this article, we performed 85 runs. Therefore, a supercomputer is essential to construct and test realistic kinetic models within a reasonable time scale.

The experimental training dataset on which the constraint functions are based is summarized in Table S9. For fair model comparison, the same constraints (*g*_1–52_) were used for the active and passive transporter models. We used experimental data from the following three papers. Yuan et al.^19^ (Yuan hereafter) grew *E. coli* (wild type, ΔGDH, and ΔGOGAT) on filters on top of a solid agarose-medium mixture to enable rapid, noninvasive sampling of the intracellular metabolome. To induce N-limitation in cells growing on the filter, the initial NH_4_^+^ concentration was set to 2 mM. Some 3 h later, the surface NH_4_^+^ concentration at the agarose-filter interface became measurably depleted. Since the underlying agarose provides a reservoir of ammonium, growth did not stop, but the growth rate was reduced, indicating that cultures were N-limited. Transferring the N-limited filter culture to plates with 10 mM NH_4_^+^ induced an N-upshift and partially restored the growth rate. At various time points for up to 30 min after the N-upshift, extracts from the cells on the filters were analysed by a set of LC-MS/MS methods. Kim et al.^26^ (Kim hereafter) developed microfluidic growth chambers in which NH_4_^+^ can be maintained continuously at low concentrations. From the growth rates, they estimated the intracellular NH_4_^+^ concentrations, the rates of the ammonium transport via AmtB, non-facilitated ammonia diffusion, and ammonium assimilation. Radchenko et al.^27^ (Radchenko hereafter) grew *E. coli* under N-limitation and then added 200 µM NH_4_^+^ to the liquid culture medium to cause a moderate N-upshift. The uridylylation state of GlnK and the binding of GlnK to AmtB were investigated prior to N-upshift and then periodically after the N-upshift.

### The active transporter model is 130 times more likely than the passive transporter model

We performed five independent runs of parameter estimation each for the active and the passive transporter models. GA found parameter sets that satisfied all the constraints (*γ* = 0) for both the active transporter model and the passive transporter model, indicating that both models can fit the training data used. However, there was a significant difference in the objective function value *f* (*p* = 0.008, Wilcoxon rank-sum test): 8.4 and 13.3 for the active and the passive transporter models, respectively. Since we defined *f* as the natural logarithm of the inverse of MP, we can calculate MP from *f* values: MP is 2.2 × 10^−4^ and 1.7 × 10^−6^ for the active and the passive transporter models, respectively. Therefore, MP of the active transporter model is 130 times higher than that of the passive transporter model. The difference in MP stems mainly from the difference in GS-related parameters. Since NH_4_^+^ cannot be accumulated in the passive transporter model, an unreasonably high V_max_ of GS is required to explain rapid cell growth at μM range of external NH_4_^+^ (see Section 9 of Supplementary Information).

### Refining the active transporter model

Since the active transporter model is much more likely than the passive transporter model, we hereafter focus on the active transporter model. First, we refined the active transporter model by incorporating Kim’s semi-experimental data which were calculated based on the active transporter hypothesis. Namely, we performed five new runs of parameter estimation with the full set of the constraint functions (*g*_1–58_). We plotted the deviation of the average of estimated values from their reference values. Class I and II parameters are shown in Fig. 2. Changes in 94% of class I parameters and 97% in class II parameters (circles in Fig. 2a, b) were less than twofold and fivefold on either side of the reference value, respectively, indicating that the model is able to reproduce the observed behaviours while using realistic parameter values.

Out of five independent runs of the GA, the parameter set that yielded the smallest value of the objective function (*f*) will be discussed further, also because the results to be shown for this particular set, essentially did not differ from those of the other 4 parameter sets (see Table S10 for all the estimated parameter sets).

**Fig. 3:**
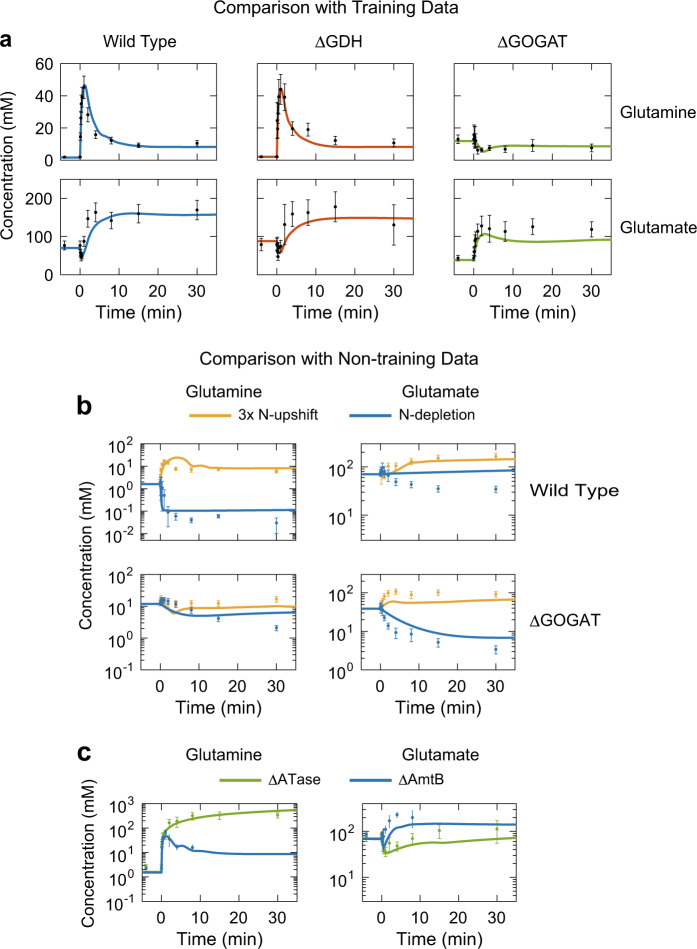
Comparison of model simulations with experimental data obtained by Yuan et al.^19^ **a** 13× N-upshift experiment, **b** various N-perturbations in the wild type and ΔGOGAT, **c** 13× N-upshift for ΔATase and ΔAmtB. Symbols and error bars represent experimental measurements as done by Yuan et al.^19^ Lines represent the values simulated by the refined active transporter model. At time zero, N-limited cells were transferred to plates with various NH_4_^+^ concentrations. The experimental data were obtained from Fig. 4 (13× N-upshift), Fig. 7 (various N-perturbations in the wild type and ΔGOGAT, and ΔATase), and Supplementary Fig. 5 (ΔAmtB) of Yuan et al.^19^

**Fig. 4:**
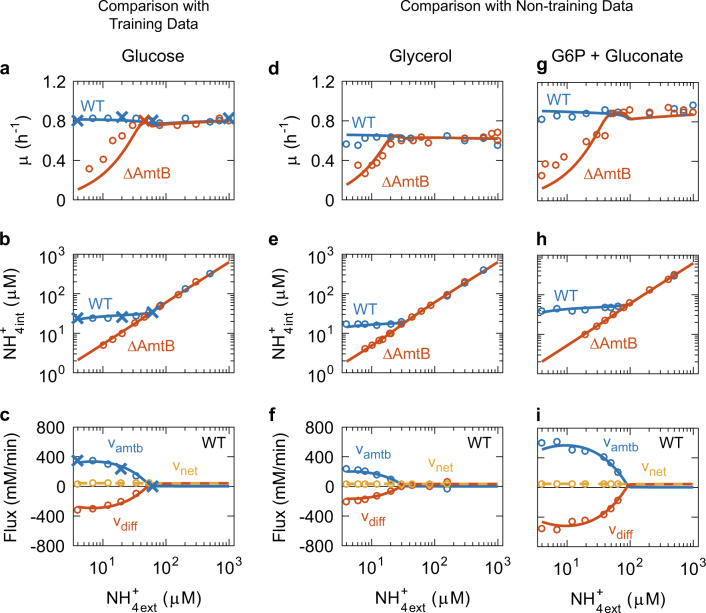
Comparison of model simulations with experimental data obtained by Kim et al.^26^ Steady-state changes of various variables as a function of extracellular ammonium. The carbon source is glucose for (**a**–**c**), glycerol for (**d**–**f**), and glucose-6P + gluconate for (**g**–**j**). Red, yellow, and blue circles represent experimental data. Cross symbols are data points actually used for parameter estimation. Solid lines with a corresponding colour represent the values simulated by the refined active transporter model. The experimental data in (**a**), (**d**), and (**g**) were obtained from Fig. 3A of Kim et al.^26^ The experimental data in (**b**), (**e**), and (**h**) were obtained from Fig. 3C of Kim et al.^26^ The experimental data in (**c**), (**f**) and (**i**) were kindly provided by Dr. Minsu Kim

**Fig. 5:**
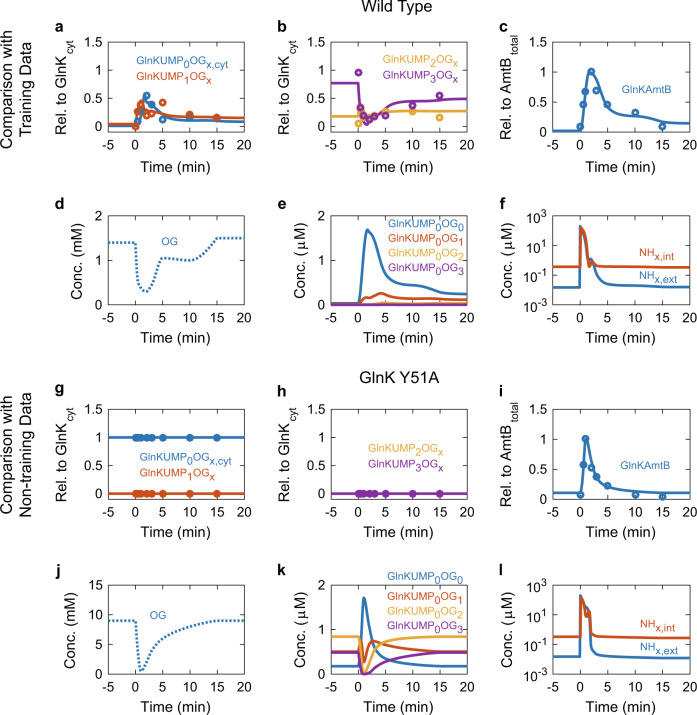
Comparison of model simulations with experimental data obtained by Radchenko et al.^27^ **a**–**f** The wild type and **g**–**l** GlnK Y51A mutant. Red, blue, yellow, and purple circles represent experimental data. Solid lines with a corresponding colour represent the values simulated by the refined active transporter model. Dotted lines represent assumed dynamic model inputs. “Rel. to GlnK_cyt_“ and “Rel. to AmtB_total_” indicate abundance relative to the total cytoplasmic GlnK and that to the total AmtB, respectively. GlnKUMP_0_OG_*x*,cyt_ indicates the sum of cytoplasmic GlnKUMP_0_OG_0_, GlnKUMP_0_OG_1_, GlnKUMP_0_OG_2_, and GlnKUMP_0_OG_3_. The same applies to GlnKUMP_1_OG_*x*_, GlnKUMP_2_OG_*x*_, and GlnKUMP_3_OG_*x*_ In (**h**), the yellow and purple lines are overlapping. At time zero, extracellular NH_x_ (NH_4_^+^ + NH_3_) was shifted from 15 nM to 200 μM. We obtained the experimental data in (**a**–**c**) and (**g**–**i**) by semi-quantifying blackness of the protein bands visible in Fig. 3AB of Radchenko et al.^27^ with ImageJ.^57^ We used Fig. 2A of Radchenko et al.^28^ to obtain the 2-oxoglutarate profile for the wild type. Since 2-oxoglutarate for GlnK Y51A mutant has not been measured, we optimized the dynamic 2-oxoglutarate model input

Next, we checked whether the refined active transporter model (with the smallest *f* value) actually fitted to training experimental data (see Comparison with Training Data in Figs 3–5). As shown in Fig. 3a, the refined active transporter model fits the experimental data reported by Yuan, with respect to the transient glutamine and glutamate changes after the 10 mM N-upshift.

The refined active transporter model successfully reproduced Kim’s experimental data for *E. coli* cells growing with glucose (Fig. 4a–c): The simulated growth rate of the wild type remained constant at ~0.8 h^−1^ regardless of the extracellular NH_4_^+^ concentration, and fits well to the experimental data (blue line in Fig. 4a). And, the growth of the ΔAmtB strain decreased at external NH_4_^+^ concentrations below ~40 μM (red line in Fig. 4a). Based on measured growth rates, Kim estimated the internal NH_4_^+^ concentration and rates of AmtB-mediated ammonium transport, unfacilitated diffusion, and net ammonium assimilation. The model fitted these quasi-experimental data as well: Lowering the extracellular NH_4_^+^ concentration from 1000 to 60 µM resulted in a linear decrease of the intracellular NH_4_^+^ concentration of the wild type from 628 down to 35 μM. At a further decrease of the external NH_4_^+^ concentration down to 4 µM, the internal NH_4_^+^ concentration remained virtually constant (blue line in Fig. 4b). The net influx of ammonium (v_net_) was constant at ~40 mM/min regardless of the extracellular NH_4_^+^ concentration (yellow line in Fig. 4c), a remarkable feat vis-à-vis the requirements and homeostasis of the cell. In all this, the model was consistent with the experimental data. However, the model also shows how all this works: Above 60 μM external NH_4_^+^, almost all the ammonium transport proceeds via unfacilitated NH_3_ diffusion (v_diff_). As the external NH_4_^+^ decreases further, the unfacilitated NH_3_ diffusion decreases to negative values (red line in Fig. 4c); this negative value of v_diff_ indicates NH_3_ back diffusion, i.e. passive outward NH_3_ permeation. The flux via AmtB (v_amtb_) increases just as much as the cells need (blue line in Fig. 4c), thereby minimizing the back diffusion.^26^

The refined active transporter model reproduced Radchenko’s experimental data for the wild type (Fig. 5a–c). The transient increase in both un-uridylylated GlnK and GlnK with one UMP-group, upon the N-upshift (200 µM NH_4_^+^) were both accurately reproduced by the model (Fig. 5a). Also, the relative steadiness of GlnK with two UMP-groups as well as the transient decrease in GlnK with three UMP-groups followed by the partial recovery were simulated by the model (Fig. 5b), as were the transient full inactivation of AmtB by the formation of the GlnKAmtB complex within 2 min after the N-upshift and the slower activation of AmtB by releasing GlnK (Fig. 5c).

### Model validation

In order to validate the refined active transporter model, we investigated whether this model fitted to experimental data that was not used for parameter estimation (see Comparison with Non-training Data in Figs 3–5). The model correctly reproduced both transient responses after the small N-upshift for the wild type and ΔGOGAT for Yuan’s experiments (yellow lines in Fig. 3b). The model’s behaviour upon the N-depletion was reasonable in a qualitative sense, except for the glutamate transient in the wild type (blue lines in Fig. 3b). Furthermore, the model reproduced the transient responses of ΔATase (green lines in Fig. 3c). Finally, the model also successfully fitted the glutamine and glutamate transients in ΔAmtB (blue lines in Fig. 3c; see also Section 10 of Supplementary Information).

Next, we investigated whether the model could reproduce non-training data for Kim’s experiments. We simulated differences in carbon sources by changing the value of the minimal doubling time *τ*_0_ (For details, see Section 2.2 of Supplementary Information). The model provided a good fitting to the experimental data for glycerol (Fig. 4d–f) and glucose 6 phosphate + gluconate (Fig. 4g–i) as growth substrates. This is valid for the specific growth rate of wild type and ΔAmtB (Fig. 4d, g), for the internal NH_4_^+^ concentration of both wild type and ΔAmtB (Fig. 4e, h), and for v_amtb_, v_diff_, and v_net_ of the wild type (Fig. 4f, i).

Finally, we investigated whether the model could reproduce non-training data for Radchenko’s experiments: The experimental data for the GlnK Y51A mutant that contains a variant GlnK protein that cannot be uridylylated. Only unuridylylated GlnK was present before and after the N-upshift (Fig. 5g, h), but, more importantly, the model reproduced the transient GlnKAmtB complex formation that was experimentally observed (Fig. 5i). Radchenko concluded that association and dissociation of the GlnKAmtB complex were independent of the uridylylation state of GlnK and that binding of 2-oxoglutarate (and ATP) to GlnK influenced the dynamics of its interaction with AmtB. Since 2-oxoglutarate has not been measured for this mutant, we optimized the time evolution of the 2-oxoglutarate concentration and found a similar pattern as in the wild type, but at higher concentrations (Fig. 5j). When using this dynamic 2-oxoglutarate input, the model predicted not only the transient increase/decrease pattern of GlnK free of bound 2-oxoglutarate, but also the opposite transient decrease/increase pattern of GlnK species with one, two or three bound 2-oxoglutarate molecules (Fig. 5k).

Radchenko did not measure the extracellular and intracellular ammonium concentrations either.^27,28^ Fig. 5f (wild type) and Fig. 5l (GlnK Y51A) show the external and internal NH_*x*_ (NH_4_^+^ + NH_3_) concentration as calculated by the refined active transporter model. Both NH_*x*_ traces look quite similar for the wild type and the mutant. Because of the rapid AmtB-mediated ammonium transport immediately after the N-upshift, the extracellular NH_*x*_ decreases to a sub-μM level within 2 min.

### GlnK is an indispensable regulator to limit ammonium/ammonia futile cycling

**Fig. 6:**
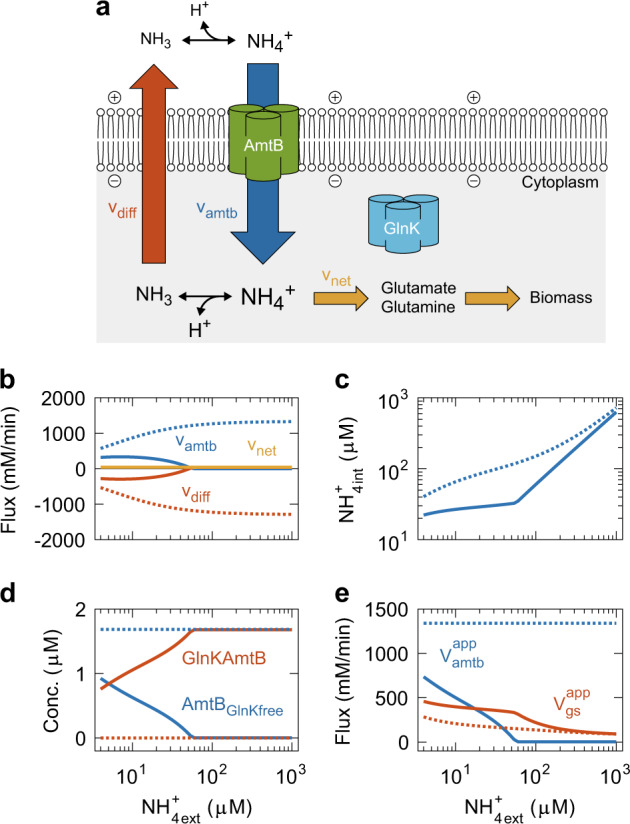
Investigation of the physiological role of GlnK. **a** Ammonium/ammonia futile cycling. When the extracellular NH_4_^+^ is low, AmtB expression is high, and GlnK dissociates from AmtB. AmtB transports NH_4_^+^ to increase the intracellular NH_4_^+^ concentration, supporting cell growth. The intracellular NH_4_^+^ is at equilibrium with its de-protonated form NH_3_ and H^+^. Since the intracellular NH_3_ concentration thereby becomes higher than the extracellular one, NH_3_ diffuses out of the cell, which is called “back diffusion”. The diffused NH_3_ is re-protonated. NH_4_^+^ transport, NH_4_^+^ de-protonation, NH_3_ back diffusion, and NH_3_ re-protonation together constitute a futile cycle as it dissipates the proton motive force. **b**–**e** In silico experiments showing an essential role of GlnK in limiting the futile cycling. The simulation was done for cells growing with glucose as in the experiments by Kim et al.^26^ The lines show the metabolic steady states of *E. coli* cells established shortly after the extracellular NH_4_^+^ was shifted from 4 μM to the concentration indicated on the horizontal axes. Solid and dotted lines show the computational results for wild type and virtual mutant, respectively. For the virtual mutant, all GlnK proteins were removed at the N-shift. In (**b**), yellow solid and dotted lines are overlapping

AmtB-mediated active ammonium transport and passive outward NH_3_ permeation together constitute a futile cycle^8,26^^,^^29,30^ (Fig. 6a). Therefore, Boogerd et al. hypothesized that GlnK is necessary not only to block but also to fine-tune the AmtB-mediated active NH_3_ transport in order to limit futile cycling whilst satisfying the demand of N input for growth.^8^ This hypothesis was addressed by the microfluidics experiments carried out by Kim^26^ and the latter experiments were used as training and non-training datasets in this paper.

To test this hypothesis further, we performed an in silico experiment that would be practically impossible to realize in vitro or in vivo. We subjected virtual wild type and mutant cells, adapted to 4 μM extracellular ammonium, to a sudden increase in the extracellular ammonium concentration (which was maintained afterwards). For the virtual mutant, we removed all GlnK proteins upon the N-upshift. In this analysis, we assumed that the membrane potential, ATP, NADPH, and cellular enzyme make-up remained constant after the N-upshift for at least 20 min. The interesting variables at steady state are presented in Fig. 6. After the N-upshift up to 60 μM extracellular NH_4_^+^, in the wild-type cells, GlnK adjusted the AmtB-mediated ammonium transport to the minimum flux necessary to maintain ~20 μM intracellular NH_4_^+^ given the back diffusion rate (solid lines in Fig. 6b–e). After the N-upshift beyond 60 μM extracellular NH_4_^+^, the wild-type cell completely blocked the AmtB-mediated transport. For the wild type, the three fluxes v_amtb_, v_diff_, and v_net_ (Fig. 6b**)** and the internal ammonium concentration (Fig. 6c) were almost indistinguishable from those shown in Fig. 4b, c, respectively, due to the fine-tuning of active ammonium transport by GlnK. The virtual mutant cannot limit the futile cycling because of the absence of GlnK (dotted lines in Fig. 6b–e). Its back diffusion rate of NH_3_ (red dotted line in Fig. 6b) was as high as 1300 mM/min at 1 mM extracellular ammonium. Assuming that NH_3_ is symported with one H^+^ and the H^+^/ATP coupling ratio is 3,^31–33^ the resultant dissipation of proton motive force would be equivalent to the loss of more than 400 mM/min of ATP. Considering that the overall ATP production rate is typically some 500 mM/min for cells growing exponentially with glucose in minimal medium,^34^ the energy loss by the futile cycling in this virtual mutant would amount to some 80% of the overall ATP production in the cell and not only preclude growth but even compromise maintenance of the living state. In summary, the most balanced model of ammonium assimilation produced here proves that GlnK is an indispensable regulator to hold in check the dissipation of proton motive force by the ammonium/ammonia futile cycling.

## Discussion

The new modelling technology presented here succeeded to integrate experimental data gathered in state-of-the-art experiments carried out by three independent research groups from two continents. Generally, it is difficult to develop a kinetic model capable of quantitatively reproducing different experimental data from different research groups. The data tend to address highly different aspects of the model at different accuracies, yet address the very same model so that parameter changes necessitated in one experimental setting destroy model correspondence with the data produced in another setting. With our new technology we succeeded to accommodate the experimental data obtained by the three research groups in terms of one and the same parameter set and one and the same model, except for the limited number of experiment-specific necessary adjustments: the refined active transporter model has 115 parameters in total, and only 11 parameters such as external pH and maximum specific growth rate needed experiment-specific adjustments.

Our endeavour was successful because we formulated our parameter estimation problem as a constrained optimization problem, allowed for different accuracies, and performed GAs on a supercomputer. Since there were multiple training sets of experimental data, it would have been impossible to tune parameters manually by trial and error: too many permutations. But what was possible and important, was the input of human expertise in judging the accuracy of parameters and variables used for model building. This was accomplished by allowing parameters to deviate from their reference value, as if allowing rubber bands to be stretched, but with ‘forces’ counteracting the deviations and with force constants that were adjusted by human experts so as to reflect the accuracy of the parameter. Also, variables were allowed to deviate from measured values since these were inaccurate to some extent as well. Once training data had been converted to constraint functions and reference values of kinetic parameters had been implemented, our technology estimated the most plausible values of kinetic parameters with minimal changes in the most firmly established parameters. To our knowledge, this type of doubly constrained optimization is not commonly used in kinetic modelling (e.g.^35,36^). Yet, this constrained optimization was here demonstrated to be highly effective in generating realistic in silico models. We took the ammonium transport and assimilation network merely as an example because it is so complex and controversial that it requires an objective comparison of model likelihood, which we delivered as concretely as a factor of likelihood (i.e. 130). The parameter estimation technique presented in this paper should essentially be applicable to other complex systems as long as reference parameter values and training data concerning model variables are available. We would like it to be tested in many other systems including cell biology, biotechnology, and microbial ecology.

Biology is complex and the performance of its models depends critically on parameter values and variables that are known with limited accuracy. Our technology is able to weigh the various certainties and uncertainties and integrate human expertise with parameters and experimental data. This should then produce the best available model given experimental data that are limited by resources as much as by feasibility of experimental determinations of some parameters and variables. The question was whether the model resulting from our new technology would be powerful enough to be decisive in an important biological issue. We showed that it was: when we applied our technology to ammonium assimilation in *E. coli*, the model in which AmtB is an active transporter and GlnK minimizes ammonium/ammonia futile cycling was 130 times more probable than the existing alternative model of facilitated passive transport of NH_3_.

The topic of active versus passive transport of ammonium by AmtB has been vividly debated.^5–10^ Structural studies reported in 2004 that AmtB is an NH_3_ channel, i.e. a passive transporter.^11,12^ Other studies thereafter seemed to support this conclusion.^37–42^ Although this may still be the consensus view, recent studies suggested that AmtB is an active NH_3_ (or passive NH_4_^+^) transporter.^43–47^ We tackled this elusive problem in an unprecedented way: kinetic modelling comprising the transporter, signal proteins, and metabolic enzymes. We developed two models based on the active and passive transporter hypotheses, respectively. Rather than coming to the more classical type of conclusion that one model is right and the other model is wrong, we discuss this in terms of relative likelihoods of alternative mechanisms. According to MP, the active transporter model is 130 times more likely than the passive transporter model.

The parameter estimation and model selection problem has been tackled before, notably by Bayesian approaches (see Liepe et al.^48^ and within), which also use prior knowledge about parameter values. While optimization algorithms (such as the one that we used) try to obtain a *single* parameter set that best enables a model to fit experimental data, Bayesian approaches try to find the *probability distribution* of such parameter sets. Bayesian approaches thus make it possible to assess confidence by assessing the probability distributions of unknown parameters. However, due to their much higher computational cost, Bayesian approaches have rarely been applied to models with more than a dozen unknown parameters (The largest model we have found to which a Bayesian approach has been applied has 19 unknown parameters. See Liepe et al.^48^ and references therein). Our constrained optimization-based approach is a computationally much cheaper alternative to Bayesian approaches: We were able to integrate prior information into parameter estimation and still obtained parameter estimates for 94 unknown (class I–III) parameters within a reasonable computational time (12 h).

Our constrained optimization-based approach can deal with the uncertainty not only in a single but also in multiple network connections. To illustrate this, we consider the hypothetical situation in which we investigate whether GlnB is (de)uridylylated (it is common knowledge that this is the case). We have created an alternative model in which GlnB is not (de)uridylylated, i.e. we eliminated 6 edges for *v*_*utglnb*1-3_ and *v*_*urglnb*1-3_ in Fig. 1. Apart from the GlnB (de)uridylylation, the rest of the network of the alternative model was the same as the refined active transporter model. Next, we performed parameter estimation for the alternative model and obtained an MP of 3.6 × 10^−8^, which is much smaller than that of the refined active transporter model (1.1 × 10^−4^). We concluded that the alternative model is less plausible, and that it is highly likely that GlnB is (de)uridylylated. We conclude that as long as models can fit training experimental data and reference parameter values are provided, our approach can rank competing models with different network connections.

We performed five independent runs of parameter estimation each for the active, the passive, and the refined active transporter models. In Table S10, we showed all the estimated parameter sets. These parameter sets provide comparable fitting results as all the constraints are satisfied (*g*_*i*_ ≤ 0 for all *i*). The parameter sets in Table S10 have similar parameter values for each model, indicating that parameters are almost identified: The median of the coefficient of variation (CV) for all search parameters is 0.05, and all the CVs are less than 0.38. The key to identifiability is to penalize deviations of parameters from the reference values. In principle, the parameters of class I and II (i.e. penalized parameters) can be uniquely determined. Other parameter sets which are very different from those in Table S10 may fit training data (*g*_*i*_ ≤ 0 for all *i*); however, it is likely that they provide smaller MP than that in Table S10. For more discussion of this important issue, see Section 13 of Supplementary Information.

Our methodology cannot rank different parameter sets with the same deviations of parameters from the reference values. To rank such parameter sets, other model ranking criteria such as robustness to parameter changes can be used.^49,50^ We conducted a sensitivity analysis for important model variables and fluxes (Table S13). In the passive transporter model, the growth rate is highly sensitive to internal and external pH changes because only the concentration gradient of NH_3_ is the driving force of the ammonium transport. In contrast, the growth rate is less sensitive in the active transporter model. Therefore, not only in terms of the parameter deviation from the reference values but also in terms of robustness, the active transporter model is better than the passive transporter model.

Where in chemistry and physics new technologies are first tested in silico, this has been much less successful in bioengineering. The thousands of nonlinearly interacting processes in biology have long been the legitimate culprit: insufficient data were available. Thanks to functional genomics and biochemical technologies the quantity of experimental data should no longer constitute a limitation. Indeed, we are almost able to measure every single of the thousands of molecule types that run living cells. It would seem that the quantity of data should suffice for a ‘deep biology’ understanding and for an engineering of cell-based systems by using dynamic in silico replica models of the intracellular networks. Such integral kinetic modelling should enable prediction of complex dynamic responses to complex perturbations,^51,52^ including those of precision bioengineering.

We have here developed a new, balanced modelling technology that enables decisions on the relative rather than absolute validity of mechanisms in crucial biological networks. Indeed, an innovation is that with this analysis we refrain from concluding that the one model is right and the other wrong. We consider it likely that this relative likelihood of the two models will change with more experimental data becoming available in the future. We see the future of biotechnology as one in which models are not true or false but more and less likely at rates driven by the developing amounts of big data. Hence we see the methodology we here developed as big (data) biotechnology.

## Methods

### Constrained optimization-based parameter estimation

The parameter estimation problem can be formulated as a constrained optimization problem:

3a $${\mathrm{minimize}}\ f({\mathbf{p}}),$$

3b $${\mathrm{subject}}\,{\mathrm{to}}\ {\mathbf{g}}({\mathbf{p}}) \le {\mathbf{0}},$$

3c $${\mathbf{p}}^L \le {\mathbf{p}} \le {\mathbf{p}}^U,$$

where **p** = (*p*_1_, *p*_2_, …) is the search parameter vector, i.e. a set of parameters to be searched, and *p*_*i*_ is the *i*th parameter. *f* is the objective function that evaluates deviation of parameter estimates from the reference values (*reference* refers to the values used to initiate the search, for which measured values, educated guesses or rough guesses are taken). *f* is defined as the natural logarithm of the inverse of MP (see Section 4.1 of Supplementary Information). **g** = (*g*_1_, *g*_2_, …) is the constraint function vector that evaluates model fitting to training data, i.e. the experimental data to which an in silico model should fit. If the fitting is not sufficient, *g*_*i*_ takes a positive value. For example, a constraint function that evaluates model fitting to time course data is given by

4 $$g_i({\mathbf{p}}) = \frac{1}{n}\mathop {\sum}\limits_{j = 1}^n {\left( {\frac{{x_j^{\mathrm{sim}} - x_j^{\mathrm{exp}}}}{{x_j^{\mathrm{exp}}}}} \right)^2} - \varepsilon _i^2,$$

where *x*_*j*_^sim^ and *x*_*j*_^exp^ are simulated and experimental data points of a model variable, *n* is the number of data points, and *ε*_*i*_ is the allowable error. For the actual equations, see Section 4.2 of Supplementary Information. In Eq. (3), **p**^*L*^ and **p**^*U*^ are the lower bound and upper bound vectors, respectively. The aim of the constrained optimization problem is to minimize parameter deviation from the reference values (*f*) while keeping a good fit to training experimental data (**g** ≤ **0**). The modelling workflow is illustrated in Fig. S5.

We divided search parameters into three classes (see Table S4): Class I, II, and III parameters are those for which measured values (I), educated guesses (II), and rough guesses (III) are available. In this study, the objective function *f* is given by:

5 $$f({\mathbf{p}}) = \lambda _I\mathop {\sum}\limits_{p_i \in {\mathrm{Class}}\,I} {\left( {\ln \frac{{p_i}}{{p_i^ \ast }}} \right)^2} + \lambda _{II}\mathop {\sum}\limits_{p_i \in {\mathrm{Class}}\,II} {\left( {\ln \frac{{p_i}}{{p_i^ \ast }}} \right)^2} + \lambda _{III}\mathop {\sum}\limits_{p_i \in {\mathrm{Class}}\,III} {\left( {\ln \frac{{p_i}}{{p_i^ \ast }}} \right)^2} ,$$

where *p*_i_^*^ is the reference value of the *i*th parameter, and *λ*_*j*_ (*j* = *I*, *II*, *III*) is the class-related penalty weight for a parameter change (*λ*_*I*_ > *λ*_*II*_ > *λ*_*III*_ ≥ 0). We used *λ*_*I*_ = 1.0407, *λ*_*II*_ = 0.1930, and *λ*_*III*_ = 0. We derived Eq. (5) based on MP. Thus, we can calculate MP from *f*:

6 $$MP({\mathbf{p}}) = e^{ - f({\mathbf{p}})}.$$

Therefore, minimizing *f* is equal to maximizing MP. For more details on the objective function *f* and MP, see Sections 3 and 4 of Supplementary Information.

In the constrained optimization, the constraint violation *γ* is used to check if the constraint equations [Eq. (3b)] are satisfied.

7 $$\gamma ({\mathbf{p}}) = \mathop {\sum}\limits_{i = 1}^{n_{\mathrm{constraint}}} {\left[ {\max \left( {0,g_i({\mathbf{p}})} \right)} \right]^2} ,$$

where the max function returns the higher value of two inputs: 0 and *g*_*i*_(**p**). *γ* = 0 indicates that all the constraint functions are satisfied, i.e. the model fits the training data. *γ* > 0 indicates one or more constraint functions are not satisfied. Different allowable errors *ε*_*k*_ (*k* = *I*, *II*, *III*) are used for the constraint functions in a manner similar to the penalty weights *λ*_*j*_ (*j* = *I*, *II*, *III*) for the objective function (see Section 4 of Supplementary Information). The aim of the constrained optimization can be rephrased as to find parameter vectors that provide the smallest possible *f* value while satisfying *γ* = 0.

Equation (3) is the standard formalism of constrained optimization problems, and a wide variety of optimization algorithms and software (e.g.^53–55^) have been proposed to deal with them. We employ a genetic algorithm (GA) named IS-SR-REX^star^/JGG (Iterative Start and Stochastic Ranking, Real-coded Ensemble Crossover star/Just Generation Gap). GAs are metaheuristic techniques that have been developed inspired by the evolution of living organisms. IS-SR-REX^star^/JGG has been a slightly modified from SR-REX^star^/JGG.^53^ For details, see Section 4.3 of Supplementary Information. Here we provide a brief description of how IS-SR-REX^star^/JGG solves the constrained optimization problem [Eq. (3)]:

1. Randomly generate an initial population in which each individual is characterized by a set of different values for search parameters. To evaluate *g*_*i*_ and *γ* for each individual, Yuan, Kim, and Radchenko experiments are simulated.
2. Select a subset of individuals from the population. The selected ones are called parents.
3. Generate children using the parents (outside the population) and compute *f* and *γ* for them.
4. Select some children that provide small values of *f* and *γ*.
5. Replace the parents in the population with the selected children, thereby creating a partly changed population, while maintaining the number of individuals.
6. If the *f* and *γ* have not been decreased for many generations, go to the step (1). Otherwise, go the step (2). The iteration is stopped at the predefined computational time (12 h).

By performing the steps (1)-(6), parameter sets providing large *f* and *γ* values are eliminated from the population, and those providing small *f* and *γ* values emerge. Eventually, we can obtain an individual (i.e. a parameter set) that provides a small *f* value with *γ* = 0. The stochastic ranking enables GAs to reduce both *f* and *γ* values in a balanced way.^56^ Fig. S6 illustrates how the GA works in a simple problem.

## Supplementary information

Supplementary Information

Tables S1 - S4 and S10 - S13

Simulation Program in MATLAB

## Data Availability

The authors declare that the data supporting the findings of this study are available within the paper and its supplementary information files.

## Supplementary information

**Supplementary information** accompanies the paper on the *npj Systems Biology and Applications* website (10.1038/s41540-019-0091-6).

## Code availability

Custom C and MATLAB codes used in this study are available upon request. The MATLAB codes for the refined active transporter model are provided as a supplementary file of this article (41540_2019_91_MOESM3_ESM.zip). The SBML file for the refined active transporter model for Kim’s experiment is available from BioModels database (MODEL1901090001).

## References

[1] Reitzer L (2003). Nitrogen assimilation and global regulation in Escherichia coli. Annu. Rev. Microbiol..

[2] Miller RE, Stadtman ER (1972). Glutamate synthase from Escherichia coli. An iron-sulfide flavoprotein. J. Biol. Chem..

[3] Sakamoto N, Kotre AM, Savageau MA (1975). Glutamate dehydrogenase from Escherichia coli: purification and properties. J. Bacteriol..

[4] Wohlhueter, R. M., Schutt, H. & Holzer, H. in *The Enzymes of Glutamine Metabolism* (eds S. Prusiner & E. R. Stadtman) 44–64 (Academic Press, New York, 1973).

[5] van Heeswijk WC, Westerhoff HV, Boogerd FC (2013). Nitrogen assimilation in Escherichia coli: putting molecular data into a systems perspective. Microbiol. Mol. Biol. Rev..

[6] Andrade SL, Einsle O (2007). The Amt/Mep/Rh family of ammonium transport proteins. Mol. Membr. Biol..

[7] Neuhauser B, Dynowski M, Ludewig U (2014). Switching substrate specificity of AMT/MEP/ Rh proteins. Channels.

[8] Boogerd FC (2011). AmtB-mediated NH3 transport in prokaryotes must be active and as a consequence regulation of transport by GlnK is mandatory to limit futile cycling of NH4(+)/NH3. FEBS Lett..

[9] Javelle A (2007). Structural and mechanistic aspects of Amt/Rh proteins. J. Struct. Biol..

[10] Winkler FK (2006). Amt/MEP/Rh proteins conduct ammonia. Pflugers Arch..

[11] Khademi S (2004). Mechanism of ammonia transport by Amt/MEP/Rh: structure of AmtB at 1.35 A. Science.

[12] Zheng L, Kostrewa D, Berneche S, Winkler FK, Li XD (2004). The mechanism of ammonia transport based on the crystal structure of AmtB of Escherichia coli. Proc. Natl Acad. Sci. USA.

[13] Bruggeman FJ, Boogerd FC, Westerhoff HV (2005). The multifarious short-term regulation of ammonium assimilation of Escherichia coli: dissection using an in silico replica. FEBS. J..

[14] Kurata H, Masaki K, Sumida Y, Iwasaki R (2005). CADLIVE dynamic simulator: direct link of biochemical networks to dynamic models. Genome Res..

[15] Ma H, Boogerd FC, Goryanin I (2009). Modelling nitrogen assimilation of Escherichia coli at low ammonium concentration. J. Biotechnol..

[16] Ma, H., Boogerd, F. C. & Goryanin, I. Corrigendum to “Modelling nitrogen assimilation of Escherichia coli at low ammonium concentration” [J. Biotechnol. 144 (2009) 175–183]. *J Biotechnol* **150**, 207 (2010). 10.1016/j.jbiotec.2009.09.003 19761805

[17] Masaki K, Maeda K, Kurata H (2012). Biological design principles of complex feedback modules in the E. coli ammonia assimilation system. Artif. Life.

[18] Gosztolai A (2017). GlnK facilitates the dynamic regulation of bacterial nitrogen assimilation. Biophys. J..

[19] Yuan J (2009). Metabolomics-driven quantitative analysis of ammonia assimilation in E. coli. Mol. Syst. Biol..

[20] Banga JR, Balsa-Canto E (2008). Parameter estimation and optimal experimental design. Essays Biochem..

[21] Jaqaman K, Danuser G (2006). Linking data to models: data regression. Nat. Rev. Mol. Cell Biol..

[22] Sontag ED (2003). For differential equations with r parameters, 2r+1 experiments are enough for identification. J. Nonlinear Sci..

[23] van Beek JH, Hauschild AC, Hettling H, Binsl TW (2009). Robust modelling, measurement and analysis of human and animal metabolic systems. Philos. Trans. A Math. Phys. Eng. Sci..

[24] Kurata H, Matoba N, Shimizu N (2003). CADLIVE for constructing a large-scale biochemical network based on a simulation-directed notation and its application to yeast cell cycle. Nucleic Acids Res..

[25] Kurata H (2007). Extended CADLIVE: a novel graphical notation for design of biochemical network maps and computational pathway analysis. Nucleic Acids Res..

[26] Kim M (2012). Need-based activation of ammonium uptake in Escherichia coli. Mol. Syst. Biol..

[27] Radchenko MV, Thornton J, Merrick M (2014). Association and dissociation of the GlnK-AmtB complex in response to cellular nitrogen status can occur in the absence of GlnK post-translational modification. Front. Microbiol..

[28] Radchenko MV, Thornton J, Merrick M (2010). Control of AmtB-GlnK complex formation by intracellular levels of ATP, ADP, and 2-oxoglutarate. J. Biol. Chem..

[29] Kleiner D (1981). The transport of NH3 and NH4+ across biological membranes. Biochim. Biophys. Acta.

[30] Neijssel OM, Buurman ET, Teixeira de Mattos MJ (1990). The role of futile cycles in the energetics of bacterial growth. Biochim. Biophys. Acta.

[31] Stouthamer AH, Bettenhaussen C (1973). Utilization of energy for growth and maintenance in continuous and batch cultures of microorganisms. A reevaluation of the method for the determination of ATP production by measuring molar growth yields. Biochim. Biophys. Acta.

[32] Boogerd FC, van Verseveld HW, Torenvliet D, Braster M, Stouthamer AH (1984). Reconsideration of the efficiency of energy transduction in Paracoccus denitrificans during growth under a variety of culture conditions. Arch. Microbiol..

[33] Tomashek JJ, Brusilow WS (2000). Stoichiometry of energy coupling by proton-translocating ATPases: a history of variability. J. Bioenerg. Biomembr..

[34] Gonzalez JE, Long CP, Antoniewicz MR (2017). Comprehensive analysis of glucose and xylose metabolism in Escherichia coli under aerobic and anaerobic conditions by 13C metabolic flux analysis. Metab. Eng..

[35] Tohsato Y, Ikuta K, Shionoya A, Mazaki Y, Ito M (2013). Parameter optimization and sensitivity analysis for large kinetic models using a real-coded genetic algorithm. Gene.

[36] Kotte O, Zaugg JB, Heinemann M (2010). Bacterial adaptation through distributed sensing of metabolic fluxes. Mol. Syst. Biol..

[37] Khademi S, Stroud RM (2006). The Amt/MEP/Rh family: structure of AmtB and the mechanism of ammonia gas conduction. Physiology.

[38] Javelle A, Thomas G, Marini AM, Kramer R, Merrick M (2005). In vivo functional characterization of the Escherichia coli ammonium channel AmtB: evidence for metabolic coupling of AmtB to glutamine synthetase. Biochem. J..

[39] Soupene E, He L, Yan D, Kustu S (1998). Ammonia acquisition in enteric bacteria: physiological role of the ammonium/methylammonium transport B (AmtB) protein. Proc. Natl Acad. Sci. USA.

[40] Soupene E, Lee H, Kustu S (2002). Ammonium/methylammonium transport (Amt) proteins facilitate diffusion of NH_3_ bidirectionally. Proc. Natl Acad. Sci. USA.

[41] Kustu S, Inwood W (2006). Biological gas channels for NH_3_ and CO_2_: evidence that Rh (Rhesus) proteins are CO_2_ channels. Transfus. Clin. Biol..

[42] Li XD, Lupo D, Zheng L, Winkler F (2006). Structural and functional insights into the AmtB/Mep/Rh protein family. Transfus. Clin. Biol..

[43] Hall JA, Yan D (2013). The molecular basis of K+ exclusion by the Escherichia coli ammonium channel AmtB. J. Biol. Chem..

[44] Fong RN, Kim KS, Yoshihara C, Inwood WB, Kustu S (2007). The W148L substitution in the Escherichia coli ammonium channel AmtB increases flux and indicates that the substrate is an ion. Proc. Natl Acad. Sci. USA.

[45] Lamoureux G, Javelle A, Baday S, Wang S, Berneche S (2010). Transport mechanisms in the ammonium transporter family. Transfus. Clin. Biol..

[46] Wang S, Orabi EA, Baday S, Berneche S, Lamoureux G (2012). Ammonium transporters achieve charge transfer by fragmenting their substrate. J. Am. Chem. Soc..

[47] Baday S, Wang S, Lamoureux G, Berneche S (2013). Different hydration patterns in the pores of AmtB and RhCG could determine their transport mechanisms. Biochemistry.

[48] Liepe J (2014). A framework for parameter estimation and model selection from experimental data in systems biology using approximate Bayesian computation. Nat. Protoc..

[49] Morohashi M (2002). Robustness as a measure of plausibility in models of biochemical networks. J. Theor. Biol..

[50] Bates DG, Cosentino C (2011). Validation and invalidation of systems biology models using robustness analysis. IET Syst. Biol..

[51] Tummler K, Klipp E (2018). The discrepancy between data for and expectations on metabolic models: How to match experiments and computational efforts to arrive at quantitative predictions?. Curr. Opin. Syst. Biol..

[52] Miskovic L, Tokic M, Fengos G, Hatzimanikatis V (2015). Rites of passage: requirements and standards for building kinetic models of metabolic phenotypes. Curr. Opin. Biotechnol..

[53] Maeda K, Boogerd FC, Kurata H (2018). libRCGA: a C library for real-coded genetic algorithms for rapid parameter estimation of kinetic models. IPSJ Trans. Bioinform..

[54] Ji X, Xu Y (2006). libSRES: a C library for stochastic ranking evolution strategy for parameter estimation. Bioinformatics.

[55] Balsa-Canto E, Henriques D, Gabor A, Banga JR (2016). AMIGO2, a toolbox for dynamic modeling, optimization and control in systems biology. Bioinformatics.

[56] Runarsson TP, Yao X (2000). Stochastic ranking for constrained evolutionary optimization. IEEE Trans. Evol. Comput..

[57] Schneider CA, Rasband WS, Eliceiri KW (2012). NIH Image to ImageJ: 25 years of image analysis. Nat. Methods.

