# Supplementary material for: Ranking network mechanisms by how they fit diverse experiments and deciding on E. coli's ammonium transport and assimilation network
Source: NPJ Syst Biol Appl. 2019 Apr 12;5:14. doi: 10.1038/s41540-019-0091-6 (PMC6461619; doi:10.1038/s41540-019-0091-6)
Supplement: Supplementary file 1 — Supplementary Information [file 41540_2019_91_MOESM1_ESM.pdf]

Ranking network mechanisms by how they fit diverse experiments and deciding on *E. coli*'s ammonium transport and assimilation network

Kazuhiro Maeda, Hans V. Westerhoff*, Hiroyuki Kurata*, Fred C. Boogerd
*Corresponding authors

Supplementary Information

Contents

Supplementary Figures 3
Figure S1 Reaction schemes of AmtB-mediated ammonium transport 3
Figure S2 Transition of the values of objective function (f) and constraint violation (γ) of the best individual 4
Figure S3 Simulation of NH_x diffusion and consumption in Yuan's experiments 5
Figure S4 Deviation of estimated parameter values from their reference values 6
Figure S5 Modeling workflow 7
Figure S6 Constrained optimization-based parameter estimation 8
Supplementary Tables 10
Tables S1 – S4 10
Table S5 Parameter modifications needed to simulate various mutants in Yuan's experiments 10
Table S6 Extracellular NH_x concentrations for Yuan's experiments 11
Table S7 Parameter modifications needed to simulate various experimental conditions in Kim's experiments 12
Table S8 Parameters for calculation of AmtB, GlnK, and GS concentrations 13
Table S9 Training data for the parameter estimation 14
Tables S10 – S13 15
Supplementary Notes 16
1 Model Description 16
1.1 Regulation of GS 16
1.2 Regulation of AmtB 16
1.3 AmtB-mediated ammonium transport 17
1.4 Regulation of AmtB 18
1.5 Ammonium/ammonia (NH_x) transfer 19
1.6 Details on GlnK-, GlnB-, AmtB- and GS-related variables 20

2 Simulation Settings 21
2.1 Yuan's experiments 22
2.2 Kim's experiments 23
2.3 Radchenko's experiments 25
3 Model Plausibility 26
4 Parameter Estimation 27
4.1 Objective function 27
4.2 Constraint functions 28
4.3 IS-SR-REX^{star}/JGG 35
5 Apparent Discrepancy between Kim's and Yuan's Experimental Data 35
6 Discussion on the Mass Transfer Capacity Coefficient (k_{db}) 38
6.1 The Yuan model implicitly includes a diffusion boundary for NH_x 38
6.2 Thickness of the diffusion barrier 39
7 Derivation of the Rate Equation of AmtB-mediated Ammonium Transport 41
7.1 Active transport of NH_3 41
7.2 Facilitated passive transport of NH_3 42
8 Derivation of Key Parameter Values 44
8.1 Number of nitrogen atoms per liter cell volume (N_0) 44
8.2 Cell volume (V_{cell}) and surface area (A_{cell}) 44
8.3 V_{max} of GS, GOGAT, and GDH (V_{gs}, V_{gog}, and V_{gdh}) 45
8.4 AmtB and GlnK-related parameters ($k_{catamtb}$, K_{amtbnh}, $AmtB_{total}$, $GlnK_{total}$, and $K_{glnkog1\text{-}3}$) 45
8.5 GLU_{demn}, GLU_{demf}, GLN_{demn}, and GLN_{demf} 46
9 Detailed Discussion on Comparison of Active and Passive Transporter Models 48
9.1 Passive transporter model requires at least 1600 mM/min for V_{max} of GS 48
9.2 Parameters that are different between the active and passive transporter models 48
9.3 Low value for model plausibility is to be expected 49
9.4 Active transporter model is more likely even if values of λ_i are changed 49
9.5 Active transporter model is more likely even if K_m of GS for NH_4^+ is allowed to be changed 50
10 Why Are the Metabolome Phenotypes Similar for ΔAmtB and Wild Type in Yuan's Experiments? 50
11 Model Improvements and Summary of Other Models 50
11.1 Model improvements 50
11.2 Summary of other models 51
12 Constrained Optimization-based Approach vs. Conventional Approach 52
13 Parameter Identifiability 54
14 Rationale for the Implementation of Model Plausibility and Objective Function 55
References 59

Supplementary Figures

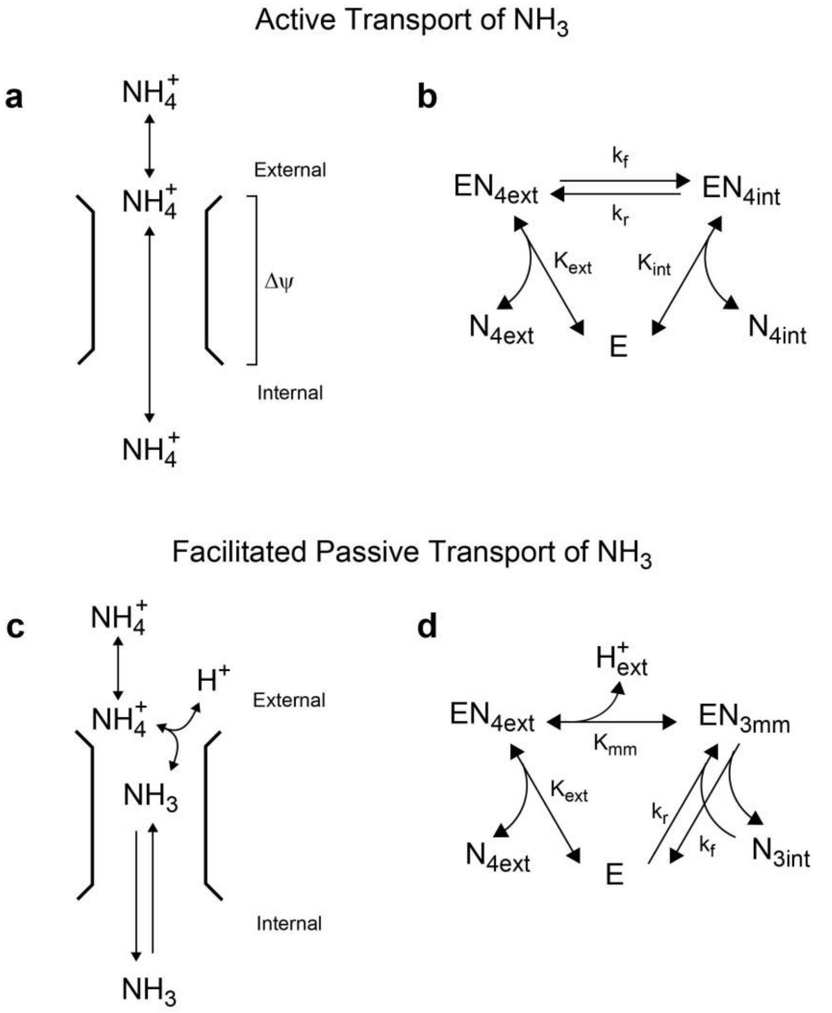

Figure S1 Reaction schemes of AmtB-mediated ammonium transport

(ab) Active transport of NH_3. (a) Scheme of active transport across the cytoplasmic membrane: first, external NH_4^+ binds to AmtB. It is assumed that the AmtB-NH_4^+ complex has two states: One open towards the outside and the other open towards the inside. After binding, NH_4^+ is released into the cytoplasm. Because of the transmembrane electric potential ($\Delta\psi$), it is difficult for the internal NH_4^+ to reach the binding site of AmtB. (b) Schematic diagram of the active transport: AmtB is distributed over three states, i.e. empty carrier (E), carriers with NH_4^+ bound at the binding site which is either open towards the outside (EN_{4ext}) or the inside (EN_{4int}). N_{4ext} and N_{4int} depict external and internal NH_4^+, respectively. K_{ext}, K_{int}, k_f, and k_r are parameters. (cd) Facilitated passive transport of NH_3. (c) Scheme of passive transport across the cytoplasmic membrane: first, external NH_4^+ binds to AmtB. Then, NH_4^+ is deprotonated, and the resultant NH_3 passes through the pore of AmtB. (d) Schematic diagram of the facilitated passive transport: AmtB is distributed over three states, i.e. empty carrier (E), carrier with NH_4^+ bound at the binding site (EN_{4ext}), and carrier with NH_3 at the middle membrane channel (EN_{3mm}). N_{4ext} and N_{3int} depict external NH_4^+ and internal NH_3, respectively. K_{ext}, K_{mm}, k_f, and k_r are parameters.

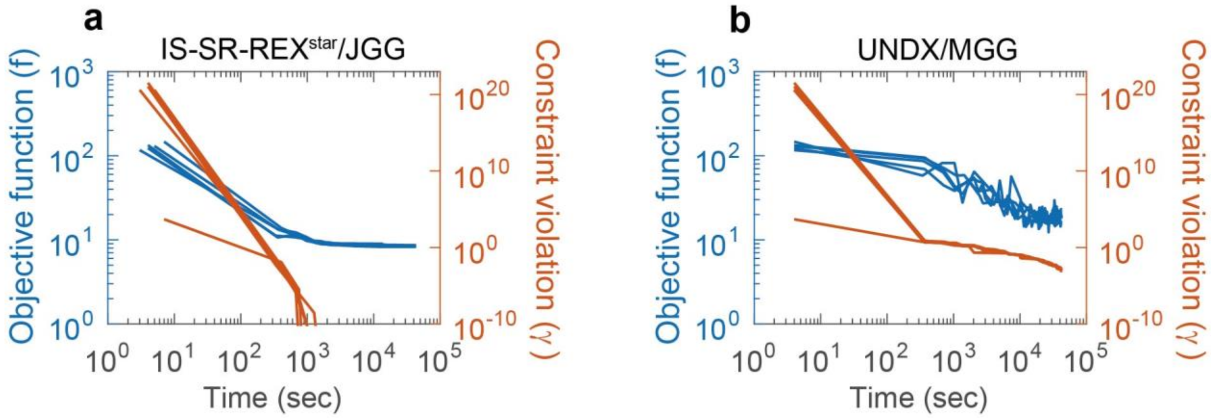

Figure S2 Transition of the values of objective function (*f*) and constraint violation (*γ*) of the best individual

(a) IS-SR-REX^{star}/JGG and (b) UNDX/MGG were employed to solve the parameter estimation problem for the active transporter model. The constraint functions $g_{1\text{-}52}$ were employed. The GAs were performed five times. For each run of the GAs, we used 21cores cores of Intel Xeon E5-2670 v3 on the supercomputer Shirokane3. In (a), the values of constraint violation reach zero around 1,000 sec. IS-SR-REX^{star}/JGG decreases the objective function and the constraint violation simultaneously, which is realized by stochastic ranking. UNDX/MGG firstly tries to decrease the constraint violation to zero. Therefore, the objective function sometimes increases in return for decreases in the constraint violation. IS-SR-REX^{star}/JGG found solutions (parameter sets with $\gamma = 0$) within 30 min. However, UNDX/MGG was not able to find solutions within 12 hours.

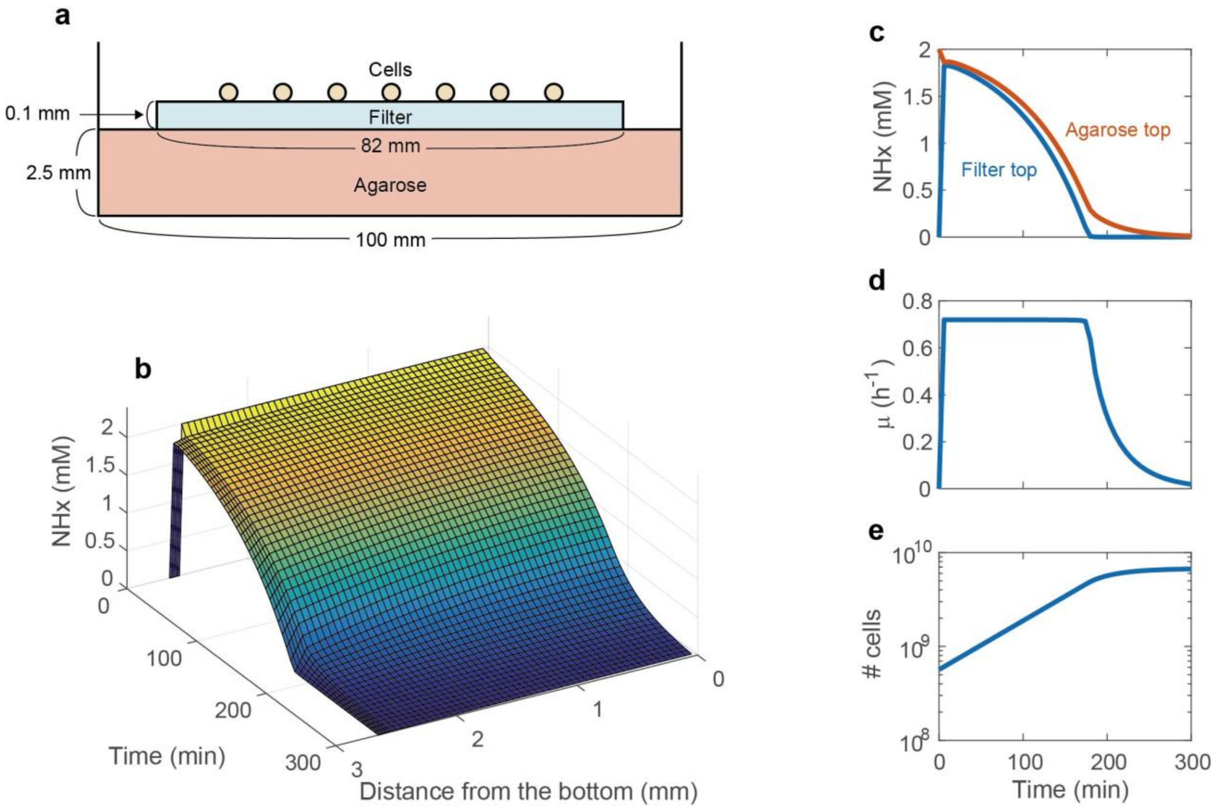

Figure S3 Simulation of NH_x diffusion and consumption in Yuan's experiments

We simulated NH_x (i.e. NH_4^+ + NH_3) diffusion and consumption in Yuan's experiments (triangles in Figure 2A of [1]). (a) Geometry of plates. The plate consists of agarose, filter, and *E. coli* cells. We assumed that Yuan used 20 ml of agarose per 10-cm plate [2-4], resulting in an agarose layer of 2.5 mm thick. We assumed the filter was 0.1 mm thick. (b) NH_x distribution in the plates. (c) NH_x concentration on top of the filter (blue) and that on top of the agarose (red). (d) The specific growth rate. (e) The number of cells on the filter. We modeled NH_x diffusion in agarose and filter based on Fick's law of diffusion. From the top of the filter, the cells consume NH_x and grow. We assumed homogenous distribution of the cells. We assumed that the diffusion coefficient for NH_x in agarose is equal to the diffusion coefficient in water: 1.12 x 10^{-7} m^2/min [5]. Based on the triangles of Figure 1b of [2], we estimated the diffusion coefficient for NH_x in filter: 2.23 x 10^{-8} m^2/min. The specific growth rate was modeled by a Monod equation $\mu = \mu_{max}$ $[NH_x]$ / (K + $[NH_x]$), where $[NH_x]$ is the NH_x concentration at the top of the filter. The maximum specific growth rate in Yuan's experiments is 0.72 h^{-1}. Since *E. coli* can grow at the maximum specific growth rate at 4 μM external NH_4^+ [6], K must be less than 4 μM. Thus, we used μ_{max} = 0.72 h^{-1} and K = 1 μM. A single cell consumes NH_x from the top of the filter at a rate of $v_{single} = \mu N_0 V_{cell}$, where N_0 = 3 mol-N/L-cyt and V_{cell} = 2.15 x 10^{-18} m^3 [7]. The total NH_x consumption rate is $v_{total} = v_{single}$ X, where X is the number of cells. The number of cells follows the ordinary differential equation: $dX/dt = \mu X$ with the initial condition $X(t) = X_0$. We assumed that Yuan used 5 ml of culture with ~0.085 OD_{650} to inoculate cells on the filter [1]. Assuming 1.11 x 10^9 cells/(ml OD_{600}) [8] and 1.2 OD_{600}/OD_{650}, the initial number of cells is 5.66 x 10^8. In our simulation (b-e), the specific growth rate was predicted to be 0.72 h^{-1} for 0 – 180 min and 0.16 h^{-1} for 180 – 300 min. These specific growth rates are close to Yuan's observation (0.72 h^{-1} and 0.19 h^{-1}, respectively , see triangles in Figure 2A of [1]), validating our simulation.

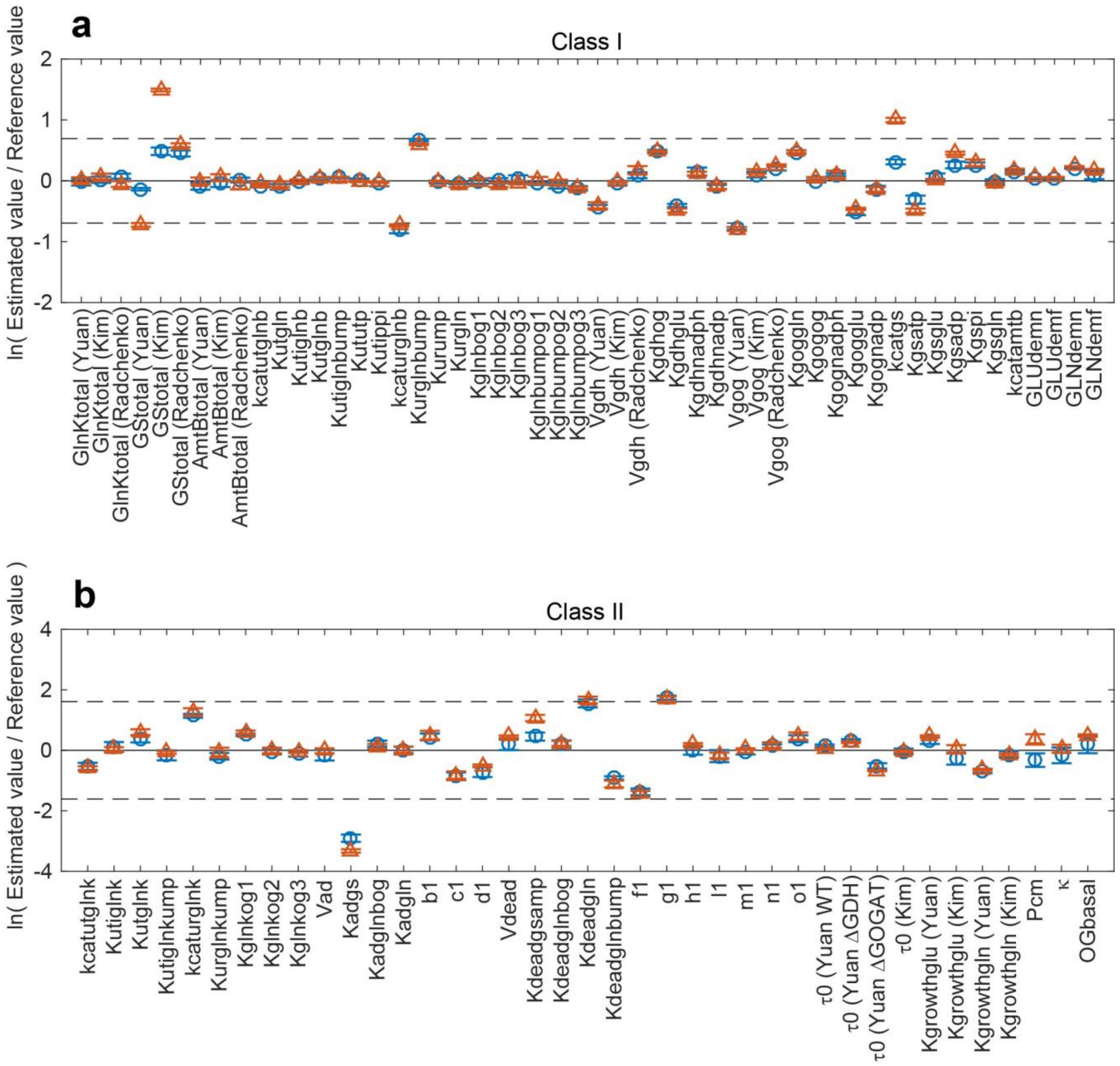

Figure S4 Deviation of estimated parameter values from their reference values

(a) Class I parameters. (b) Class II parameters. Blue circles and red triangles represent the active and the passive transporter models, respectively. We repeated parameter estimation five times. Circles and triangles represent mean values ($n = 5$). Error bars represent ± standard deviation. Dashed lines show the boundaries of a twofold change in (a) and a fivefold change in (b) above and below the reference values. It should be noted that in parameter estimation we assumed that $\ln(p_i/p_i^*)$ follows the normal distribution with the standard deviation of ln(2) for class I and ln(5) for class II. The parameter values used for the figures are shown in **Table S10**.

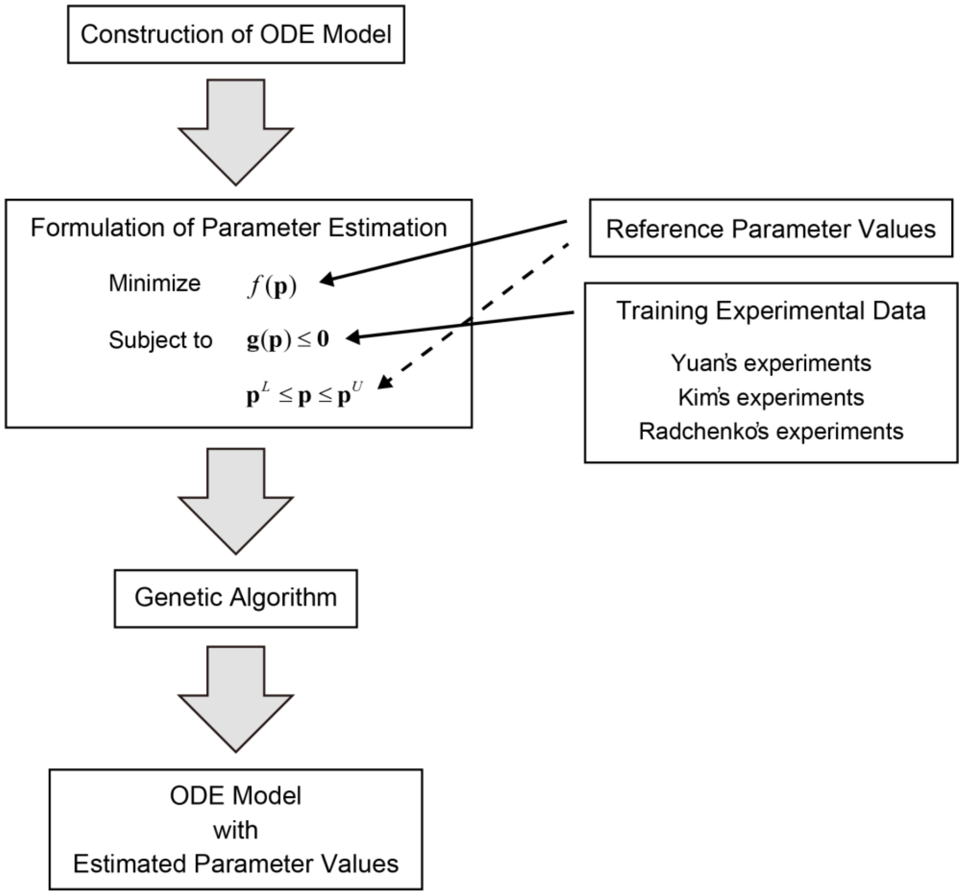

Figure S5 Modeling workflow

First, we develop the ordinary differential equation (ODE) model for the *E. coli* ammonium transport and assimilation network. Second, we formulate the parameter estimation problem as a constrained optimization problem. The objective function (*f*) quantifies the deviation of parameters from their reference values, and constraint functions (**g**) quantify the difference between model behavior and training experimental data. The lower bound ($\mathbf{p}^L$) and the upper bound ($\mathbf{p}^U$) of the parameter search space are determined based on the reference values. We obtain reference parameter values and training experimental data from literature. Next, we run the genetic algorithm (GA) to solve the constrained optimization problem. Finally, we obtain the ODE model with the estimated parameter values. The modeling workflow is the same for the active, the passive, and the refined active transporter models. We perform 5 independent GA runs for each model. Thus, we obtain 5 parameter sets for each model.

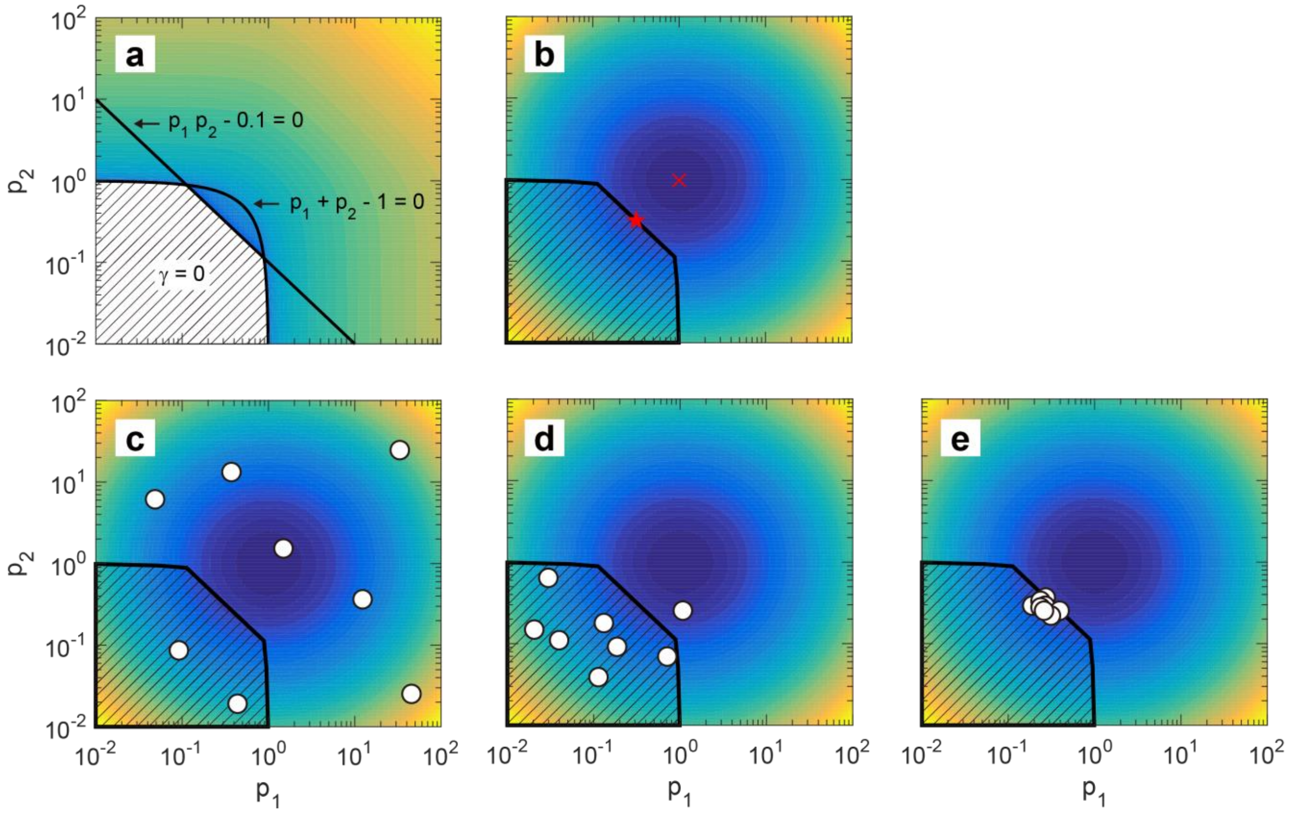

Figure S6 Constrained optimization-based parameter estimation

To illustrate how the constrained optimization-based parameter estimation works, we consider the following simple problem here:

$$\begin{aligned}
&\text{minimize} && f(p_1,p_2)=\lambda_1\left(\ln\frac{p_1}{p_1^*}\right)^2+\lambda_2\left(\ln\frac{p_2}{p_2^*}\right)^2 \\
&\text{subject to} && g_1(p_1,p_2)=p_1p_2-0.1\le 0 \\
& && g_2(p_1,p_2)=p_1+p_2-1\le 0 \\
& && 10^{-2}\le p_1\le 10^2 \\
& && 10^{-2}\le p_2\le 10^2
\end{aligned} \quad , \cdots\cdots\text{(Figure S6-1)}$$

where $p_1^* = 1$, $p_2^* = 1$, $\lambda_1, = 1$, $\lambda_2 = 1$. The objective function $f(p_1,p_2)$ is the two-variable version of Eq. (S4.1-1) and quantifies deviations of p_1 and p_2 from p_1^* and p_2^*. The simple constraints $g_1(p_1,p_2) \le 0$ and $g_2(p_1,p_2) \le 0$ enable us to illustrate the feasible region (the subspace in which all the constraints are satisfied). The aim is to find the pair of p_1 and p_2 which minimizes $f(p_1,p_2)$ and satisfies $g_1(p_1,p_2) \le 0$, $g_2(p_1,p_2) \le 0$, $10^{-2} < p_1 < 10^2$, and $10^{-2} < p_2 < 10^2$. The constraint violation $\gamma(p_1,p_2)$ is provided as $\gamma(p_1,p_2) = [\max(0,g_1(p_1,p_2))]^2 + [\max(0,g_2(p_1,p_2))]^2$. Only if $g_1 \le 0$ and $g_2 \le 0$, γ takes the minimum: $\gamma = 0$. In (a), the γ value is visualized by a change of color: it increases as the color changes from blue to yellow. The shaded area shows the feasible region in which the γ value takes the minimum: $\gamma = 0$. In the modeling context, the feasible region is a parameter space in which the model fits training experimental data (with certain allowable errors). In (b)-(e), the f value is visualized by a change of color: it increases as the color changes from blue to yellow. In (b), the x symbol shows the position at which f takes the minimum: $f = 0$. At the position indicated by the star symbol in (b), the

f value is minimized with $\gamma = 0$. Thus, the aim of Eqs. (Figure S6-1) is to find p_1 and p_2 at the star symbol. It should be noted that p_1 and p_2 cannot be uniquely estimated based only on the constraints: any combinations of p_1 and p_2 in the feasible region (the shaded area) can satisfy $\gamma = 0$. However, if the constraints are combined with the minimization of f, p_1 and p_2 can be uniquely estimated. In (c)-(e), the parameter estimation procedure by genetic algorithms (GAs) is illustrated. In (c)-(e), each white circle shows an "individual" (a pair of p_1 and p_2). The GA initiates the search by generating random individuals within the upper and lower bounds (c). Individuals which provide smaller f and γ values are considered better than those who provide larger f and γ values. The GA gives a slightly higher priority to reducing γ (which is done by stochastic ranking [9]). By iterating selection and reproduction operations, the GA converges individuals to the feasible region (d). In the feasible region, $\gamma = 0$ regardless of the values of p_1 and p_2. Thus, individuals in the feasible region try to reduce the f value in order to outperform others. Eventually, individuals converge to the minimum f value in the feasible region (e). In the modeling context, the GA generates random parameter sets (c), and then tries to find parameter sets which provide a good fitting, i.e. $\gamma = 0$ (d). Finally, it reduces the deviation of parameters from the reference values, i.e. it reduces the f value (e). Thus, penalized parameters can be identified by minimizing f with constraints satisfied. It should be noted that if the feasible regions are inappropriately shaped, parameters can be non-identifiable. For example, if the feasible region is annulus-shaped and surrounds the x symbol in (b), p_1 and p_2 will be non-identifiable. However, such a situation rarely happens in practice. When it happens, constraints need to be modified.

Supplementary Tables

Tables S1 – S4

Tables S1 – S4 are shown in a separate XLSX file.

Table S5 Parameter modifications needed to simulate various mutants in Yuan's experiments

The asterisks show the values for wild type (shown in **Table S4**).

Strain	Parameter modification
ΔGDH	$V_{gdh} = 0$
ΔGOGAT	$V_{gdh} = 3 \times V_{gdh}^*$, $V_{gog} = 0$, $[GS_{total}] = 0.5 \times [GS_{total}]^*$, $[GlnK_{total}] = 0$, $[AmtB_{total}] = 0$
ΔATase	$V_{ad} = 0$, $V_{dead} = 0$
ΔAmtB	$[AmtB_{total}] = 0$

Table S6 Extracellular NH_x concentrations for Yuan's experiments

In Yuan's experiments [1], *E. coli*-inoculated filters on N-limited media are transferred onto solid media with different NH_x concentrations, causing changes in the extracellular NH_x concentration.

N-change	Strain	Extracellular NH_x (mM)	
		Before N-change	After N-change
13x N-upshift	WT	0.75	10
	ΔGDH	0.75	10
	ΔGOGAT	1	10
	ΔATase	0.75	10
	ΔAmtB	0.75	10
3x N-upshift	WT	0.75	2
	ΔGOGAT	1	2
N-downshift	WT	0.75	0.05
	ΔGOGAT	1	0.05

Table S7 Parameter modifications needed to simulate various experimental conditions in Kim's experiments

The asterisks show the values for glucose (shown in **Table S4**). It should be noted that Kim deleted the entire *glnK-amtB* operon for ΔAmtB mutant [6].

Carbon source	Strain	Parameter modification
Glucose	Wild type	-
	ΔAmtB	$[AmtB_{total}] = 0$, $[GlnK_{total}] = 0$
Glycerol	Wild type	$\tau_0 = 1.29 \times \tau_0^*$
	ΔAmtB	$\tau_0 = 1.29 \times \tau_0^*$, $[AmtB_{total}] = 0$, $[GlnK_{total}] = 0$
G6P + Gluconate	Wild type	$\tau_0 = 0.89 \times \tau_0^*$
	ΔAmtB	$\tau_0 = 0.89 \times \tau_0^*$, $[AmtB_{total}] = 0$, $[GlnK_{total}] = 0$

Table S8 Parameters for calculation of AmtB, GlnK, and GS concentrations

For ΔAmtB, [$AmtB_{total}$] = 0 and [$GlnK_{total}$] = 0, and thus $P_{AmtB,std}$, H_{AmtB}, K_{AmtB}, and f_{AmtB} are not provided for.

Carbon source	Strain	$P_{AmtB,std}$ (-)	H_{AmtB} (-)	K_{AmtB} (mM)	f_{AmtB} (-)	$P_{GS,std}$ (-)	H_{GS} (-)	K_{GS} (mM)	f_{GS} (-)
Glucose	WT	6.3	1.9	0.058	28	23.5	0.50	0.42	2.6
	ΔAmtB	-	-	-	-	27.0	0.47	0.025	2.5
Glycerol	WT	1.3	2.8	0.032	103	13.8	1.15	0.062	6.1
	ΔAmtB	-	-	-	-	13.2	1.20	0.063	6.7
G6P + Gluconate	WT	11.6	1.6	0.079	23	28.9	0.37	0.0049	2.9
	ΔAmtB	-	-	-	-	27.1	0.35	0.00055	5.4

Table S9 Training data for the parameter estimation

In the parameter estimation, we used experimental data from [1, 2, 6, 10] as training data. The experimental data were transformed by using the correct pK_a for ammonium and a representative cell volume and cell surface area. The constraint functions shown in the table were constructed based on the training data. Auxiliary constraints ($g_{34\text{-}52}$) are not shown in the table. Please note that $g_{53\text{-}58}$ are used only for refining the active transporter model but not for the comparison of the active and the passive transporter models. For the mathematical expressions of the constraint functions, see Section 4.2.

Training data	Constraint function
Glutamate and glutamine concentrations of WT, ΔGDH, and ΔGOGAT in 13x N-upshift experiment (Figure 4 of [1])	$g_1 - g_6$
Specific growth rates of WT, ΔGDH, and ΔGOGAT in 13x N-upshift experiment (Figure 2A and Supplementary Figure 2 of [1])	$g_7 - g_{12}$
Ammonium assimilation fluxes of WT in 13x N-upshift experiment (Figure 2A of [1])	$g_{13} - g_{14}$
Ratios of the glutamate production flux via GOGAT to total glutamate production flux [2]	$g_{15} - g_{16}$
Specific growth rates for glucose as the C-source (Open and solid triangles of Figure 3A of [6])	$g_{17} - g_{21}$
N-assimilation rate for glucose as the C-source [6]	g_{22}
Uridylylation states of GlnKs of WT (Figure 3A of [10])	$g_{23} - g_{26}$
GlnK-AmtB complex of WT (Figure 3B of [10])	g_{27}
Ratios of glutamate and glutamine consumption rates [1]	$g_{28} - g_{33}$
Internal NH_4^+ concentrations for glucose as the C-source (Open and solid triangles of Figure 3C of [6])	$g_{53} - g_{55}$
AmtB-mediated ammonium transport rates for glucose as the C-source [6]	$g_{56} - g_{58}$

Tables S10 – S13

Tables S10 – S13 are shown in a separate XLSX file.

Supplementary Notes

1 Model Description

The schematic diagram of the *E. coli* ammonium transport and assimilation network is shown in **Figure 1**, where CADLIVE notation [11-13] is used for simplicity. The mathematical model is described in **Tables S1–S4**. The equations relevant to GDH, GS, GOGAT, GlnB, UTase, and ATase are based on the Bruggeman model [14]. The rate equations for glutamate and glutamine consumption, and the cell growth function are taken from the Yuan model [1]. The models in this paper newly include unmediated diffusion of NH_3, AmtB-mediated ammonium transport through the cytoplasmic membrane, and regulation of AmtB by GlnK. For Yuan's experiments, we modelled an NH_x diffusion barrier between the medium and the cell surface so as to explain an apparent contradiction between two recent experimental data [1, 6] (see Section 5; In this paper, we use the term NH_x when we do not wish to discriminate between NH_4^+ and NH_3**)**. In order to simulate multiple experiments conducted by different research groups, the values of some parameters have to be modified since parameters such as enzyme expression levels and external pH differ among experiments (see Section 2).

1.1 Regulation of GS

The activity of GS is feedback-controlled based on intracellular NH_4^+ availability: As the intracellular NH_4^+ increases, 2-oxoglutarate decreases and glutamine increases, leading to (partial) inactivation of GS. The enzyme level of GS is controlled by gene expression and GS activity by covalent modification. The models in this paper include only the covalent modification. The concentration of total GS is constant for Yuan's and Radchenko's experiments. In Kim's experiments, it is modified based on measured promoter activity.

The covalent modification of GS is controlled via ATase, UTase and GlnB. ATase is a bifunctional, ambiguous enzyme, which inactivates GS through a progressive adenylylation of its 12 subunits, and is also able to deadenylylate the adenylylated form of GS (GSAMP) [15, 16]. The adenylylation reaction is stimulated by the nitrogen signaling protein GlnB and glutamine [17, 18]. The deadenylylation reaction is stimulated by GlnBUMP and 2-oxoglutarate. The uridylylation and deuridylylation of GlnB is catalyzed by UTase, which is also an ambiguous enzyme [19]. Uridylylation and deuridylylation activities of UTase are respectively inhibited and stimulated by glutamine. Taken together, a decrease (increase) in nitrogen availability causes activation (inactivation) of GS.

1.2 Regulation of AmtB

The activity of AmtB is also feedback-controlled based on intracellular ammonium availability. The level of AmtB is controlled by gene expression, and AmtB activity is regulated via binding of the protein GlnK. The models in this paper include only the regulation via binding of GlnK. The concentration of total AmtB is taken to be constant for Yuan's and Radchenko's experiments. In Kim's experiments, it is modified based on measured promoter activity.

GlnK, a paralogue of GlnB, is the main regulator of AmtB-mediated ammonium transport. GlnK binds to

AmtB and thereby blocks ammonium transport [10, 20, 21]. At low NH_4^+ concentration, GlnK is uridylylated by UTase in the same manner as GlnB and then loses its ability to bind to AmtB. An increase in NH_4^+ concentration leads to deuridylylation of GlnK, enabling GlnK to block the ammonium transport. It has been reported that the complex formation between AmtB and GlnK is affected by metabolites, such as 2-oxoglutarate, ATP, and ADP. 2-oxoglutarate is an indicator of the cellular nitrogen status. Recently, Radchenko et al. revealed the function of ATP/ADP binding [22]: GlnK has an ATPase activity that is inhibited by 2-oxoglutarate. Hydrolysis of ATP to ADP enables GlnK to change its confirmation to bind to AmtB. Taken together, a decrease (increase) in nitrogen availability causes activation (inactivation) of the AmtB-mediated ammonium transport.

1.3 AmtB-mediated ammonium transport

In this paper, we developed two models based either on the active or on the passive transporter hypothesis. For both the active transporter and the passive transporter models, the rate equation for AmtB-mediated ammonium transport is described by:

$$v_{amtb} = \frac{k_{catamtb}[AmtB_{GlnKfree}]\left([NH_{4surf}^{+}] - \frac{[NH_{4int}^{+}]}{\varphi}\right)}{K_{amtbnh} + [NH_{4surf}^{+}]}, \cdots\cdots (S1.3\text{-}1)$$

where $[AmtB_{GlnKfree}]$ is the operative form of AmtB, $[NH_4^+{}_{surf}]$ and $[NH_4^+{}_{int}]$ are the NH_4^+ concentrations at the cell surface and in the cytoplasm, respectively. $k_{catamtb}$ and K_{amtbnh} are the turnover rate and the Michaelis constant, respectively. φ is the theoretical accumulation factor of NH_4^+, i.e. the ratio of the intracellular to the extracellular NH_4^+ concentration at the transporter equilibrium ($v_{amtb} = 0$). For the active transporter model, φ is based on membrane potential. For the passive transporter model, φ is determined by the pH difference between both sides of cytoplasmic membrane. For fair comparison, we use the same reference values of $k_{catamtb}$ and K_{amtbnh} for both models. For derivation of Eq. (S1.3-1), see Section 7.

For the active transporter model, we assume AmtB is an active transporter of NH_3, i.e. ammonium is transported by an SH^+ or S/H^+ mechanism [23, 24]. In the SH^+ mechanism, NH_4^+ is transported as it is. In the S/H^+ mechanism, NH_3 and H^+ are separately transported. If one NH_3 is co-transported with one H^+, the S/H^+ mechanism is thermodynamically equivalent to the SH^+ mechanism. In the active transporter model, NH_4^+ can accumulate inside cells owing to the membrane potential (inside negative) because of the positive charge. In the active transporter model, φ is for that reason a function of membrane potential ($\Delta\psi$):

$$\varphi = \exp\left(\frac{-F \cdot \Delta\psi}{RT}\right), \cdots\cdots (S1.3\text{-}2)$$

where $\Delta\psi$ is the transmembrane electrical potential, F is the Faraday constant, R is the gas constant, T is the absolute temperature. Given $\Delta\psi$ = -150 mV, φ = 275 (or 313) at T = 310 K (or 303 K).

For the passive transporter model, we assume AmtB facilitates the passive transport of NH_3 [23, 24] (In this paper, we use “facilitated” passive transport to distinguish it clearly from *unmediated* passive NH_3 diffusion

through the cytoplasmic membrane). Thus, only the concentration gradient of NH_3 is the driving force of transport. At equilibrium, NH_4^+ can be accumulated in or expelled from cells if and only if the internal pH is lower or higher, respectively, than the external pH. Consequently, in the passive transporter model, φ is a function of the pH difference:

$$\varphi = \frac{[H^+_{int}]}{[H^+_{ext}]} = 10^{pH_{ext} - pH_{int}}, \quad \text{(S1.3-3)}$$

where $[H^+_{ext}]$ and $[H^+_{int}]$ are the extracellular and intracellular proton concentrations, respectively. pH_{ext} and pH_{int} are extracellular and intracellular pH, respectively. Given $pH_{int} = 7.6$, $\varphi = 0.25$ (or 0.63) at $pH_{ext} = 7.0$ (or 7.4).

1.4 Regulation of AmtB

In this paper, "GlnK" depicts the GlnK trimer which harbours three uridylylation sites. We modelled the (de)uridylylation rate equations as Bruggeman et al. did for GlnB [14] (with correction). The rate equation of the first uridylylation reaction is given by:

$$v_{utglnk1} = \frac{k_{catutglnk}[UTase][GlnKUMP_{0,cyt}][UTP]}{\left(1+\frac{[GLN]}{K_{utgln}}\right)\left(\begin{array}{l} K_{utiglnk}K_{ututp} + K_{ututp}\left([GlnKUMP_{0,cyt}] + \sum_{i=1}^{2}[GlnKUMP_i]\right) + K_{utglnk}[UTP] \\ +\left([GlnKUMP_{0,cyt}] + \sum_{i=1}^{2}[GlnKUMP_i]\right)\cdot[UTP] \\ +\frac{K_{utglnk}[UTP]\sum_{i=0}^{2}[GlnKUMP_{i+1}]}{K_{utiglnkump}} \\ +\frac{\left([GlnKUMP_{0,cyt}] + \sum_{i=1}^{2}[GlnKUMP_i]\right)\cdot[UTP][PPi]}{K_{utippi}} \end{array}\right)}, \quad \text{(S1.4-1)}$$

where $[GlnKUMP_{0,cyt}]$ is ununridylylated GlnK in the cytoplasm, i.e. $[GlnKUMP_{0,cyt}] = [GlnKUMP_0] - [GlnKAmtB]$. It should be noted that GlnK bound to AmtB is not accessible to UTase (cf. [25]). Similarly, the second and third uridylylation reactions are given by

$$v_{utglnk,j} = \frac{k_{catutglnk}[UTase][GlnKUMP_{j-1}][UTP]}{\left(1+\frac{[GLN]}{K_{utgln}}\right)\left(\begin{array}{l} K_{utiglnk}K_{ututp} + K_{ututp}\left([GlnKUMP_{0,cyt}] + \sum_{i=1}^{2}[GlnKUMP_i]\right) + K_{utglnk}[UTP] \\ +\left([GlnKUMP_{0,cyt}] + \sum_{i=1}^{2}[GlnKUMP_i]\right)\cdot[UTP] \\ +\frac{K_{utglnk}[UTP]\sum_{i=0}^{2}[GlnKUMP_{i+1}]}{K_{utiglnkump}} \\ +\frac{\left([GlnKUMP_{0,cyt}] + \sum_{i=1}^{2}[GlnKUMP_i]\right)\cdot[UTP][PPi]}{K_{utippi}} \end{array}\right)}, \quad \text{(S1.4-2)}$$

where $j = 2, 3$. The rate equation of the deuridylylation reaction is given by:

$$v_{urglnk,j} = \frac{k_{caturglnk}[UTase][GlnKUMP_j]}{\left(1+\frac{K_{urgln}}{[GLN]}\right)\left(K_{urglnkump} + \sum_{i=1}^{3}[GlnKUMP_i] + \frac{\sum_{i=1}^{3}[GlnKUMP_i]\cdot[UMP]}{K_{urump}}\right)}, \quad \text{(S1.4-3)}$$

where $j = 1, 2, 3$.

We do not explicitly include ATP hydrolysis by and subsequent conformational change of GlnK in the models because the kinetic details remain unclear. Instead, we model only binding between GlnK and 2-oxoglutarate, implicitly assuming that ATP/ADP binding to GlnK, ATP hydrolysis, and the conformation change are very fast. Let $GlnKUMP_0OG_0$ be completely deuridylylated and 2-oxoglutarate-unbound GlnK. Since a GlnK trimer harbors three 2-oxoglutarate-binding sites, the concentration of $GlnKUMP_0OG_0$ is given by:

$$[GlnKUMP_0OG_0] = \frac{[GlnKUMP_0]}{1 + \frac{3[OG]}{K_{glnkog1}} + \frac{3[OG]^2}{K_{glnkog1}K_{glnkog2}} + \frac{[OG]^3}{K_{glnkog1}K_{glnkog2}K_{glnkog3}}}, \quad \text{(S1.4-4)}$$

where $[GlnKUMP_0]$ represents the sum of all deuridylylated forms of GlnK ($GlnKUMP_0OG_0$, $GlnKUMP_0OG_1$, $GlnKUMP_0OG_2$, and $GlnKUMP_0OG_3$; see also Section 1.6). $GlnKUMP_0OG_0$ is the only GlnK form that can bind to AmtB.

The AmtB trimer binds the GlnK trimer in a one-to-one ratio. The concentration of the active form of AmtB is given by

$$[AmtB_{GlnKfree}] = \frac{1}{2}\left(\begin{array}{l} -[GlnKUMP_0OG_0] + [AmtB_{total}] - K_{glnkamtb} \\ + \sqrt{\begin{array}{l} \left([GlnKUMP_0OG_0] - [AmtB_{total}] + K_{glnkamtb}\right)^2 \\ +4K_{glnkamtb}[AmtB_{total}] \end{array}} \end{array} \right). \quad \text{(S1.4-5)}$$

1.5 Ammonium/ammonia (NH_x) transfer

The net NH_x transfer rate v_{net} (mM/min) is given by:

$$v_{net} = v_{amtb} + v_{diff}, \quad \text{(S1.5-1)}$$

where v_{amtb} is the rate of the AmtB-mediated ammonium transport given by Eq. (S1.3-1), and v_{diff} is the rate of unmediated NH_3 diffusion across the cytoplasmic membrane. Based on Fick's law of diffusion, v_{diff} is given by:

$$v_{diff} = \frac{P_{cm}A_{cell}}{V_{cell}}\left([NH_{3surf}] - [NH_{3int}]\right), \quad \text{(S1.5-2)}$$

where P_{cm} is the permeability coefficient of the cytoplasmic membrane (m/min), A_{cell} is the surface area of a cell (m^2), V_{cell} is the volume of a cell (m^3). $[NH_{3surf}]$ and $[NH_{3int}]$ are the NH_3 concentrations (mM) at the cell surface and in the cytoplasm, respectively.

The work of Kim and of Yuan are the most valuable sources of quantitative experimental data for kinetic modeling of the *E. coli* ammonium transport and assimilation network [1, 6]. Kim et al. (Kim hereafter) grew *E. coli* in a microfluidic chamber. Yuan et al. (Yuan hereafter) grew *E. coli* on top of filters on agarose media. Based on the experimental data from Kim [6], we calculated the NH_3 permeability coefficient of the cytoplasmic membrane (P_{cm}) and obtained P_{cm} = 0.077 m/min. With this value of P_{cm}, Yuan's experimental data can be explained only on the assumption that the NH_x concentration is lower at the cell surface than in the

bulk medium (see Section 5). This observation convinced us that the models require an additional diffusion barrier adjacent to the cell surface.

Generally, if stirring is slow or absent or if solution viscosity is high, the movement of dissolved molecules is limited [26], resulting in a decrease in the concentration at the cell surface compared to that in the bulk medium if cells consume the solutes faster than can be replenished from the medium. This probably applies to the cells in Yuan's experiments whenever they were growing on filters on top of solid agarose medium under N-limiting condition: NH_x has to move from the solid agarose medium to the backside of the filter and from there through the filter to the cells growing on top. Thus, the NH_x transfer from the medium to the intracellular space is determined not only by the permeability of the cytoplasmic membrane and by AmtB-mediated transport, but also by permeability of the diffusion barrier. Let v_{db} be the NH_x flux through the diffusion barrier. Assuming that the permeability coefficients for NH_3 and NH_4^+ are equal and that Fick's law of diffusion is applicable, we obtain:

$$v_{db} = k_{db}\left([NH_{x,ext}]-[NH_{x,surf}]\right), \cdots\cdots \text{(S1.5-3)}$$

where k_{db} is the mass transfer capacity coefficient for NH_x diffusion through the diffusion barrier (min^{-1}). $[NH_{x,ext}]$ and $[NH_{x,surf}]$ are the NH_x concentrations (mM) in the bulk medium and at the cell surface, respectively. See also Section 6.

1.6 Details on GlnK-, GlnB-, AmtB- and GS-related variables

Since GlnK, GlnB, AmtB, and GS form complexes and/or are covalently modified, there are many possible molecular species related to these proteins. In this section, we explain how the concentrations of the "child" variables add up to the total concentrations.

$[GlnK_{total}]$ denotes the total concentration of GlnK and consists of four child variables.

$$[GlnK_{total}] = [GlnKUMP_0]+[GlnKUMP_1]+[GlnKUMP_2]+[GlnKUMP_3], \cdots\cdots \text{(S1.6-1)}$$

where $[GlnKUMP_0]$, $[GlnKUMP_1]$, $[GlnKUMP_2]$, and $[GlnKUMP_3]$ denote deuridylylated, singly uridylylated, doubly uridylylated, and triply uridylylated GlnKs, respectively. On its turn, $[GlnKUMP_0]$ consists of four child variables:

$$[GlnKUMP_0] = [GlnKUMP_0OG_0]+[GlnKUMP_0OG_1]+[GlnKUMP_0OG_2] +[GlnKUMP_0OG_3], \cdots\cdots \text{(S1.6-2)}$$

$[GlnKUMP_0OG_0]$ denotes deuridylylated 2-oxoglutarate-unbound GlnK. $[GlnKUMP_0OG_1]$, $[GlnKUMP_0OG_2]$, and $[GlnKUMP_0OG_3]$ denote deuridylylated GlnK with one, two, three molecules of 2-oxoglutarate, respectively. Similarly, each of $[GlnKUMP_1]$, $[GlnKUMP_2]$, and $[GlnKUMP_3]$ consists of four child variables:

$$[GlnKUMP_1] = [GlnKUMP_1OG_0]+[GlnKUMP_1OG_1]+[GlnKUMP_1OG_2] +[GlnKUMP_1OG_3], \cdots\cdots \text{(S1.6-3)}$$

$$[GlnKUMP_2] = [GlnKUMP_2OG_0]+[GlnKUMP_2OG_1]+[GlnKUMP_2OG_2] +[GlnKUMP_2OG_3], \cdots\cdots \text{(S1.6-4)}$$

$$[GlnKUMP_3] = [GlnKUMP_3OG_0] + [GlnKUMP_3OG_1] + [GlnKUMP_3OG_2] + [GlnKUMP_3OG_3], \cdots\cdots\cdots\cdots\cdots \text{(S1.6-5)}$$

Deuridylylated 2-oxoglutarate-unbound GlnK is the species that can bind to AmtB [10, 27], and thus

$$[GlnKUMP_0OG_0] = [GlnKUMP_0OG_{0,cyt}] + [GlnKAmtB], \cdots\cdots\cdots\cdots\cdots \text{(S1.6-6)}$$

where [$GlnKUMP_0OG_{0,cyt}$] denotes deuridylylated, 2-oxoglutarate-unbound, and AmtB-unbound (thus, cytoplasmic) GlnK. [GlnKAmtB] denotes GlnK-AmtB complex.

Similarly to GlnK, [$GlnB_{total}$] denotes the total concentration of GlnB and consists of four child variables, and each child variable consists again of four child variables. However, in contrast to GlnK, GlnB is assumed not to bind to AmtB.

$$[GlnB_{total}] = [GlnBUMP_0] + [GlnBUMP_1] + [GlnBUMP_2] + [GlnBUMP_3], \cdots\cdots\cdots\cdots\cdots \text{(S1.6-7)}$$

$$[GlnBUMP_0] = [GlnBUMP_0OG_0] + [GlnBUMP_0OG_1] + [GlnBUMP_0OG_2] + [GlnBUMP_0OG_3], \cdots\cdots\cdots\cdots\cdots \text{(S1.6-8)}$$

$$[GlnBUMP_1] = [GlnBUMP_1OG_0] + [GlnBUMP_1OG_1] + [GlnBUMP_1OG_2] + [GlnBUMP_1OG_3], \cdots\cdots\cdots\cdots\cdots \text{(S1.6-9)}$$

$$[GlnBUMP_2] = [GlnBUMP_2OG_0] + [GlnBUMP_2OG_1] + [GlnBUMP_2OG_2] + [GlnBUMP_2OG_3], \cdots\cdots\cdots\cdots\cdots \text{(S1.6-10)}$$

$$[GlnBUMP_3] = [GlnBUMP_3OG_0] + [GlnBUMP_3OG_1] + [GlnBUMP_3OG_2] + [GlnBUMP_3OG_3], \cdots\cdots\cdots\cdots\cdots \text{(S1.6-11)}$$

[$AmtB_{total}$] denotes the total concentration of AmtB and consists of two child variables:

$$[AmtB_{total}] = [AmtB_{GlnKfree}] + [GlnKAmtB], \cdots\cdots\cdots\cdots\cdots \text{(S1.6-12)}$$

where [$AmtB_{GlnKfree}$] is GlnK-unbound AmtB which is capable of transporting ammonium, and [GlnKAmtB] is GlnK-AmtB complex which does not transport ammonium.

[GS_{total}] is the total concentration of GS and consists of deadenylylated and adenylylated forms of GS:

$$[GS_{total}] = [GS] + [GSAMP], \cdots\cdots\cdots\cdots\cdots \text{(S1.6-13)}$$

where [GS] denotes the deadenylylated GS and [GSAMP] denotes the adenylylated GS.

2 Simulation Settings

In this section, we explain in detail how we simulated Yuan's [1], Kim's [6] and Radchenko's [10] experiments. Steady-state growth of cells in the presence of a range of ammonium concentrations (4 μM to 1 mM) in Kim's microfluidic chamber is qualitatively quite different from growth of cells on Yuan's filters on top of solid agarose media confronted with a 10 mM ammonium pulse or metabolizing cells in Radchenko's liquid media facing a 200 μM ammonium pulse. Nevertheless we sought to change the models for fitting these disparate training data sets as parsimoniously as possible: All the three papers were accommodated with one and the same set of parameters except for the paper-specific necessary adjustments (see Sections 2.1, 2.2, and 2.3). Some parameters needed to be adjusted because they depend on the specific experimental culture conditions.

We assumed that the expression levels of GlnK, AmtB, GS, GDH, and GOGAT are different between Yuan's, Kim's and Radchenko's experiments. In addition, the authors performed experiments in growth media with a different temperature and pH. In the following sections, [$GlnK_{total}$], [GS_{total}], [$AmtB_{total}$], V_{gdh}, V_{gog}, τ_0, $K_{growthglu}$, $K_{growthgln}$, T, pKa, and pH_{ext} are referred to as experiment-*dependent* parameters. The following 13 parameters are used only in one or two experiments: τ_0, $K_{growthglu}$, $K_{growthgln}$, GLU_{demn}, GLU_{demf}, GLN_{demf}, GLN_{demf}, k_{db}, κ, N_{int}^*, [OG_{basal}], V_{medium}, and $[NH_{x,ext}]_{t<0}$. These parameters are referred to as experiment-*specific* parameters. The extracellular NH_4^+ and the intracellular 2-oxoglutarate, ATP, and ADP are sometimes used as dynamic model inputs, i.e. their concentrations are changed *within* an experiment.

2.1 Yuan's experiments

E. coli K12 strain NCM3722 was used as wild type, all mutants strain were all isogenic with NCM3722 [1]. Cells grown under ammonium-limited condition before ammonium-upshift are assumed to have expressed GS, AmtB, and GlnK and that the expression levels have remained unchanged after N-upshift because of the short simulation duration (up to 30 min after N-upshift). In Yuan's experiments, we assumed that NH_x concentration at the cell surface can be different from that in the bulk medium as described in Section 1.5. As ATP, ADP, NADPH, and $NADP^+$ concentrations did not change upon the ammonium-upshift (Fig. 3 and Supp. Fig. 4 of [1]), these cofactors are modeled as constants. For ATP, ADP and NADPH we used the concentrations as measured for unrestricted growth on filters with glucose and ammonium [28]; however for NADP, we used a higher value to obtain an NADPH/NADP ratio that lies within the commonly observed range (0.8-3.0) [29-32].

Parameter modifications needed to simulate various mutants are shown in **Table S5**. In order to simulate mutant strains, we changed V_{max} or protein concentrations of corresponding enzymes. Note that ΔGOGAT requires a change not only in V_{gog} but also in V_{gdh} and [GS_{total}] because the enzyme expression level of GDH is tripled in this mutant strain and that of GS is reduced by half compared to wild type [1]. Qualitatively comparable changes were observed by Kumar and Shimizu in *gltB* and *gltD* mutants of strain BM25113 [33]. It was shown that a *Salmonella typhimurium* ΔGOGAT mutant (Δ*gltB*) did not express AmtB [34]. Thus, we set [$GlnK_{total}$] = 0 and [$AmtB_{total}$] = 0. It should be noted though that Yuan's ΔGOGAT is Δ*gltD*, and we assume Δ*gltB* and Δ*gltD* behave similarly. Since the reported specific growth rates vary for different strains (Supp. Fig. 2 of [1]), we used different τ_0 values for mutants (**Table S4**).

2.1.1 Extracellular NH_x concentration

The extracellular NH_x concentrations used for simulations are summarized in **Table S6**. We explain below how we obtained these concentrations.

The extracellular NH_x concentrations of wild type and ΔGOGAT in 13x N-upshift experiments are given in Fig. 2B of [1]: Before N-upshift, solid agarose media containing 0.75 and 1 mM NH_x for wild type and ΔGOGAT, respectively, were used. Then, the filters with cells were transferred to solid agarose media containing 10 mM NH_x.

Since NH_x concentrations in media for other mutant strains are not shown in [1], we had to estimate them. For ΔGDH, ΔATase and ΔAmtB in 13x N-upshift experiments, we assumed the extracellular NH_x concentration before N-upshift was the same as that for wild type, because Yuan reported that before N-upshift these mutants and the wild type showed indistinguishable metabolome profiles.

Similarly, we estimated NH_x concentrations for 3x N-upshift and N-downshift. For 3x N-upshift and N-downshift, Yuan grew *E. coli* in the same way as for 13x N-upshift before filter transfer. Thus, we assumed the extracellular NH_x concentrations for 3x N-upshift and N-downshift were the same as for 13x N-upshift before filter transfer. In 3x N-upshift experiment, *E. coli*-inoculated filters were transferred to media with 2 mM of NH_x [1]. To induce N-downshift, Yuan transferred *E. coli*-inoculated filters to NH_x-free media. The transferred filters contained app. 0.05 mM of NH_x (Supp. Fig. 6 of [1]). Therefore, we assumed 0.05 mM extracellular NH_x was present after N-downshift.

<u>2.1.2 2-Oxoglutarate concentration</u>

Yuan measured 2-oxoglutarate with respect to time (Fig. 4 of [1]), and provided the numerical data as Supp. Dataset of [1] (msb200960-s4.xls). For ΔAmtB, the 2-oxoglutarate concentration was taken from Supp. Fig. 5 of [1]. We utilized these data with linear interpolation.

2.2 Kim's experiments

E. coli K12 strain NCM3722 was used as wild type, the *glnKamtB* mutant strain (ΔamtB) was isogenic with NCM3722 [6]. AmtB, GlnK, and GS concentrations depend on the extracellular NH_4^+ concentration, i.e. they are functions of the extracellular NH_4^+ concentration (see Section 2.2.1). V_{gdh} and V_{gog} were estimated in our study. The reference value of τ_0 is obtained by dividing τ_0 for wild type of Yuan's experiments by two, because the specific growth rate in Kim's experiments (0.8 h^{-1} for glucose as carbon source) is twice as high as in Yuan's experiments (0.36 h^{-1} after 13x N-upshift). Since liquid media were used, NH_x molecules were easily transferred from the medium to the cell surface. Therefore, NH_x concentration at the cell surface was taken to be equal to that in the bulk medium.

Parameter modifications needed to simulate various experimental conditions are shown in **Table S7**. Different carbon sources result in different τ_0 values. When glucose is the carbon source, wild type grows at 0.80 h^{-1}. For glycerol, it is 0.62 h^{-1}. Therefore, to simulate the glycerol culture, we multiplied τ_0 by 1.29 (= 0.80 / 0.62). Likewise, to simulate the G6P + gluconate culture, we multiplied τ_0 by 0.89 (= 0.8 / 0.9).

<u>2.2.1 AmtB, GlnK, and GS concentrations</u>

The *amtB* and *glnA* promoter activities with respect to the extracellular NH_4^+ concentration were reported in Supp. Table 6-11 of [6], where the activities were presented as fluorescence intensities of GFP and mCherry, respectively. In order to develop continuous functions that yield promoter activities with respect to the extracellular NH_4^+ concentration, we took an approach similar to that shown in [6]. We fitted the following

Hill function to the *amtB* promoter activity data:

$$P_{AmtB}\left(P_{AmtB,std}, H_{AmtB}, K_{AmtB}, f_{AmtB}; [NH_{4ext}^{+}]\right) = P_{AmtB,std}\,\frac{f_{AmtB} + \left([NH_{4ext}^{+}]/K_{AmtB}\right)^{H_{AmtB}}}{1+\left([NH_{4ext}^{+}]/K_{AmtB}\right)^{H_{AmtB}}}, \qquad \text{(S2.2.1-1)}$$

where $P_{AmtB,std}$ is the promoter activity at a relatively high extracellular NH_4^+ level (e.g. the value at 10 mM extracellular NH_4^+, which is the highest concentration provided in Supp. Table 6-11 of [6]. If the promoter activity at 10 mM is zero, the activity at 1 mM is used). H_{AmtB}, K_{AmtB}, and f_{AmtB} are Hill coefficient, half-inhibition concentration, maximal fold change, respectively. These parameters differ for strains and carbon sources. The fitted parameters are provided in **Table S8**.

Using the above promoter activity function, we obtain the AmtB concentration by assuming that the AmtB concentration is proportional to the promoter activity and that the promoter activity of wild type cells grown on medium containing 4 μM of NH_4^+ as nitrogen source and ample glucose as carbon source corresponds to a certain AmtB concentration ($[AmtB_{total}]^*$). Taken together, AmtB concentration at arbitrary extracellular NH_4^+ concentration is then obtained by:

$$[AmtB_{total}] = [AmtB_{total}]^*\,\frac{P_{AmtB}\left(P_{AmtB,std}, H_{AmtB}, K_{AmtB}, f_{AmtB}; [NH_{4ext}^{+}]\right)}{P_{AmtB}\left(P_{AmtB,std}^{*}, H_{AmtB}^{*}, K_{AmtB}^{*}, f_{AmtB}^{*}; [NH_{4ext}^{+}]^{*}\right)}, \qquad \text{(S2.2.1-2)}$$

where the asterisks show the "reference" values (wild type grown on 4 μM of NH_4^+ and with glucose as carbon source): $[NH_4^+{}_{ext}]^* = 4$ μM, $P_{AmtB,std}^* = 6.3$, $H_{AmtB}^* = 1.9$, $K_{AmtB}^* = 0.058$, $f_{AmtB} = 28$ (**Table S8**). $[AmtB_{total}]^*$ is estimated in our study (**Table S4**). Using Eq (S2.2.1-2) and **Table S8**, AmtB concentration at arbitrary extracellular NH_4^+ concentrations can be obtained. For example, if $[AmtB_{total}]^* = 1.5$ μM, wild type cells growing with G6P + gluconate contain 1.0 μM total AmtB at 100 μM extracellular NH_4^+ ($P_{AmtB,std} = 11.6$, $H_{AmtB} = 1.6$, $K_{AmtB} = 0.079$ μM, $f_{AmtB} = 23$, $[NH_4^+{}_{ext}] = 100$ μM). Since *glnK* gene is located on the same operon as *amtB*, we assumed the GlnK concentration to be proportional to the *amtB* promoter activity:

$$[GlnK_{total}] = [GlnK_{total}]^*\,\frac{P_{AmtB}\left(P_{AmtB,std}, H_{AmtB}, K_{AmtB}, f_{AmtB}; [NH_{4ext}^{+}]\right)}{P_{AmtB}\left(P_{AmtB,std}^{*}, H_{AmtB}^{*}, K_{AmtB}^{*}, f_{AmtB}^{*}; [NH_{4ext}^{+}]^{*}\right)}. \qquad \text{(S2.2.1-3)}$$

$[GlnK_{total}]^*$ is estimated in our study (**Table S4**). Similarly to AmtB, we fitted the following Hill function to the GS promoter activity data of [6]:

$$P_{GS}\left(P_{GS,std}, H_{GS}, K_{GS}, f_{GS}; [NH_{4ext}^{+}]\right) = P_{GS,std}\,\frac{f_{GS} + \left([NH_{4ext}^{+}]/K_{GS}\right)^{H_{GS}}}{1+\left([NH_{4ext}^{+}]/K_{GS}\right)^{H_{GS}}}, \qquad \text{(S2.2.1-4)}$$

We assumed that the promoter activity of wild type cells grown on medium containing 4 μM of NH_4^+ as nitrogen source and ample glucose as carbon source corresponds to a certain GS concentration ($[GS_{total}]^*$). GS concentration at arbitrary extracellular NH_4^+ concentrations are obtained by:

$$[GS_{total}] = [GS_{total}]^*\,\frac{P_{GS}\left(P_{GS,std}, H_{GS}, K_{GS}, f_{GS}; [NH_{4ext}^{+}]\right)}{P_{GS}\left(P_{GS,std}^{*}, H_{GS}^{*}, K_{GS}^{*}, f_{GS}^{*}; [NH_{4ext}^{+}]^{*}\right)}, \qquad \text{(S2.2.1-5)}$$

where $[NH_{4ext}]^* = 4$ μM, $P_{GS,std} = 23.5$, $H_{GS} = 0.50$, $K_{GS} = 0.42$ μM, $f_{GS} = 2.6$. $[GS_{total}]^*$ is estimated in our study (**Table S4**).

2.2.2 Extracellular NH_4^+ concentration

The extracellular NH_4^+ is given as indicated on the abscissas of **Figure 4 and 6**.

2.2.3 2-Oxoglutarate concentration

The 2-oxoglutarate concentration was not reported in [6]. Therefore, we had to develop a function of the 2-oxoglutarate concentration with respect to the NH_4^+ concentration. Kim predicted a change in 2-oxoglutarate level with respect to the extracellular NH_4^+ (Figure 5C of [6]). The 2-oxoglutarate level is insensitive to the extracellular NH_4^+ level if the extracellular NH_4^+ is higher than a certain level indicated as N_{ext}^*, i.e. the external NH_4^+ level below which AmtB is activated. If the extracellular NH_4^+ is less than N_{ext}^*, the 2-oxoglutarate level increases as the extracellular NH_4^+ decreases. Kim also provided an equation that relates the 2-oxoglutarate level to the intracellular NH_4^+ concentration (Eq. (S29) of [6]). When the intracellular NH_4^+ is below N_{int}^*, i.e. the internal NH_4^+ level below which AmtB is activated, the 2-oxoglutarate level increases as the intracellular NH_4^+ decreases. Based on Kim's predictions, we developed a function of the 2-oxoglutarate concentration with respect to the intracellular NH_4^+ concentration:

$$[OG] = \begin{cases} \kappa\left(1 - \dfrac{[NH_{4int}^+]}{N_{int}^*}\right) + [OG_{basal}], & \text{if } [NH_{4int}^+] < N_{int}^* \\ [OG_{basal}], & \text{otherwise} \end{cases} \quad \cdots\cdots\cdots\cdots\cdots\cdots\cdots\cdots\cdots\cdots\text{(S2.2.3-1)}$$

In Eq. (S2.2.3-1), there are three parameters N_{int}^*, $[OG_{basal}]$, and κ. N_{int}^* depends on the carbon source: 0.019 mM for glycerol, 0.033 mM for glucose, and 0.057 mM for G6P + gluconate (Table S5 of [6]). $[OG_{basal}]$ and κ are independent of carbons sources and strains. $[OG_{basal}]$ is the 2-oxogluratate concentration under N-rich condition, and κ is the proportional constant. They are class II parameters, and their reference values are 0.7 mM and 10.9 mM, respectively. These reference values were chosen to make the range of [OG] congruent with that reported in [1, 35].

2.3 Radchenko's experiments

E. coli strain GT1000 (*ΔglnKamtB*) harbouring plasmid pAD2 (*glnK His6amtB*) or pADY51A (*glnK_Y51A His6amtB*) were used as the wild type and the GlnK Y51A mutant, respectively; the expression of WT GlnK or Y51A GlnK and his-tagged AmtB were under the control of the native promoter of the *glnKamtB* operon [10]. Cells grown under N-limited condition before the ammonium-upshift are assumed to have expressed GS, AmtB, and GlnK and that the expression levels have remained unchanged after N-upshift during the short duration of the experiment (20 min) after the shift. NH_4^+ concentration at the cell surface was taken to be equal to that in the bulk medium. The cells are expected to be severely N-limited before the N-upshift. Therefore, we assumed that the cells did not grow in Radchenko's experiments after the N-upshift ($\mu = 0$, $v_{gludemn} = 0$, $v_{gludemf} = 0$, $v_{glndemn} = 0$, and $v_{glndemf} = 0$, but were still able to metabolise ammonium. In order to simulate GlnK Y51A mutant whose GlnK cannot be uridylylated, we set k_{cat} of the uridylyl transfer reaction of UTase to zero

($k_{catutglnk}$ = 0).

2.3.1 Extracellular NH_x concentration

Radchenko et al. (Radchenko hereafter) grew *E. coli* in liquid media with glutamine as nitrogen source, and then increased NH_x concentration to 200 μM [10]. They did not measure extracellular ammonium concentrations. Therefore, we modeled the extracellular NH_x concentration as a model variable. We assumed that the extracellular NH_x concentration before the N-upshift is constant and very low. At the N-upshift, the extracellular NH_x is shifted to 200 μM. After the N-upshift, the extracellular NH_x decreases according to the following differential equation:

$$\frac{d[NH_{x,ext}]}{dt} = -(v_{amtb} + v_{diff})\frac{V_{cell}}{V_{medium}}, \quad \text{(S2.3.1-1)}$$

where v_{amtb} is the rate of the AmtB-mediated ammonium transport [Eq. (S1.3-1)], v_{diff} is the rate of unmediated NH_3 diffusion across the cytoplasmic membrane [Eq. (S1.5-2)]. V_{cell} is the cell volume. V_{medium} is the medium volume per cell, which is estimated as follows: Before the N-upshift, OD_{600} = 1.3 – 1.4 [10]. Using OD_{600} = 1.35 and 1.1 x 10^9 cells per ml medium per OD_{600} [25], we obtain V_{medium} = 6.7 x 10^{-13} L-medium/cell. The NH_x concentration before the N-upshift ($[NH_{x,ext}]_{t<0}$) is a class III parameter.

2.3.2 2-Oxoglutarate, ATP, and ADP concentrations

Radchenko did not measure 2-oxoglutarate, ATP, and ADP in [10]. However, they conducted a similar experiment where they did measure these metabolites (Figure 2A and Figure 3A of [36]). We used the latter experimental data for the wild type in our simulation. For the GlnK Y51A mutant, those metabolites have not been measured. For ATP and ADP, we used the same values as for the wild type. We estimated the time evolution of 2-oxoglutarate for the GlnKY51A mutant.

3 Model Plausibility

In parameter estimation, as the reference values, we use the experimentally measured values, computationally estimated values, or rough guesses. The reference values are not "true" values; however, if estimated values are far from the reference values, the estimated values and thus the model using these values can be considered less realistic. In this section, we explain how we quantify the plausibility of individual parameter values and of the model as a whole. Here, we introduce q_i which is the natural logarithm of the ratio of p_i to p_i^*:

$$q_i = \ln\frac{p_i}{p_i^*}. \quad \text{(S3-1)}$$

We assume that the probability density of q_i follows a normal distribution with the mean of zero. The probability density function (*PDF*) for p_i is:

$$PDF_i(p_i) = \frac{1}{\sqrt{2\pi\sigma_i^2}}\exp\left(-\frac{\left[\ln(p_i / p_i^*)\right]^2}{2\sigma_i^2}\right), \quad \text{(S3-2)}$$

where σ_i is the standard deviation for q_i. Here, we define parameter plausibility (*PP*) for the estimated value of the *i*th parameter:

$$PP_i(p_i) = \frac{PDF_i(p_i)}{PDF_i(p_i^*)}$$

$$= \frac{\frac{1}{\sqrt{2\pi\sigma_i^2}} \exp\left(-\frac{\left[\ln(p_i / p_i^*)\right]^2}{2\sigma_i^2} \right)}{\frac{1}{\sqrt{2\pi\sigma_i^2}} \exp\left(-\frac{\left[\ln(p_i^* / p_i^*)\right]^2}{2\sigma_i^2} \right)} \quad \text{(S3-3)}$$

$$= \exp\left(-\frac{\left[\ln(p_i / p_i^*)\right]^2}{2\sigma_i^2} \right)$$

PP_i indicates how realistic an estimated parameter p_i is. If $p_i = p_i^*$, then $\ln(p_i/p_i^*) = 0$ and $PP_i = 1$. As p_i deviates from p_i^*, PP_i decreases to zero. Next, we define model plausibility MP as the product of parameter plausibilities:

$$MP(\mathbf{p}) = \prod_{i=1}^{n} PP_i(p_i)$$

$$= \prod_{i=1}^{n} \exp\left(-\frac{\left[\ln(p_i / p_i^*)\right]^2}{2\sigma_i^2} \right), \quad \text{(S3-4)}$$

where $\mathbf{p} = (p_1, \ldots, p_n)$ is the parameter vector, and n is the number of parameters. MP indicates how realistic the estimated parameter set and thus also the corresponding model is. If $p_i = p_i^*$ for all i, $MP = 1$, which is the maximum of MP. If $p_i << p_i^*$ or $p_i^* << p_i$ for one or more parameters, $MP \approx 0$. In principle, if just one estimated parameter is far from its reference value it might already substantially lower the model plausibility. For example, if $\sigma_i = \ln(2)$ for all i, $p_1 = 17.2\, p_1^*$, and $p_i = p_i^*$ for all i ($i \neq 1$), then $MP = 2.2 \times 10^{-4}$. If $p_j = 7.47\, p_j^*$ ($j = 1, 2$) and $p_i = p_i^*$ for all i ($i \neq 1, 2$), then $MP = 2.2 \times 10^{-4}$. For the rationale for our implementation of model plausibility, see Section 14.

4 Parameter Estimation

As described in Methods in the main text, we formulated our variable fitting and parameter estimation problem as a constrained optimization problem, and employed a genetic algorithm (GA) to solve it with the aid of a supercomputer. First, we elaborate on the objective function and the constraint functions used to fit experimental data. Next, we provide a brief description of the GA we employed.

4.1 Objective function

We define the objective function f as the natural logarithm of the inverse of model plausibility:

$$f(\mathbf{p}) = \ln\left[MP(\mathbf{p})^{-1}\right]$$

$$= \sum_{i=1}^{n} \frac{\left[\ln(p_i / p_i^*)\right]^2}{2\sigma_i^2}, \quad \text{(S4.1-1)}$$

$$= \sum_{i=1}^{n} \lambda_i \left(\ln \frac{p_i}{p_i^*}\right)^2$$

where

$$\lambda_i = \frac{1}{2\sigma_i^2}. \quad \text{(S4.1-2)}$$

$\mathbf{p} = (p_1, \ldots, p_n)$ is the parameter vector, n is the number of parameters, and p_i^* is the reference value of the ith parameter. If $p_i = p_i^*$ for all i, $f = 0$. Eq. (S4.1-1) is a general expression of model plausibility-based objective function. In this study, we categorize parameters into three classes and assign different λ_i values for each class, and thus Eq. (S4.1-1) can be written as:

$$f(\mathbf{p}) = \lambda_I \sum_{p_i \in \text{Class } I} \left(\ln \frac{p_i}{p_i^*}\right)^2 + \lambda_{II} \sum_{p_i \in \text{Class } II} \left(\ln \frac{p_i}{p_i^*}\right)^2 + \lambda_{III} \sum_{p_i \in \text{Class } III} \left(\ln \frac{p_i}{p_i^*}\right)^2, \quad \text{(S4.1-3)}$$

where λ_j ($j = I, II, III$) is the class-related penalty weight for a parameter change ($\lambda_I > \lambda_{II} > \lambda_{III} \geq 0$). We use $\lambda_I = 1.0407$, $\lambda_{II} = 0.1930$, and $\lambda_{III} = 0$. These values correspond to $\sigma_i = \ln(2) = 0.6931$, $\sigma_i = \ln(5) = 1.6094$, and $\sigma_i \to \infty$, respectively [see Eq. (S4.1-2)]. That is, PDF_i for class I parameters gives probability of 68% for $1/2 < p_i/p_i^* < 2$, and that for class II gives 68% probability for $1/5\ p_i/p_i^* < 5$ [see Eq. (S3-2)]. We use $\lambda_{III} = 0$, that is, we allow class III parameters to change freely because class III parameters have not been measured, and it is difficult to guess their values. In parameter estimation, GA tries to minimize f, i.e. maximize MP. The concept of our parameter estimation is analogous to maximum likelihood estimation. Actually, model plausibility is the ratio of the likelihood for $\mathbf{p}$ to that for $\mathbf{p}^*$. Therefore, maximization of MP is equal to maximization of the likelihood.

Two of the authors of this paper used an objective function similar to Eq. (S4.1-3) in the previous work [37]. The difference is that the objective function in the present work employs "squared" and "natural logarithm" [Eq. (S4.1-3)] while "absolute" and "common logarithm" were used in the previous work. The difference is subtle but important. Eq. (S4.1-3) is derived based on model plausibility while the objective function in the previous work was just an empirical formula, i.e. without theoretical background.

4.2 Constraint functions

In a GA, we sequentially simulated Yuan's, Kim's and Radchenko's experiments for each model candidate in order to evaluate to what extent the model candidate was realistic. Model reality is quantified by the objective function f and the constraint violation γ (see Methods in the main text). In the following sub-sections, the details of the constraint functions are made explicit.

4.2.1 General description of constraint functions

Constraint functions (except for auxiliary constraint constraints $g_{34\text{-}52}$) are expressed as one of the following four general forms of constraint functions.

Type 1 constraint function is used to evaluate fitting to time evolution data:

$$gfun_1(\mathbf{x}^{sim}, \mathbf{x}^{exp}, n, \varepsilon) = \frac{1}{n}\sum_{i=1}^{n}\left(\frac{x_i^{sim} - x_i^{exp}}{x_i^{exp}}\right)^2 - \varepsilon^2, \qquad \text{(S4.2.1-1)}$$

where $\mathbf{x}^{sim} = (x_1^{sim},\ldots, x_n^{sim})$ is the simulated variable vector, and x_i^{sim} indicates the simulated variable at the ith time point. $\mathbf{x}^{exp} = (x_1^{exp},\ldots, x_n^{exp})$ is the experimentally measured variable vector, and x_i^{exp} indicates the experimentally measured variable at the ith time point. n is the number of time points. ε is the allowable error. Depending on the level of uncertainty of experimentally measured variables, we use different values for ε: $\varepsilon_I = 0.05$, $\varepsilon_{II} = 0.1$, and $\varepsilon_{III} = 0.2$. If $gfun_1 \le 0$, the fitting is considered sufficient.

Type 2 constraint function is used to evaluate how close the simulated and experimentally measured values are:

$$gfun_2(x^{sim}, x^{\exp}, \varepsilon) = \begin{cases} 0 & \text{if } (1-\varepsilon)x^{\exp} \le x^{sim} \le (1+\varepsilon)x^{\exp} \\ \left(\frac{x^{sim} - (1-\varepsilon)x^{\exp}}{x^{\exp}}\right)^2 & \text{if } x^{sim} < (1-\varepsilon)x^{\exp} \\ \left(\frac{x^{sim} - (1+\varepsilon)x^{\exp}}{x^{\exp}}\right)^2 & \text{otherwise} \end{cases}, \qquad \text{(S4.2.1-2)}$$

where x^{sim}, x^{exp}, and ε are the simulated variable, measured variable, and allowable error, respectively.

Type 3 constraint function imposes a penalty if the simulated value (x^{sim}) is smaller than a certain lower bound (x^{lb}).

$$gfun_3(x^{sim}, x^{lb}) = \begin{cases} 0 & \text{if } x^{lb} \le x^{sim} \\ \left(\frac{x^{sim} - x^{lb}}{x^{lb}}\right)^2 & \text{otherwise} \end{cases}. \qquad \text{(S4.2.1-3)}$$

Type 4 constraint function imposes a penalty if the simulated value (x^{sim}) is larger than a certain upper bound (x^{ub}).

$$gfun_4(x^{sim}, x^{ub}) = \begin{cases} 0 & \text{if } x^{sim} \le x^{ub} \\ \left(\frac{x^{sim} - x^{ub}}{x^{ub}}\right)^2 & \text{otherwise} \end{cases}. \qquad \text{(S4.2.1-4)}$$

4.2.2 Constraint functions for Yuan's experiments

The first constraint function evaluates fitting with respect to glutamate for wild type in the 13x N-upshift.

$$g_1 = \frac{1}{n_{timepoint}} \sum_{i=1}^{n_{timepoint}} \left(\frac{[GLU]_i^{Yuan,WT} - [GLU]_i^{Yuan,WT*}}{[GLU]_i^{Yuan,WT*}} \right)^2 - \varepsilon_{III}^2, \quad \text{(S4.2.2-1)}$$

where the first term in the right-hand side of Eq. (S4.2.2-1) represents the summation of the squared residues between simulated and experimental values, and the second term in the right-hand side is an allowable error. This constraint is $gfun_1$ with ε_{III}. g_1 allows on average 20 % deviation between simulated and experimental values. $[GLU]_i^{Yuan,WT}$ and $[GLU]_i^{Yuan,WT*}$ are simulated and experimental values, respectively. The experimental values were obtained from the sheet "training set (fig. 4)" of msb200960-s4.xls of [1]. In contrast to Fig. 4 of [1], all data points from t = -15 to 30 min were used, thus $n_{datapoint}$ = 14. The superscript denotes that the variable applies to the wild type in Yuan's experiments. The subscript is the index of the individual data points. If $g_1 \leq 0$, the fitting is considered sufficiently good. Similarly, g_2 evaluates fitting with respect to glutamine:

$$g_2 = \frac{1}{n_{datapoint}} \sum_{i=1}^{n_{datapoint}} \left(\frac{[GLN]_i^{Yuan,WT} - [GLN]_i^{Yuan,WT*}}{[GLN]_i^{Yuan,WT*}} \right)^2 - \varepsilon_{III}^2. \quad \text{(S4.2.2-2)}$$

Likewise, the constraints for the ΔGDH and the ΔGOGAT strains are:

$$g_3 = \frac{1}{n_{datapoint}} \sum_{i=1}^{n_{datapoint}} \left(\frac{[GLU]_i^{Yuan,\Delta GDH} - [GLU]_i^{Yuan,\Delta GDH*}}{[GLU]_i^{Yuan,\Delta GDH*}} \right)^2 - \varepsilon_{III}^2, \quad \text{(S4.2.2-3)}$$

$$g_4 = \frac{1}{n_{datapoint}} \sum_{i=1}^{n_{datapoint}} \left(\frac{[GLN]_i^{Yuan,\Delta GDH} - [GLN]_i^{Yuan,\Delta GDH*}}{[GLN]_i^{Yuan,\Delta GDH*}} \right)^2 - \varepsilon_{III}^2, \quad \text{(S4.2.2-4)}$$

$$g_5 = \frac{1}{n_{datapoint}} \sum_{i=1}^{n_{datapoint}} \left(\frac{[GLU]_i^{Yuan,\Delta GOGAT} - [GLU]_i^{Yuan,\Delta GOGAT*}}{[GLU]_i^{Yuan,\Delta GOGAT*}} \right)^2 - \varepsilon_{III}^2, \quad \text{(S4.2.2-5)}$$

$$g_6 = \frac{1}{n_{datapoint}} \sum_{i=1}^{n_{datapoint}} \left(\frac{[GLN]_i^{Yuan,\Delta GOGAT} - [GLN]_i^{Yuan,\Delta GOGAT*}}{[GLN]_i^{Yuan,\Delta GOGAT*}} \right)^2 - \varepsilon_{III}^2. \quad \text{(S4.2.2-6)}$$

According to Fig. 2A of [1], the wild type grows at $\mu = 0.19\ h^{-1}$ before the N-upshift. Thus, the simulated value of the specific growth rate ($\mu^{Yuan,WT,N\text{-}limit}$) should be close to that value. If the deviation of $\mu^{Yuan,WT,N\text{-}limit}$ from $0.19\ h^{-1}$ (= $0.0032\ min^{-1}$) is less than or equal to 10%, then no penalty is imposed. Otherwise, the squared relative deviation is used as the penalty, i.e. $gfun_2$ with ε_{II}.

$$g_7 = gfun_2(\mu^{Yuan,WT,N\text{-}limit}, 0.0032, \varepsilon_{II}). \quad \text{(S4.2.2-7)}$$

After the N-upshift, the wild type grows at $\mu = 0.36\ h^{-1}$ (= $0.0060\ min^{-1}$) and the constraint is similarly given by:

$$g_8 = gfun_2(\mu^{Yuan,WT,N\text{-}rich}, 0.0060, \varepsilon_{II}) . \cdots\cdots (S4.2.2\text{-}8)$$

According to the left of Supp. Fig. 2 of [1], ΔGDH grew at μ = 0.19 h^{-1} (0.0032 min^{-1}) before N-upshift and at μ = 0.24 h^{-1} (0.0040 min^{-1}) after N-upshift:

$$g_9 = gfun_2(\mu^{Yuan,\Delta GDH,N\text{-}limit}, 0.0032, \varepsilon_{II}) , \cdots\cdots (S4.2.2\text{-}9)$$

$$g_{10} = gfun_2(\mu^{Yuan,\Delta GDH,N\text{-}rich}, 0.0040, \varepsilon_{II}) . \cdots\cdots (S4.2.2\text{-}10)$$

According to the right of Supp. Fig. 2 of [1], ΔGOGAT grew at μ = 0.23 h^{-1} (0.0038 min^{-1}) before N-upshift and at μ = 0.45 h^{-1} (0.0075 min^{-1}) after N-upshift:

$$g_{11} = gfun_2(\mu^{Yuan,\Delta GOGAT,N\text{-}limit}, 0.0038, \varepsilon_{II}) , \cdots\cdots (S4.2.2\text{-}11)$$

$$g_{12} = gfun_2(\mu^{Yuan,\Delta GOGAT,N\text{-}rich}, 0.0075, \varepsilon_{II}) . \cdots\cdots (S4.2.2\text{-}12)$$

The ammonium assimilation flux (J_N) is 9.5 mM/min before N-upshift since the specific growth rate is 0.19 h^{-1} (assuming N_0 = 3 M).

$$J_N^{Yuan,WT,N\text{-}limit} = v_{gs}^{Yuan,WT,N\text{-}limit} + v_{gdh}^{Yuan,WT,N\text{-}limit} , \cdots\cdots (S4.2.2\text{-}13)$$

$$g_{13} = gfun_2(J_N^{Yuan,WT,N\text{-}limit}, 9.5, \varepsilon_{II}) . \cdots\cdots (S4.2.2\text{-}14)$$

Similarly, the ammonium assimilation flux (J_N) is 18 mM/min after N-upshift since the specific growth rate is 0.36 h^{-1} (assuming N_0 = 3M).

$$J_N^{Yuan,WT,N\text{-}rich} = v_{gs}^{Yuan,WT,N\text{-}rich} + v_{gdh}^{Yuan,WT,N\text{-}rich} , \cdots\cdots (S4.2.2\text{-}15)$$

$$g_{14} = gfun_2(J_N^{Yuan,WT,N\text{-}rich}, 18, \varepsilon_{II}) , \cdots\cdots (S4.2.2\text{-}16)$$

Most of glutamate production is achieved by GOGAT, both before and after N-upshift [2, 38]. According to [2], it is more than 85%. We use $gfun_3$ for g_{15} and g_{16}:

$$g_{15} = gfun_3(r_{GLU}^{Yuan,WT,N\text{-}limit}, 0.85) , \cdots\cdots (S4.2.2\text{-}17)$$

$$g_{16} = gfun_3(r_{GLU}^{Yuan,WT,N\text{-}rich}, 0.85) , \cdots\cdots (S4.2.2\text{-}18)$$

where $r_{GLU}^{Yuan,WT,N\text{-}limit}$ and $r_{GLU}^{Yuan,WT,N\text{-}rich}$ are given by:

$$r_{GLU}^{Yuan,WT,N\text{-}limit} = \frac{2v_{gog}^{Yuan,WT,N\text{-}limit}}{v_{gdh}^{Yuan,WT,N\text{-}limit} + 2v_{gog}^{Yuan,WT,N\text{-}limit}} , \cdots\cdots (S4.2.2\text{-}19)$$

$$r_{GLU}^{Yuan,WT,N\text{-}rich} = \frac{2v_{gog}^{Yuan,WT,N\text{-}rich}}{v_{gdh}^{Yuan,WT,N\text{-}rich} + 2v_{gog}^{Yuan,WT,N\text{-}rich}} \cdot \cdots \quad \text{(S4.2.2-20)}$$

4.2.3 Constraint functions for Kim's experiments

According to the middle figure of Fig. 3A of [6], wild type cells with glucose grow at 0.80 h^{-1} (0.0133 min^{-1}) for 4 - 1000 μM of external NH_4^+. To avoid a rather broad range of the specific growth rates to be fitted to the experimental data, we took the lowest allowable error (5 %; ε_I).

$$g_{17} = gfun_2(\mu^{Kim,WT,[NH_{4ext}^+]=1000\mu M}, 0.0133, \varepsilon_I), \cdots \quad \text{(S4.2.3-1)}$$

$$g_{18} = gfun_2(\mu^{Kim,WT,[NH_{4ext}^+]=60\mu M}, 0.0133, \varepsilon_I), \cdots \quad \text{(S4.2.3-2)}$$

$$g_{19} = gfun_2(\mu^{Kim,WT,[NH_{4ext}^+]=20\mu M}, 0.0133, \varepsilon_I), \cdots \quad \text{(S4.2.3-3)}$$

$$g_{20} = gfun_2(\mu^{Kim,WT,[NH_{4ext}^+]=4\mu M}, 0.0133, \varepsilon_I). \cdots \quad \text{(S4.2.3-4)}$$

ΔAmtB grows at 0.80 h^{-1} (0.0133 min^{-1}) when the external NH_4^+ is 40 μM.

$$g_{21} = gfun_2(\mu^{Kim,\Delta AmtB,[NH_{4ext}^+]=40\mu M}, 0.0133, \varepsilon_I). \cdots \quad \text{(S4.2.3-5)}$$

The N-assimilation rate is 40 mM/min for the specific growth rate of 0.8 h^{-1} at 4 μM external NH_4^+.

$$g_{22} = gfun_2(J_N^{Kim,WT,[NH_{4ext}^+]=4\mu M}, 40, \varepsilon_{II}), \cdots \quad \text{(S4.2.3-6)}$$

where $J_N^{Kim,WT,[NH_{4ext}^+]=4\mu M}$ is given by

$$J_N^{Kim,WT,[NH_{4ext}^+]=4\mu M} = v_{gdh}^{Kim,WT,[NH_{4ext}^+]=4\mu M} + v_{gs}^{Kim,WT,[NH_{4ext}^+]=4\mu M}. \cdots \quad \text{(S4.2.3-7)}$$

4.2.4 Constraint functions for Radchenko's experiments

We semi-quantified blackness of the protein bands visible in Fig. 3A of [10] using ImageJ [39] and calculated the relative abundance of $GlnKUMP_{0\text{-}3}$ in the cytoplasm (the fractions with an asterisk below). The constraints below are a variant of *gfun*$_1$ (the total concentration is used in the denominators).

$$g_{23} = \frac{1}{n_{datapoint}} \sum_{i=1}^{n_{datapoint}} \left[\frac{[GlnKUMP_{0,cyt}]_i}{[GlnK_{total,cyt}]_i} - \left(\frac{[GlnKUMP_{0,cyt}]}{[GlnK_{total,cyt}]} \right)_i^* \right]^2 - \varepsilon_{II}^2, \cdots \quad \text{(S4.2.4-1)}$$

$$g_{24} = \frac{1}{n_{datapoint}} \sum_{i=1}^{n_{datapoint}} \left[\frac{[GlnKUMP_1]_i}{[GlnK_{total,cyt}]_i} - \left(\frac{[GlnKUMP_1]}{[GlnK_{total,cyt}]} \right)_i^* \right]^2 - \varepsilon_{II}^2, \cdots \quad \text{(S4.2.4-2)}$$

$$g_{25} = \frac{1}{n_{datapoint}} \sum_{i=1}^{n_{datapoint}} \left[\frac{[GlnKUMP_2]_i}{[GlnK_{total,cyt}]_i} - \left(\frac{[GlnKUMP_2]}{[GlnK_{total,cyt}]} \right)_i^* \right]^2 - \varepsilon_{II}^2, \cdots\cdots\cdots \text{(S4.2.4-3)}$$

$$g_{26} = \frac{1}{n_{datapoint}} \sum_{i=1}^{n_{datapoint}} \left[\frac{[GlnKUMP_3]_i}{[GlnK_{total,cyt}]_i} - \left(\frac{[GlnKUMP_3]}{[GlnK_{total,cyt}]} \right)_i^* \right]^2 - \varepsilon_{II}^2. \cdots\cdots\cdots \text{(S4.2.4-4)}$$

In the above four equations, $[GlnK_{total,cyt}] = [GlnKUMP_{0,cyt}] + \sum_{i=1}^{3} [GlnKUMP_i]$. It should be noted that $GlnKUMP_{1\text{-}3}$ cannot bind to AmtB and therefore remain in the cytoplasm. Similarly to GlnK, we semi-quantified blackness of the protein bands visible in Fig. 3B of [10] and calculated the relative abundance of GlnKAmtB complex.

$$g_{27} = \frac{1}{n_{datapoint}} \sum_{i=1}^{n_{datapoint}} \left[\frac{[GlnKAmtB]_i}{[AmtB_{total}]} - \left(\frac{[GlnKAmtB]}{[AmtB_{total}]} \right)_i^* \right]^2 - \varepsilon_{II}^2. \cdots\cdots\cdots \text{(S4.2.4-5)}$$

4.2.5 Other constraint functions

The two central amino acids glutamate and glutamine are used for anabolic purposes; i) both molecules are constituents of proteins; ii) glutamate (glutamine) also serves as amino group (amide) donor for generation of all other amino acids; iii) both molecules serve as N donors for the N-atoms present in the RNA and DNA nucleotides; iv) glutamate provides the C skeletons of arginine and proline [40, 41]. We allocated processes i-iv to four demand functions and pose constraints to the respective flux ratios as imposed by the cellular composition. The sum of the demand fluxes is determined by the specific growth rate (See Section 8.5).

$$g_{28} = gfun_2(GLU_{demf} / GLU_{demn}, 0.149, \varepsilon_{III}), \cdots\cdots\cdots \text{(S4.2.5-1)}$$

$$g_{29} = gfun_2(GLN_{demn} / GLU_{demn}, 0.254, \varepsilon_{III}), \cdots\cdots\cdots \text{(S4.2.5-2)}$$

$$g_{30} = gfun_2(GLN_{demf} / GLU_{demn}, 0.035, \varepsilon_{III}), \cdots\cdots\cdots \text{(S4.2.5-3)}$$

$$g_{31} = gfun_2(GLN_{demn} / GLU_{demf}, 1.704, \varepsilon_{III}), \cdots\cdots\cdots \text{(S4.2.5-4)}$$

$$g_{32} = gfun_2(GLN_{demf} / GLU_{demf}, 0.236, \varepsilon_{III}), \cdots\cdots\cdots \text{(S4.2.5-5)}$$

$$g_{33} = gfun_2(GLN_{demf} / GLN_{demn}, 0.138, \varepsilon_{III}). \cdots\cdots\cdots \text{(S4.2.5-6)}$$

4.2.6 Auxiliary constraint functions

To evaluate $g_{1\text{-}33}$, we have to run 9 simulations, one for each of the training data sets: wild type, ΔGDH, and ΔGOGAT for Yuan's experiments, wild type with 1000, 60, 20, 4 μM of external NH_4^+ and ΔAmtB with 40 μM of external NH_4^+ for Kim's experiments, and wild type for Radchenko's experiments. However, simulations sometimes fail because the model ordinary differential equations (ODEs) can be stiff. We use 9

auxiliary constraint functions (g_{34} up to g_{42}) to detect these failures in simulation for each training data set. If numerical integration of the model ODEs fails, $g_{34\text{-}42}$ returns a penalty of 10^{20} in each case:

$$g_i = \begin{cases} 0 & \text{if simulation success} \\ 10^{20} & \text{otherwise} \end{cases} \quad \text{for } i = 34, ..., 42 \text{.} \cdots\cdots\cdots\cdots\cdots\cdots \text{(S4.2.6-1)}$$

In addition, the model should reach steady states in case of Kim's experiments and in case of Yuan's and Radchenko's experiments before the N-perturbation is applied. Nine auxiliary constraint functions (g_{43} up to g_{51}) are used to detect the failure of the model to reach steady state, if so, $g_{43\text{-}51}$ returns 10^{10} in each case. We also used the steady state constraint (g_{52}) for wild type of Yuan's experiment *after* the 13x N-upshift in order to make sure the models reach a steady state with reasonable glutamate and glutamine levels.

$$g_i = \begin{cases} 0 & \text{if steady state reached} \\ 10^{10} & \text{otherwise} \end{cases} \quad \text{for } i = 43, ..., 52 \text{.} \cdots\cdots\cdots\cdots\cdots\cdots \text{(S4.2.6-2)}$$

The latter penalty [Eq. (S4.2.6-2)] is lower than the previous one [Eq. (S4.2.6-1)], because a simulation failure precludes a non-steady-state failure. Because the constraints $g_{34\text{-}52}$ impose large penalties compared to $g_{1\text{-}33}$, individuals with parameter sets prone to simulation failure and non-steady state run a substantial chance of being removed in the early generations of the GA search.

4.2.7 Additional constraint functions for refining the active transporter model

The following constraints ($g_{53\text{-}58}$) are based on Kim's calculation of the intracellular NH_4^+ concentrations and the rate of the AmtB-mediated ammonium transport [6]. In their calculation, Kim assumed that AmtB is an active transporter. For this reason, we did not use these constraints for the first parameter estimation in which the active and passive transporter models are compared (the section "The active transporter model is 130 times more likely than the passive transporter model" in the main text). We used these constraints only for refining the active transporter model (the section "Refining the active transporter model" in the main text).

According to Figure 3C of [6], the internal NH_4^+ is 35 μM at 60 μM external NH_4^+. The constraint is given as:

$$g_{53} = gfun_2([NH_{4int}^+]^{Kim,WT,[NH_{4ext}^+]=60\mu M}, 0.035, \varepsilon_{II}) \text{.} \cdots\cdots\cdots\cdots\cdots \text{(S4.2.7-1)}$$

The internal NH_4^+ is 26 μM at 20 μM external NH_4^+.

$$g_{54} = gfun_2([NH_{4int}^+]^{Kim,WT,[NH_{4ext}^+]=20\mu M}, 0.026, \varepsilon_{II}) \text{.} \cdots\cdots\cdots\cdots\cdots \text{(S4.2.7-2)}$$

The internal NH_4^+ is 24 μM at 4 μM external NH_4^+.

$$g_{55} = gfun_2([NH_{4int}^+]^{Kim,WT,[NH_{4ext}^+]=4\mu M}, 0.024, \varepsilon_{II}) \text{.} \cdots\cdots\cdots\cdots\cdots \text{(S4.2.7-3)}$$

The AmtB-mediated ammonium influx at 60 μM external NH_4^+ is nearly zero. Thus, we impose a penalty if the AmtB-mediated ammonium influx is more than 10 % of the ammonium assimilation flux (40 mM/min).

$$g_{56} = gfun_4(v_{amtb}^{Kim,WT,[NH_{4ext}^+]=60\mu M}, 4.0) \text{.} \cdots\cdots\cdots\cdots\cdots\cdots\cdots \text{(S4.2.7-4)}$$

According to [6], the rate of AmtB-mediated ammonium transport rate for glucose at 20 μM external NH_4^+ is 243 mM/min (this is a corrected value based on our A_{cell}, V_{cell}, and pKa).

$$g_{57} = gfun_2(v_{amtb}^{Kim,WT,[NH_{4ext}^{+}]=20\mu M}, 243, \varepsilon_{II}) \; . \cdots\cdots\cdots\cdots \text{(S4.2.7-5)}$$

Similarly, the rate of AmtB-mediated ammonium transport rate for glucose at 4 μM external NH_4^+ is 356 mM/min.

$$g_{58} = gfun_2(v_{amtb}^{Kim,WT,[NH_{4ext}^{+}]=4\mu M}, 356, \varepsilon_{II}) \; . \cdots\cdots\cdots\cdots \text{(S4.2.7-6)}$$

4.3 IS-SR-REX^{star}/JGG

Parameter estimation problems are highly nonlinear and have multiple local optima [42, 43]. For such problems, deterministic optimization methods hardly find solutions that provide sufficient fitting to training data. Population-based stochastic optimization algorithms such as genetic algorithms, evolution strategies, and scatter searches are more promising alternative approaches [43-47]. We developed the novel real-coded genetic algorithm (GA), named IS-SR-REX^{star}/JGG (Iterative Start and Stochastic Ranking-REX^{star}/JGG), in order to estimate kinetic parameters in *E. coli* ammonium transport and assimilation models. The working of this GA can be briefly explained as follows: IS-SR-REX^{star}/JGG employs REX^{star} (Real-coded Ensemble Crossover star) and JGG (Just Generation Gap) as a crossover method and a generation alternation method, respectively [48, 49]. REX^{star}/JGG find solutions much faster than UNDX (Unimodal Normal Distribution Crossover)/MGG (Minimal Generation Gap) which two of the authors of this paper employed in [37]. However, REX^{star}/JGG has a drawback: the search sometimes becomes trapped in local optima. To compensate for this weakness, we incorporate an iterative start strategy into REX^{star}/JGG: If REX^{star}/JGG does not find any solutions (i.e. parameter sets that provide $\gamma = 0$) by a predefined generation (say at generation 2,000), it discards the current search and restarts the search using a newly generated random initial population. In addition, if REX^{star}/JGG does not improve f by 1 % for 2,000 generations, it restarts the search. Also, REX^{star}/JGG (and most of GAs) has originally been designed for unconstrained optimization problem. To effectively handle constrained optimization problems, we employed the stochastic ranking method [9, 50]. In this study, the size of the initial population, the number of parents, and the number of children are 500, 300, and 500, respectively. As shown in **Figure S2**, IS-SR-REX^{star}/JGG is superior to UNDX/MGG.

Population-based stochastic optimization algorithms are computationally demanding. IS-SR-REX^{star}/JGG is no exception in this regard. To reduce the computational time, we used the development version of libRCGA [51] in which REX^{star}/JGG and UNDX/MGG are implemented in C language and paralleled by MPI. We employed CVODE [52] for numerical integration of ordinary differential equations (ODEs). We executed the IS-SR-REX^{star}/JGG on the supercomputer Shirokane3, provided by the Human Genome Center, Institute of Medical Science, the University of Tokyo. A single run for the parameter estimation took 12 hours using 21 cores of Intel Xeon E5-2670 v3 (2.3 GHz).

5 Apparent Discrepancy between Kim's and Yuan's Experimental Data

In this section, we elaborate on an apparent discrepancy (for glucose as carbon source) we observed between Kim's [6] and Yuan's [1] experimental data. We will do this step by step.

The net NH_x transfer rate v_{net} (mM/min) is given by:

$$v_{net} = v_{amtb} + v_{diff}, \quad \text{(S5-1)}$$

where v_{amtb} is the AmtB-mediated ammonium transport rate [see Eq. (S1.3-1)], and v_{diff} is the rate of unmediated NH_3 diffusion across the cytoplasmic membrane. Based on Fick's law of diffusion, v_{diff} is given by:

$$v_{diff} = \frac{P_{cm}A_{cell}}{V_{cell}}([NH_{3surf}]-[NH_{3int}]), \quad \text{(S5-2)}$$

where P_{cm} is the permeability coefficient of the cytoplasmic membrane (m/min), A_{cell} is the surface area of a cell (m^2), V_{cell} is the volume of a cell (m^3). $[NH_{3surf}]$ and $[NH_{3int}]$ are the NH_3 concentrations (mM) at the cell surface and in the cytoplasm, respectively. On the other hand, v_{net} can also be calculated on the basis of nitrogen incorporation into biomass during growth:

$$v_{net} = \mu N_0, \quad \text{(S5-3)}$$

where μ is the specific growth rate (min^{-1}), N_0 is the number of millimoles of nitrogen atoms in biomass expressed per liter of cytoplasm (mM N atoms). The intracellular concentrations of NH_4^+ and NH_3 are given by:

$$[NH_{4int}^+] = \frac{[NH_{x,int}][H_{int}^+]}{K_a + [H_{int}^+]}, \quad \text{(S5-4)}$$

$$[NH_{3int}] = \frac{[NH_{x,int}]K_a}{K_a + [H_{int}^+]}, \quad \text{(S5-5)}$$

where $[NH_{x,int}]$ is the NH_x concentration in the cytoplasm (NH_x refers to both NH_4^+ and NH_3, thus $[NH_{x,int}] = [NH_4^+{}_{int}] + [NH_{3int}]$). $[H^+{}_{int}]$ is the proton concentration in the cytoplasm. K_a is the equilibrium constant of NH_3/NH_4^+. The concentrations are expressed in mM. Combining Eqs. (S5-4) and (S5-5), the intracellular NH_3 concentration is given by:

$$[NH_{3int}] = \frac{K_a}{[H_{int}^+]}[NH_{4int}^+]. \quad \text{(S5-6)}$$

Similarly, the NH_3 concentration at the cell surface is:

$$[NH_{3surf}] = \frac{K_a}{[H_{surf}^+]}[NH_{4surf}^+]. \quad \text{(S5-7)}$$

By inserting Eqs (S5-2) – (S5-3) and (S5-6) – (S5-7) into Eq (S5-1) and solving for P_{cm}, we obtain:

$$P_{cm} = \frac{V_{cell}(\mu N_0 - v_{amtb})}{A_{cell}K_a\left(\frac{[NH_{4surf}^+]}{[H_{surf}^+]} - \frac{[NH_{4int}^+]}{[H_{int}^+]}\right)}. \quad \text{(S5-8)}$$

Let us now calculate the permeability coefficient for cytoplasmic membrane (P_{cm}) based on Kim's experimental data. We assumed that the cell surface area (A_{cell}) is 9.18 μm^2, the cell volume (V_{cell}) is 2.15 μm^3 (see Section 8.2), the concentration of nitrogen atoms per cell volume (N_0) is 3000 mM (see Section 8.1), the

pKa for ammonium (pKa) is 8.95 [53, 54], and the intracellular pH (pH_{int}) is 7.6 [55, 56]. When the NH_4^+ concentration in the medium was set to 4 μM, Kim reported that the intracellular ammonium [$NH_4^+{}_{int}$] is 24 μM, the ammonium flux by AmtB (v_{amb}) is 356 mM/min, the specific growth rate (μ) is 0.8 h^{-1}, and the pH of their medium is 7.4 (pH_{ext}; see data for glucose shown in Figure 3 of [6]). Here, we assume that the NH_x concentration at the cell surface is equal to that in the bulk medium. The same goes for the pH at the cell surface and in the bulk medium. These assumptions are considered valid since Kim cultivated cells in microfluidic chambers that were continuously supplied with liquid medium. Based on these assumptions, we obtained [$NH_4^+{}_{surf}$] = 4 μM and [$H^+{}_{surf}$] = 40 nM. By inserting the above values into Eq (S5-8), while using proper units, we obtained P_{cm} = 0.077 m/min. Thus, P_{cm} should be ~0.077 m/min in order to reproduce Kim's experimental data.

Next, let us consider Yuan's experiments. Yuan reported that in wild type *E. coli,* the glutamine concentration rapidly increases upon an ammonium upshift (see our **Figure 3a** or their Figure 4 of [1]). Since this increase occurs immediately after the ammonium upshift, it should be caused directly by the ammonium upshift, not by changes in other metabolites. The Michaelis constant of GS for NH_4^+ is 0.1 mM [57]. Thus, to explain the increase in glutamine production upon the ammonium upshift, the intracellular NH_4^+ concentration before the upshift should be much less than 0.1 mM. Solving Eq (S5-8) for the intracellular NH_4^+ concentration, we obtain:

$$[NH_{4int}^+] = [H_{int}^+]\left(\frac{[NH_{4surf}^+]}{[H_{surf}^+]} - \frac{V_{cell}(\mu N_0 - v_{amtb})}{A_{cell}K_a P_{cm}}\right). \quad \text{(S5-9)}$$

Yuan reported that the NH_4^+ concentration at the surface of the filter was 0.75 mM, the specific growth rate was 0.19 h^{-1} before the ammonium upshift, and the medium pH was 7.0 throughout the experiment. We again assume that the NH_x concentrations at the cell surface and at the surface of the filter are equal, and that the same is valid for the pH (but see the next paragraph). Thus, [$NH_4^+{}_{surf}$] = 0.75 mM and [$H^+{}_{surf}$] = 100 nM. Since Yuan reported that *E. coli* is N-limited in this situation, AmtB was probably operative (v_{amtb} > 0 mM/min). Using those values and Eq. (S5-9), we obtain [$NH_4^+{}_{int}$] > 0.19 mM, which is twice the K_m of GS for NH_4^+. Consequently, the possible increase in glutamine production rate upon the ammonium upshift is predicted to be at most ~1.5-fold, which cannot explain the observed sharp glutamine increase upon the ammonium upshift (which is shown in our **Figure 3a** or Yuan's Figure 4 of [1]).

In summary, there is an apparent inconsistency between Kim's and Yuan's experiments: Based on Kim's experiments, we obtained P_{cm} = 0.077 m/min, but this is not congruent with Yuan's experiments. Different values for P_{cm} have been reported in the literature: 0.0012 m/min [58], 0.078 m/min [59], 0.12 m/min [60]. Even if the lowest of these values is used, the sharp increase in glutamine upon the ammonium upshift observed in Yuan's experiments still cannot be explained: [$NH_4^+{}_{int}$] > 0.14 mM and the increase in glutamine production rate can only be maximally 1.7-fold. The sharp increase in glutamine can be explained only if [$NH_4^+{}_{surf}$] would be (much) smaller than 0.75 mM.

6 Discussion on the Mass Transfer Capacity Coefficient (k_{db})

As we described in Section 1.5, we modeled the ammonium/ammonia (NH_x) transfer from the bulk medium to the cell surface through the diffusion barrier in order to explain Yuan's experimental data:

$$v_{db} = k_{db}\left([NH_{x,ext}]-[NH_{x,surf}]\right), \quad \text{(S6-1)}$$

where k_{db} is the mass transfer capacity coefficient for NH_x diffusion through the diffusion barrier (min^{-1}), and the estimated value is 13.9 min^{-1}. $[NH_{x,ext}]$ and $[NH_{x,surf}]$ are the NH_x concentrations (mM) in the bulk medium and at the cell surface, respectively. In this section, we first show that Yuan implicitly assumed the presence of a diffusion barrier in their model. Then, we discuss the validity of the estimated value of k_{db}.

6.1 The Yuan model implicitly includes a diffusion boundary for NH_x

Yuan did not explicitly model the ammonium/ammonia diffusion barrier or the AmtB-mediated ammonium transport. In the Yuan model, the rate of NH_3 diffusion from the medium to cytoplasm is given by:

$$v_{diff} = k_{diff}([NH_{3ext}]-[NH_{3int}]), \quad \text{(S6.1-1)}$$

where $[NH_{3ext}]$ and $[NH_{3int}]$ are NH_3 concentrations in medium and cytoplasm, respectively. The term k_{diff} is the "ammonia membrane diffusion constant" (See Supp. Table 3 of [1]). Here, we assume "membrane" means cytoplasmic membrane. Thus, in standard terminology, k_{diff} is the mass transfer capacity coefficient for NH_3 in cytoplasmic membrane. Yuan estimated k_{diff} to amount to 24.6 min^{-1} to fit their model to Fig. 4 of [1]. In general, the mass transfer capacity coefficient consists of three parameters:

$$k_{diff} = \frac{P_{cm}A_{cell}}{V_{cell}}, \quad \text{(S6.1-2)}$$

where, P_{cm} is the permeability coefficient for cytoplasmic membrane, and A_{cell} and V_{cell} are the area and volume of a single cell. If we use $A_{cell} = 9.18 \times 10^{-12}$ m^2 and $V_{cell} = 2.15 \times 10^{-18}$ m^3, we obtain $P_{cm} = 5.76 \times 10^{-6}$ m/min, which is too low to be realistic (our P_{cm} is ~0.077 m/min, as derived from [6]). Therefore, k_{diff} cannot be considered a mass transfer capacity coefficient for NH_3 in cytoplasmic membrane.

What if we interpret "NH_3" as NH_x in Eq. (S6.1-1)? Yuan did not distinguish NH_3 and NH_4^+, and it is unclear what "NH_3" in Eq. (S6.1-1) actually means, NH_3 or NH_x. Here, we interpret that "NH_3" in Eq. (S6.1-1) means NH_x. Then, Eq. (S6.1-1) is rewritten as

$$v_{diff} = k_{diff}([NH_{x,ext}]-[NH_{x,int}]). \quad \text{(S6.1-3)}$$

Since NH_3 can diffuse through cytoplasmic membrane, but NH_4^+ cannot, we can interpret that k_{diff} in Eq. (S6.1-3) is a composite of the mass transfer capacity coefficient for NH_3 in the cytoplasmic membrane and a factor converting NH_x concentration to NH_3 concentration:

$$k_{diff} = k'_{diff} \cdot factor. \quad \text{(S6.1-4)}$$

Using pKa = 8.95 and pH = 7.0 [1], we obtain factor = 0.011 (pH differs in medium and cytoplasm, but it does not matter here because $[NH_{x,int}] << [NH_{x,ext}]$, i.e. the cells are N-limited). Therefore, k'_{diff} is larger than k_{diff} by

two orders of magnitude: k'_{diff}= 2.22 x 10^3 min^{-1}. Inserting k'_{diff} into k_{diff} in Eq. (S6.1-2), we obtain P_{cm} = 5.20 x 10^{-4} m/min, which is again too low to be realistic.

Then, what actually does Eq. (S6.1-1) mean in the Yuan model? Yuan used a very small value for k_{diff}, indicating that they (planned or unplanned) assumed something hinders NH_x diffusion, i.e. a diffusion barrier. Below, we show that Eq. (S6.1-1) virtually represents NH_x diffusion from medium to cytoplasm through the NH_x diffusion barrier. Here, we assume that "NH_3" in Eq. (S6.1-1) means NH_x, thus, we use Eq. (S6.1-3) again, but we interpret it to represent NH_x diffusion through the diffusion barrier instead of NH_x diffusion through the cytoplasmic membrane. Please note that Eq. (S6.1-3) resembles our rate equation of NH_x diffusion through the diffusion barrier [Eq. (S6-1)]. Here, $[NH_{x,ext}]$ = 0.75 mM for the wild type before N-upshift [1]. The Yuan model predicted $[NH_{x,int}] << [NH_{x,ext}]$. v_{diff} was predicted to be ~19 mM/min to support the specific growth rate of 0.19 h^{-1}, where Yuan assumed N_0 = ~6 mol N/L cyt. From these values of $[NH_{x,ext}]$, $[NH_{x,int}]$, and v_{diff}, Yuan estimated k_{diff} to be 24.6 min^{-1} [1]. In Eq. (S6-1), i.e. our rate equation for NH_x diffusion through the diffusion barrier, $[NH_{x,ext}]$ = 0.75 mM [1], $[NH_{x,surf}] << [NH_{x,ext}]$ (our model prediction), v_{db} = ~9.5 mM/min (N_0 = ~3 mol N/L cyt), we arrived at k_{db} =13.9 min^{-1}. The two-fold difference between k_{diff} and k_{db} simply comes from the difference in N_0 values assumed by Yuan and us. Therefore, Eq. (S6.1-3) is virtually equivalent to our rate equation of NH_x diffusion through the diffusion barrier [Eq. (S6-1)]. In other words, the Yuan model includes the NH_x diffusion barrier.

6.2 Thickness of the diffusion barrier

In our parameter estimation, we categorized k_{db} as a Class III parameter and estimated its value [see Eq. (S6-1)]. The estimated value is 13.9 min^{-1}. In general, mass transfer capacity coefficient consists of the permeability coefficient, the area through which molecules are being transferred, and the volume of the compartment that receives the molecules:

$$k_{db} = \frac{P_{db} A_{surf}}{V_{surf}}, \quad \text{(S6.2-1)}$$

where P_{db} is the permeability coefficient for NH_x in the diffusion barrier, and A_{surf} and V_{surf} are the area and volume of the surface compartment, respectively. The permeability coefficient is given by the diffusion coefficient divided by the thickness of the diffusion barrier:

$$P_{db} = D_{db} / L_{db}. \quad \text{(S6.2-2)}$$

Inserting Eqs. (S6.2-1) – (S6.2-2) into Eq. (S6-1), we obtain the following equation:

$$v_{db} = \frac{D_{db} A_{surf}}{L_{db} V_{surf}} \left([NH_{x,ext}] - [NH_{x,surf}] \right). \quad \text{(S6.2-3)}$$

The refined active transporter predicted $[NH_{x,surf}]$ < 1 μM before the N-upshift in Yuan's experiments. This prediction is consistent with Kim's experimental data: wild type cells grow at the maximum specific growth rate under extracellular NH_x concentrations as low as 4 μM (the extracellular and surface NH_x concentrations are equal in Kim's experiments). Thus, for *E. coli* to show a reduced specific growth rate, $[NH_{xsurf}]$ needs to

be less than 4 μM. In Yuan's experiments, the $[NH_{x,ext}]$ = 0.75 mM before the N-upshift. Then by assuming $[NH_{x,surf}] << [NH_{x,ext}]$, Eq. (S6.2-3) becomes:

$$v_{db} \approx \frac{D_{db} A_{surf} [NH_{x,ext}]}{L_{db} V_{surf}} . \quad \text{(S6.2-4)}$$

To support the specific growth rate of 0.19 h^{-1} before the N-upshift, NH_x consumption rate must be around 9.5 mM/min, thus, $v_{db} \approx$ 9.5 mM/min. We assumed that the diffusion coefficient for NH_x in agarose medium and filter is equal to that in water. Since most NH_x molecules exist in the NH_4^+ form around pH = 7.0, the diffusion coefficient of NH_4^+ in water can be used as D_{db}: $D_{db} = 1.12 \times 10^{-7}$ m^2 min^{-1} [5]. A_{surf} and V_{surf} are the area and volume of the surface compartment. Since the surface compartment is a conceptual compartment, the determination of A_{surf} and V_{surf} is somewhat arbitrary. Here, we assume that the surface compartment is a space at distance of up to 10 μm from the cell surface; then, $A_{surf} = 1.50 \times 10^{-9}$ m^2 and $V_{surf} = 5.47 \times 10^{-15}$ m^3. Using the above values for $[NH_{x,ext}]$, v_{db}, D_{db}, A_{surf}, V_{surf}, the thickness of the diffusion barrier can be calculated. Transforming Eq. (S6.2-4), we obtain:

$$L_{db} \approx \frac{D_{db} A_{surf} [NH_{x,ext}]}{v_{db} V_{surf}} . \quad \text{(S6.2-5)}$$

Inserting the above parameters into Eq. (S6.2-5), we obtain L_{db} = 2.4 mm. Considering the fact that the thickness of the agar is 2.5 mm (see **Figure S3**), the 2.4 mm thickness is relatively high (This qualitatively holds true for the Yuan model as well. Since they assumed N_0 = ~6 mol N/L cyt, which is twice our value, L_{db} = ~ 1.2 mm for the Yuan model).

Because of limited information about the experiment, we cannot pinpoint what causes the thick diffusion barrier. Here, we suggest five possible reasons. (i) D_{db} might be smaller than we assumed because the diffusion barrier is a composite of agarose medium and filter in Yuan's experiments. In fact, based on Fig. 1b of [2], we estimated that the diffusion coefficient in the filter is around one fifth of that in water (we assumed the filter to be 100 μm thick). (ii) A_{surf} might be smaller than we assumed. We assumed above that NH_x diffuses to a rod-shaped cell from all directions. However, in Yuan's experiments, NH_x probably only diffuses in a vertical direction from the medium to the cells. (iii) The cells formed multi-layered colonies on the plate, which decreased the effective cell surface area, thus, A_{surf}. Indeed, our calculation suggests cells may have formed multi-layered colonies. Yuan used 5 ml of culture with ~0.085 OD_{650} to inoculate cells on the filter with 82 mm diameter. Thus, there are ~5.66×10^8 cells at the start of the experiment. Assuming a single cell has a 'flat' area of 2.7 $μm^2$, then 15.3 cm^2 of the filter is occupied by the cells in total. As the cells grow for 3 generations on the filter, 122 cm^2 would be occupied at 180 min. Considering the fact the filter has 53 cm^2 surface area, it is likely that the cells formed multi-layered colonies. (iv) Another possible factor to reduce A_{surf} is air bubbles. If tiny air bubbles were present, they would hamper NH_x transfer, and A_{surf} would then decrease. (v) $[NH_{x,ext}]$ might be smaller than Yuan reported (0.75 mM). Yuan claimed that they measured the ammonium concentration at the surface of the medium. However, it was not possible to obtain the necessary details of their measurement. Therefore, we simulated NH_x diffusion and consumption by wild type cells in Yuan's filter

culture. As shown in **Figure S3**, the simulation with reasonable physical and biochemical parameters suggests that the NH_x concentration at the surface of the medium could have been lower than 0.75 mM just before the N-upshift was administered at 180 min. Our simulation predicts that, at 180 min, the NH_x concentration at the top of the medium is 0.29 mM, and that at the top of filter is 7.4 μM.

7 Derivation of the Rate Equation of AmtB-mediated Ammonium Transport

To test the active transporter and the passive transporter hypotheses of AmtB, we derived the rate equations based on these hypotheses. Although the transport hypotheses are different, the derived rate equations can be converted into the same equation [Eq. (S1.3-1)] with different theoretical accumulation factors. In this section, we elaborate on the derivation of the rate equations for AmtB-mediated ammonium transport and how these can be transformed into Eq. (S1.3-1).

7.1 Active transport of NH_3

For active transport of NH_3, we start off with the reaction scheme shown in **Figure S1a**. Please note that NH_4^+ is transported in **Figure S1a**, but it is also valid if NH_3 and H^+ are separately transported, provided that their transport is coupled. As shown in **Figure S1b**, AmtB-NH_4^+ complex has two states: One open towards the outside and the other open towards the inside. During transport, first, external NH_4^+ binds to AmtB. Then, the AmtB-NH_4^+ complex switches to the inwardly open conformation. Finally, AmtB releases NH_4^+ into cytoplasm. The carrier AmtB is distributed over three states, i.e. empty carrier (E), carriers with NH_4^+ bound at the binding site which is either open towards the outside (EN_{4ext}) or the inside (EN_{4int}). We assume that NH_4^+ binding to the carrier is at equilibrium and that the conformational change is the rate-limiting step. The total amount of carrier protein is given by:

$$[E_{total}] = [E] + [EN_{4ext}] + [EN_{4int}]. \quad \text{(S7.1-1)}$$

The equilibrium reactions are:

$$K_{ext}[EN_{4ext}] = [E][N_{4ext}], \quad \text{(S7.1-2)}$$

$$K_{int}[EN_{4int}] = [E][N_{4int}], \quad \text{(S7.1-3)}$$

where N_{4ext} and N_{4int} indicate the external and the internal NH_4^+, respectively. The rate of NH_4^+ transport is:

$$v_{amtb} = k_f[EN_{4ext}] - k_r[EN_{4int}]. \quad \text{(S7.1-4)}$$

Using Eqs. (S7.1-1) – (S7.1-3), Eq. (S7.1-4) can be transformed to a function of $[E_{total}]$, $[N_{4ext}]$ and $[N_{4int}]$:

$$v_{amtb} = \frac{[E_{total}]\left(\dfrac{k_f[N_{4ext}]}{K_{ext}} - \dfrac{k_r[N_{4int}]}{K_{int}}\right)}{1 + \dfrac{[N_{4ext}]}{K_{ext}} + \dfrac{[N_{4int}]}{K_{int}}}. \quad \text{(S7.1-5)}$$

At carrier equilibrium ($v_{amtb} = 0$), the ratio of the internal to the external NH_4^+ is:

$$\left(\frac{[N_{4int}]}{[N_{4ext}]}\right)_{eq} = \frac{k_f K_{int}}{k_r K_{ext}}. \quad \text{(S7.1-6)}$$

Thus, the ratio is determined by k_f, k_r, K_{ext}, and K_{int}. These parameters are not quite independent however. At

carrier equilibrium, the electrochemical potential difference across the cytoplasmic membrane for NH_4^+ is given by:

$$0 = \Delta\mu_{NH_4^+} = RT \ln\left(\frac{[N_{4int}]}{[N_{4ext}]}\right)_{eq} + F \cdot \Delta\psi \text{ ,} \qquad \text{(S7.1-7)}$$

where $\Delta\psi$ is the transmembrane electrical potential (usually in the order of 150 mV for *E. coli*), F is the Faraday constant, R is the gas constant, and T is the absolute temperature. According to Eq. (S7.1-7), the theoretical accumulation factor of NH_4^+ (φ) is given by:

$$\varphi = \left(\frac{[N_{4int}]}{[N_{4ext}]}\right)_{eq} = \exp\left(\frac{-F \cdot \Delta\psi}{RT}\right). \qquad \text{(S7.1-8)}$$

Assuming $\Delta\psi$ = -0.15 V and T = 310 K, φ = 275. According to Eqs. (S7.1-6) and (S7.1-8), k_f, k_r, K_{ext}, and K_{int} must satisfy the following thermodynamic constraint:

$$\frac{k_f K_{int}}{k_r K_{ext}} = \varphi \text{ .} \qquad \text{(S7.1-9)}$$

Inserting Eq. (S7.1-9) into Eq. (S7.1-5), we obtain:

$$v_{amtb} = \frac{k_f [E_{total}]\left([N_{4ext}] - \frac{[N_{4int}]}{\varphi}\right)}{K_{ext}\left(1 + \frac{[N_{4int}]}{K_{int}}\right) + [N_{4ext}]}. \qquad \text{(S7.1-10)}$$

We assume the carrier is kinetically symmetric, i.e. the probabilities of the outwardly open and the inwardly open states are equal ($k_f = k_r$). Thus, K_{int}, = φ K_{ext} implying that the transmembrane electric potential affects AmtB-NH_4^+ binding but not conformational change of the carrier. We use K_{ext} = 5 μM (see Section 8.4) and φ = 275, and thus K_{int} = 1.38 mM. With these values of K_{ext} and K_{int}, Eq. (S7.1-10) can be approximated by the following equation:

$$v_{amtb} = \frac{k_f [E_{total}]\left([N_{4ext}] - \frac{[N_{4int}]}{\varphi}\right)}{K_{ext} + [N_{4ext}]}, \qquad \text{(S7.1-11)}$$

Finally, replacing the variables in Eq (S7.1-11) with the model variables and parameters, the following rate equation is obtained:

$$v_{amtb} = \frac{k_{catamtb} [AmtB_{GlnKfree}]\left([NH_{4surf}^+] - \frac{[NH_{4int}^+]}{\varphi}\right)}{K_{amtbnh} + [NH_{4surf}^+]}, \qquad \text{(S7.1-12)}$$

where $AmtB_{GlnKfree}$ depicts GlnK-unbound AmtB, i.e. the operative form of AmtB.

7.2 Facilitated passive transport of NH_3

According to the facilitated passive transport mechanism proposed by Khademi et al. [61], NH_4^+ binds to the periplasmic cage of AmtB, then becomes deprotonated and moves to the middle membrane channel, whereafter

NH_3 is released into the cytoplasm (**Figure S1c**). Based on this mechanism, we derive a rate equation for the facilitated passive transport of NH_3. As shown in **Figure S1d**, AmtB is distributed over three states: the empty carrier (E), the carrier with NH_4^+ at the periplasmic cage (EN_{4ext}), and the carrier with NH_3 at the mid-membrane path (EN_{3mm}). We assume NH_4^+ binding to the periplasmic cage and deprotonation to be at equilibrium, and NH_3 transport from the mid-membrane to the cytoplasm to be the rate-limiting step. The total amount of carrier protein is given by:

$$[E_{total}] = [E] + [EN_{4ext}] + [EN_{3mm}]. \quad \text{(S7.2-1)}$$

The equilibrium reactions are:

$$K_{ext}[EN_{4ext}] = [E][N_{4ext}], \quad \text{(S7.2-2)}$$

$$K_{mm}[EN_{4ext}] = [EN_{3mm}][H^+_{ext}], \quad \text{(S7.2-3)}$$

where N_{4ext} and H^+_{ext} are the external NH_4^+ and H^+, respectively. The rate of NH_3 transport is:

$$v_{amtb} = k_f[EN_{3mm}] - k_r[E][N_{3int}]. \quad \text{(S7.2-4)}$$

N_{3int} represents the internal NH_3. Using Eqs. (S7.2-1) – (S7.2-3), Eq. (S7.2-4) can be transformed to a function of $[E_{total}]$, $[N_{3ext}]$ and $[N_{3int}]$:

$$v_{amtb} = \frac{[E_{total}]\left(\dfrac{k_f K_{mm}}{K_{ext}K_a}[N_{3ext}] - k_r[N_{3int}]\right)}{1 + \dfrac{[N_{3ext}]}{K_{ext}K_a}\left([H^+_{ext}] + K_{mm}\right)}, \quad \text{(S7.2-5)}$$

where N_{3ext} and N_{3int} are the external and internal NH_3, respectively. K_a is the equilibrium constant for ammonium. In the derivation of Eq. (S7.2-5), $[N_{4ext}]/[N_{3ext}] = [H^+_{ext}]/K_a$ was used. Since NH_3 transport is assumed to be passive (i.e. no free energy is consumed, and thus the NH_3 concentration gradient is the sole driving force of NH_3 transport), the ratio of the internal to the external NH_3 at carrier equilibrium ($v_{amtb} = 0$) is:

$$\left(\frac{[N_{3int}]}{[N_{3ext}]}\right)_{eq} = \frac{k_f K_{mm}}{k_r K_{ext} K_a} = 1. \quad \text{(S7.2-6)}$$

k_f, k_r, K_{ext}, K_{mm}, and K_a must satisfy this relationship. Next, we introduce three composite parameters:

$$k'_f = \frac{k_f K_{mm}}{K_{mm} + [H^+_{ext}]}, \quad \text{(S7.2-7)}$$

$$K_m = \frac{K_{ext}[H^+_{ext}]}{K_{mm} + [H^+_{ext}]}, \quad \text{(S7.2-8)}$$

$$\varphi = \frac{[H^+_{int}]}{[H^+_{ext}]} = 10^{pH_{ext} - pH_{int}}, \quad \text{(S7.2-9)}$$

where H^+_{int} is the internal H^+. Using Eqs, (S7.2-6) – (S7.2-9), Eq. (S7.2-5) can be rewritten as

$$v_{amtb} = \frac{k'_f[E_{total}]\left([N_{4ext}] - \dfrac{[N_{4int}]}{\varphi}\right)}{K_m + [N_{4ext}]}, \quad \text{(S7.2-10)}$$

where N_{4ext} and N_{4int} are the external and internal NH_4, respectively. Finally, replacing the variables in Eq (S7.2-10) with the model variables and parameters, the following rate equation is obtained:

$$v_{amtb} = \frac{k_{catamtb}[AmtB_{GlnKfree}]\left([NH_{4surf}^{+}] - \frac{[NH_{4int}^{+}]}{\varphi}\right)}{K_{amtbnh} + [NH_{4surf}^{+}]}, \quad \text{(S7.2-11)}$$

where $AmtB_{GlnKfree}$ depicts GlnK-unbound AmtB, i.e. the operative form of AmtB.

8 Derivation of Key Parameter Values

8.1 Number of nitrogen atoms per liter cell volume (N_0)

Using the nitrogen content of biomass for ammonium-limited cells of 0.108 gN/gDW [62] and 2.5 μL/mgDW [63], we obtain $N_0 \approx 3$ M:

$$N_0 = \frac{0.108\left[\frac{g_N}{g_{DW}}\right]}{0.0025\left[\frac{L}{g_{DW}}\right] \times 14\left[\frac{g_N}{mol}\right]} \approx 3\left[\frac{mol}{L}\right], \quad \text{(S8.1-1)}$$

This value is the same as the value Kim used [6], but their calculation is based on different experimental data (see Supp. Table 3 of [6]).

8.2 Cell volume (V_{cell}) and surface area (A_{cell})

We use $V_{cell} = 2.15$ μm^3 and $A_{cell} = 9.18$ μm^2. Radzikowski et al. estimated that the cell volume is 2.15 μm^3 for cells growing on glucose in the exponential growth phase [7]. It should be noted that the same research group had reported a larger cell volume (3.2 μm^3) in [8], but recently asserted that this was an overestimation [64]. Since cell surface area is not provided in [7], we estimated it as follows. (i) $V_{cell} = 2.15$ μm^3 [7]. (ii) The cell length was 2.5-fold larger than the cell width [7]. (iii) We assumed the cells to have the shape of a cylinder capped by two half-spheres [8]:

$$V_{cell} = \frac{\pi w^2}{4}\left(l - \frac{w}{3}\right), \quad \text{(S8.2-1)}$$

$$A_{cell} = \pi w l, \quad \text{(S8.2-2)}$$

where l and w are the cell length and width, respectively. Based on (i) – (iii), we obtained $A_{cell} = 9.18$ μm^2 (l = 2.70 μm and w = 1.08 μm). Are $V_{cell} = 2.15$ μm^3 and $A_{cell} = 9.18$ μm^2 reasonable? These V_{cell} and A_{cell} values yield $V_{cell}/A_{cell} = 0.23$ μm which is within the range Kim reported [6]. Using cell volume per dry weight of 2.5 μL/mgDW (= 400 gDW/L) [63] and $V_{cell} = 2.15$ μm^3, we obtain 860 fgDW/cell which is within the physiological range [65]. Protein constitutes 50 – 60 % of cell dry weight, so we get 430 – 516 fg protein/cell, which is close to the value reported in [64] (In [64], 280 fg protein/cell was obtained using a cell volume of 3.2 μm^3. If we replace this with 2.15 μm^3, the protein weight will be 417 fg protein/cell).

8.3 V_{max} of GS, GOGAT, and GDH (V_{gs}, V_{gog}, and V_{gdh})

Most data available in the literature for the V_{max} of GS are based on the γ–glutamyl transferase assay, which measures the reverse reaction of GS with hydroxylamine and glutamine as substrates. However, we prefer using data obtained for the biosynthetic GS assay that employs ammonium and glutamate as natural substrates. Also, we prefer using data obtained for ammonium-limited chemostat cultures, where GS will be not (or hardly) adenylylated. Kumar and Shimizu observed a V_{max} value for GS of ~ 80 mM/min for cells growing in an ammonium-limited chemostat at 0.2 h^{-1} [33]. A higher V_{gs} was obtained in Bruggeman et al. [4] though and we will use this value, as well as their values for V_{gog} and V_{gdh}: V_{gs} = 600 mM/min, V_{gog} = 85 mM/min, and V_{gdh} = 360 mM/min. However, these V_{max}'s were measured under enzyme-specific in vitro conditions. We corrected these values considering the fact that V_{max} in vitro may be different from that in vivo [66]. According to Figure 1 of [66], V_{gs} and V_{gog} as measured under in vivo-like conditions are reduced to 45 % and 85 % of their in vitro values, respectively. Thus, we used V_{gs} = 270 mM/min and V_{gog} = 72 mM/min as reference values. We did not correct V_{gdh} because the in vivo-in vitro difference in V_{gdh} stems primarily from K^+ (see Fig. 5 of [66]) and sufficient K^+ (>20 mM) was present in the assay medium used in [14]. Since GS itself also functions as a substrate in the adenylylation reaction catalyzed by ATase, we separated the V_{gs} of 270 mM/min into k_{catgs} and GS_{total}.

Unfortunately, there is no estimate of the total GS concentration for ammonium-limited cells but only for cells grown with glutamine plus excess ammonium and for cells grown with glutamine as the single N-source (with glucose). Van Heeswijk et al. measured 1.8 μM GS dodecamer for the former condition and 11 – 14 μM for the latter condition [67, 68]. Thus, the fold change is 6 – 8. However, it might be different for ammonium-limited cells. The latter fold change can be indirectly calculated using data from Senior [69]. Senior did not measure GS levels, but measured V_{max}'s of GS (γ–glutamyl transferase assay; with Mn^{2+}) in cells growing in chemostats under ammonium-limited and ammonium excess conditions (with glucose). The fold change was 4.4 or 3.5, depending on the range of values used to calculate average values for the (more or less constant) V_{max}'s over the μ range from 0.05 - 0.6 h^{-1} (Fig. 1 of [69]) and 0.05 - 0.6 or 0.05 - 0.9 h^{-1} (as calculated from the inverse doubling time on the X-axis of Fig. 6 of [69]), respectively. On the assumption that this fold change also applies to GS levels, a GS dodecamer concentration of 8 or 6 μM (average 7 μM) for ammonium-limited cells is obtained by multiplying 1.8 with 4.4 or 3.5. This moderate fold change is consistent with Yuan [1] and Kim [6]. According to Fig. 2C of [1], the blackness of the GS band of Western blot moderately increased upon transition from ammonium excess to limitation (with glucose). According to Supp. Table 8 of [6], mCherry (*glnA*) promoter activity increased by 3-fold upon transition from ammonium excess to limitation (with glucose). In conclusion, we took GS_{total} = 7 μM as the reference value, and thus k_{catgs} = 38571 min^{-1} to obtain V_{gs} of 270 mM/min.

8.4 AmtB and GlnK-related parameters ($k_{catamtb}$, K_{amtbnh}, $AmtB_{total}$, $GlnK_{total}$, and $K_{glnkog1-3}$)

The parameter $k_{catamtb}$ represents the number of NH_x molecules transported by an AmtB trimer per minute. We use $k_{catamtb} = 4.86 \times 10^5$ min^{-1} as the reference value in parameter estimation. According to [61], an AmtB single channel conductance is ~27000 s^{-1} (see Table 3 of [70]). However, as pointed out by Javelle et al. [70], the

proteo-liposome experiments done in [61] were not reproducible. Javelle et al. [70] speculated that the single channel conductance is 10 – 100-fold lower than the value reported in [61]. Thus, we took 2700 s^{-1} (per monomer), i.e. $k_{catamtb}$ = 4.86 x 10^5 min^{-1} (per trimer). This value is within the range estimated by Zheng et al. [71]: 10^1 – 10^4 s^{-1} per monomer. Recently, Wacker et al. reported that the conductance of *Archaeoglobus fulgidus* Amt is 30 – 300 s^{-1} per trimer [72]. Their experiment was conducted at 20 – 23 °C, but *A. fulgidus* is a thermophile and grows optimally at 83 °C. Assuming Q_{10} = 2 (the transport rate doubles as the temperature increases by 10 °C), we estimated the conductance rate under its physiological condition to be 1920 – 23600 s^{-1} per trimer, i.e. 640 – 7867 s^{-1} per monomer. The value we used for single channel conductance (2700 s^{-1} per monomer) is within this range. The K_m of AmtB for ammonium (K_{amtbnh}) is probably several μM [20, 70, 73]. We took 5 μM for K_{amtbnh}.

Data on cellular quantities of AmtB and GlnK are scarce. According to van Heeswijk et al. [68], GlnK is 1.7-fold higher than GlnB under N-limited condition. In our study, $GlnB_{total}$ is 0.65 μM and $GlnK_{total}$ to 2 μM, so the difference is 3-fold. Moreover, for GlnK to bind AmtB in 1:1 ratio and effectively block ammonium transport, AmtB should be somewhat lower than GlnK. Also, Blauwkamp and Ninfa found that coexpression of AmtB and GlnK at proportional levels are required [74]. It should be noted though that Radchenko et al. found a stoichiometric ratio of 8 for GlnK to AmtB with AmtB present at 1 μM (1353 trimers per cell) [36]. At any rate, it seems likely that the AmtB level is less than the GlnK level. Therefore, we took 1.5 μM for $AmtB_{total}$. Assuming V_{cell} = 2.15 μm^3 and A_{cell} = 9.18 μm^2, we obtain 634 AmtB monomers/μm^2, which is within the range assumed in [71].

Since GlnK is a paralogue of GlnB, we assumed that GlnK-related kinetic parameters are similar to the corresponding counterparts of GlnB. However, we make an exception for the parameters $K_{glnbog1-3}$ (dissociation constants for GlnK and 2-oxoglutarate). Atkinson and Ninfa determined a $K_{activation}$ of 2-oxoglutarate for uridylylation of GlnK (Fig. 7 of [75]), which would be ~ 10 μM. In analogy with GlnB, the $K_{glnkog1-3}$ might have similar values. This was however measured in a reconstituted system with purified UTase (10 nM) and GlnK (15 μM). Moreover, 2-oxoglutarate concentration was measured to be ~10 mM under N-limited condition and ~1 mM under N-rich condition [1]. Thus, if $K_{glnkog1-3}$ were ~10 μM, then 2-oxoglutarate would be bound to GlnK irrespective of N-status, and consequently, GlnK could not block AmtB-mediated ammonium transport. Considering that the physiological range of 2-oxoglutarate is 1 – 10 mM and GlnK-AmtB binding is responsive to changes in the 2-oxoglutarate level, we inferred that the values of $K_{glnkog1-3}$ should be in the mM range. We have chosen 5 mM for the reference values of $K_{glnkog1-3}$.

8.5 GLU_{demn}, GLU_{demf}, GLN_{demn}, and GLN_{demf}

As shown in Table S2, the balance equations for glutamate (GLU) and glutamine (GLN) are:

$$\begin{aligned} \frac{d[GLU]}{dt} &= v_{gdh} + 2v_{gog} - v_{gs} - v_{gludemn} - v_{gludemf} + v_{glndemn} \\ \frac{d[GLN]}{dt} &= -v_{gog} + v_{gs} - v_{glndemn} - v_{glndemf} \end{aligned} , \quad \text{(S8.5-1)}$$

where the unit is mM/min. As shown in Table S1, the consumption rates are:

$$\begin{aligned} v_{gludemn} &= \mu \cdot GLU_{demn} \\ v_{gludemf} &= \mu \cdot GLU_{demf} \\ v_{glndemn} &= \mu \cdot GLN_{demn} \\ v_{glndemf} &= \mu \cdot GLN_{demf} \end{aligned} \quad , \cdots\cdots \text{(S8.5-2)}$$

where μ is the specific growth rate (min^{-1}), and GLU_{demn}, GLU_{demf}, GLN_{demn}, and GLN_{demf} are biosynthetic requirements (mM). Inserting Eqs. (S8.5-2) into Eqs. (S8.5-1) and assuming steady state, we obtain:

$$\begin{aligned} 0 &= \frac{v_{gdh}}{\mu} + \frac{2v_{gog}}{\mu} - \frac{v_{gs}}{\mu} - GLU_{demn} - GLU_{demf} + GLN_{demn} \\ 0 &= -\frac{v_{gog}}{\mu} + \frac{v_{gs}}{\mu} - GLN_{demn} - GLN_{demf} \end{aligned} \quad . \cdots\cdots \text{(S8.5-3)}$$

According to Table 1 of Reitzer [40], biosynthetic requirements of glutamate and glutamine as amino acids are 1060 and 250 μmol/gDW, respectively. Using these values and 2.5 x 10^{-3} L/gDW [63], we obtain GLU_{demf} = 424 mM and GLN_{demf} = 100 mM. Biosynthetic requirement of glutamate for amino transfer is 7108 μmol/gDW, i.e. GLU_{demn} = 2843 mM. Biosynthetic requirement of glutamine for amino transfer is 10226 μmol/gDW, i.e. (v_{gog}/μ) + GLN_{demn} = 4090 mM. According to the foot note of Table 1 of [40], Reitzer assumed that glutamate is synthesized via GS-GOGAT cycle, i.e. v_{gdh} = 0. Taken together, we obtain a system of three algebraic equations:

$$\begin{aligned} 0 &= \frac{2v_{gog}}{\mu} - \frac{v_{gs}}{\mu} - 2843 - 424 + GLN_{demn} \\ 0 &= -\frac{v_{gog}}{\mu} + \frac{v_{gs}}{\mu} - GLN_{demn} - 100 \\ \frac{v_{gog}}{\mu} &+ GLN_{demn} = 4090 \end{aligned} \quad , \cdots\cdots \text{(S8.5-4)}$$

where the unit is mM for all the three equations. By solving this system, we obtain GLN_{demn} = 723 mM with v_{gs}/μ = 4190 mM and v_{gog}/μ = 3367 mM.

It should be noted that Table 1 of [40] is for *E. coli* grown under N-rich condition. Indeed, the sum of nitrogen content (the sum of the far-right column of Table 1 of [40]) is 10283 μmol/gDW, which amounts to 14.4 % (gN/gDCW). In contrast, we use the percentage of N content for N-limited cells, i.e. 10.8 % (gN/gDCW) [62]. Thus, we correct the above obtained four biosynthetic requirements by 75 % (10.8/14.4). Consequently, we use GLU_{demn} = 2132 mM, GLU_{demf} = 318 mM, GLN_{demn} = 542 mM, and GLN_{demf} = 75 mM as reference values in parameter estimation.

Assuming all the nitrogen atoms are derived from glutamate and glutamine, we obtain the following relationship between biosynthetic requirements and N_0.

$$GLU_{demn} + GLU_{demf} + GLN_{demn} + 2 \cdot GLN_{demf} = N_0 \, . \cdots\cdots \text{(S8.5-5)}$$

The left hand side is 3142 mM using the corrected values. As we mentioned in Section 8.1, we use N_0 = 3000 mM. Considering the fact that we calculated both sides of Eq. (S8.5-5) via different calculation procedures, some 5 % difference is negligible and it therefore validates our calculation.

9 Detailed Discussion on Comparison of Active and Passive Transporter Models

9.1 Passive transporter model requires at least 1600 mM/min for V_{max} of GS

For the passive transporter model to explain the specific growth rate of 0.8 h^{-1} at 4 μM external NH_4^+ level, which was observed in Kim's experiment, V_{max} of GS (V_{gs}) needs to be higher than 1600 mM/min. This statement is independent of the choice of parameter values. To achieve the specific growth rate of 0.8 h^{-1}, N-assimilation flux must be 40 mM/min (N_0 = 3 M). At 4 μM external NH_4^+, the theoretical maximum of internal NH_4^+ is 2.5 μM in the passive transporter model. With such a low internal NH_4^+, N-assimilation by GDH is negligible because its K_m for NH_4^+ is rather high (1.1 mM). GS must fulfil the nitrogen requirement of 40 mM/min. Since K_m of GS for NH_4^+ is 100 μM, the actual reaction rate is at most only 2.5 % of V_{gs} at 2.5 μM internal NH_4^+. To achieve the actual rate of 40 mM/min, V_{gs} must be 1600 mM/min even if the other substrates of GS (glutamate and ATP) would be saturating, and the products (glutamine, ADP, and Pi) would be zero.

9.2 Parameters that are different between the active and passive transporter models

As we showed in the main text, model plausibility for the active transporter model is 2.2 x 10^{-4}, and that for the passive transporter model is 1.7 x 10^{-6}. Therefore, we concluded that the active transporter model is 130 times more likely than the passive transporter model. To investigate what causes the 130-fold difference in model plausibility, we plotted the deviation of the averages of estimated values (*n* = 5; ±SD) from their reference values (**Figure S4**). Overall, the active and passive transporter models have similar parameter values. However, some parameters are different between the two models, which results in the difference in model plausibility. The difference in parameter values comes from the fact that the passive transporter model cannot accumulate NH_4^+ inside and thus needs GS-related parameters to be changed from the reference values in order to achieve an N-assimilation rate of 40 mM/min at a low internal NH_4^+ concentration.

GS_{total} (Kim) and k_{catgs} in the passive transporter model are estimated to be very high: 31 ± 0.7 μM (dodecamer) and 1750 ± 63 s^{-1} per dodecamer, respectively, leading to V_{gs} in excess of 3269 ± 76 mM/min (± SD). To the best of our knowledge, the highest GS concentration found in literature is 14 μM (dodecamer) [67]. Moreover, according to a proteomics study on 31 growth conditions [76], most of the abundant enzymes are related to central carbon metabolism. Malate dehydrogenase is the most abundant enzyme among 236 detected enzymes, and it amounts up to 200 μM (monomer). Nine enzymes are in between 60-84 μM (monomer) and 226 enzymes occur at less than 40 μM. Therefore, the estimated GS monomer concentration in excess of ~400 μM (= ~31 μM x 12) is too high to be realistic. In addition, ligases (EC numbers 6.-) have moderate k_{cat}'s: on average 6 s^{-1} (*n* = 17) and at most 15 s^{-1} per active site [76]. Thus, the estimated k_{cat} of GS (EC 6.3.1.2), i.e. ~146 s^{-1} per active site, is too high. Finally, to our knowledge, the highest reported value of V_{gs} is 600 mM/min [14], and most reported values are 200 – 300 mM/min (data not shown). For the active transporter model, GS_{total} (Kim) and k_{catgs} were estimated to be 11 ± 0.7 μM (dodecamer) and 878 ± 34 s^{-1} per dodecamer [i.e.137 ± 9 μM

(monomer) and 73 ± 3 s^{-1} per active site], leading to V_{gs} = 601 ± 49 mM/min (± SD). These values are relatively high, but not as high as those for the passive transporter model.

Not only GS_{total} (Kim) and k_{catgs} but also some other parameters are different between the active and passive transporter models. In the passive transporter model, GS_{total} (Yuan) is decreased to compensate an increase in k_{catgs} (V_{gs} needs to be ~330 mM/min to fit Yuan's experimental data). In the passive parameter model, to increase the reaction rate of GS, K_{gsatp} is reduced, and K_{gsadp} and K_{gspi} are slightly increased. Three ATase parameters (K_{adgs}, V_{dead}, and $K_{deadgsamp}$) are different between the active and passive transporter model, leading to different GS adenylylation profiles: In Kim's experiment, the active transporter model predicts that n_{AMP} changes between 0.2 – 7, but the passive transporter model predicts n_{AMP} changes between 0.1 – 11 (data not shown). The large change in n_{AMP} for the passive transporter model is probably required to deal with 274-fold change in internal NH_4^+ (2.3 μM – 0.63 mM). In the active transporter model, internal NH_4^+ changes only 37-fold (17 μM – 0.63 mM). In the passive transporter model, 2-oxoglutarate concentration (which is determined by two constants κ and OG_{basal}) is increased to keep AmtB active [see Eq. (S2.2.3-1)].

9.3 Low value for model plausibility is to be expected

As mentioned above, the model plausibility of the active transporter model is 2.2 x 10^{-4}, and that of the passive transporter model is 1.7 x 10^{-6}. One might argue that the plausibility of the active transporter model in absolute sense is already just too low, and that therefore the conclusion should be that not only the passive transporter model but also the active transporter model is unrealistic. However, we argue that the low value just implies that even the active transporter model cannot fit the training data without changing parameter values. As shown in **Figure S4**, most values are close to the reference values, but some are a little off and a few do substantially deviate; all together, this results in a low absolute value for the plausibility. Also, theoretically, a value of 2.2 x 10^{-4} can be obtained by assuming that all model parameters deviate by 44.7 % from their reference values, which means that they are all just ~1.45-fold higher or lower. Thus, for most realistic models, the fact of the matter is that a low value for the plausibility is to be expected.

9.4 Active transporter model is more likely even if values of λ_i are changed

The choice of values for the penalty weight λ_i affects model plausibility. We used λ_I = 1.0407, λ_{II} = 0.1930, and λ_{III} = 0, which correspond to σ_I = ln(2), σ_{II} = ln(5), and $\sigma_{III} \rightarrow \infty$, respectively (see Methods in the main text), and we reached the conclusion that the active transporter model is 130 times more likely than the passive transporter model. These penalty weight values are reasonable because a model is probably less realistic if more than 2-fold and 5-fold changes are required for a number of class I and class II parameters, respectively, to fit training experimental data. To investigate how the ratio of model plausibility of the active transporter model to that of the passive transporter model changes, we changed λ_i's and performed parameter estimation. We investigated the following three cases: (i) λ_I = 1.0407 and λ_{II} = 1.0407, (ii) λ_I = 0.1930 and λ_{II} = 1.0407, (iii) λ_I = 0.1930 and λ_{II} = 0.1930. We kept λ_{III} = 0 for all the cases. We obtained the following ratios of model plausibility of the active transporter model to that of the passive transporter model: (i) 516, (ii) 7948, and (iii) 3 [For (ii), the constraint violation γ could not be zero: the smallest γ values we obtained were 2.0 x 10^{-8} and

2.4 x 10^{-5} for active and passive, respectively]. Therefore, our conclusion that the active transporter model is more likely than the passive transporter model holds at least qualitatively even if we change values of λ_i.

9.5 Active transporter model is more likely even if K_m of GS for NH_4^+ is allowed to be changed

In parameter estimation above and in the main text, we considered K_m of GS for NH_4^+ (K_{gsnh}) as a constant (i.e. an unsearched parameter) because there is broad consensus in the literature on its value (100 μM). Is the active transporter model more likely than the passive transporter model even if we allow K_{gsnh} to be changed? To answer this question, we conducted an additional parameter search in which we made K_{gsnh} a class I parameter so that K_{gsnh} would be searched. We used $\lambda_I = 1.0407$, $\lambda_{II} = 0.1930$, and $\lambda_{III} = 0$. Model plausibility of the active transporter model was significantly higher than that of the passive transporter model ($p = 0.008$, Wilcoxon rank-sum test): 3.4 x 10^{-4} ± 4.7 x 10^{-5} vs. 1.9 x 10^{-5} ± 1.5 x 10^{-6} ($n = 5$; ± SD). Thus, even if we allow K_{gsnh} to be changed, the active transporter model is 18 times more likely than the passive transporer model. For the active transporter model, K_{gsnh}, GS_{total} (Kim), and k_{catgs} were estimated to be 65 ± 3 μM, 10 ± 0.6 μM (dodecamer), and 797 ± 18 s^{-1} per dodecamer, respectively, and V_{gs} = 483 ± 33 mM/min (± SD). For the passive transporter model, those were estimated to be 32 ± 1 μM, 18 ± 0.5 μM (dodecamer), and 1117 ± 76 s^{-1} per dodecamer, respectively, and V_{gs} = 1221 ± 57 mM/min. In reality, it is unlikely that the K_{gsnh} is lower than the value we employed (0.1 mM) because (i) the K_{gsnh} in the presence of 50-100 mM glutamate was actually 0.2 mM [57] and (ii) in general, most K_m values for small hydrophilic substrates exceed ~0.2 mM (See Figure 3 of [77]; for NH_4^+, molecular weight = 18 and $\log P_{o/w} = 0.38$).

10 Why Are the Metabolome Phenotypes Similar for ΔAmtB and Wild Type in Yuan's Experiments?

Since the refined active transporter model includes the ammonium transporter AmtB, it can predict the behaviours of ΔAmtB, which is one of the advantages over the Yuan model, which does not contain the transporter. Yuan reported that metabolome phenotypes of ΔAmtB cells were indistinguishable from those of wild type (see Supplementary Figure 5 of [1]). The refined active transporter model explains the reason for the (unexpected) indistinguishable behaviour of wild type and ΔAmtB: Before N-upshift, for cells growing on filters lying on top of agarose nutrient plates, NH_x diffusion from the solid medium to the cell surface is the rate-limiting step of N-assimilation [$\Delta\ln(v_{gdh}+v_{gs})/\Delta\ln(k_{db}) = \sim 1$] and consequently the presence or absence of AmtB does not influence the NH_x uptake rate. After N-upshift, AmtB is completely blocked by GlnK in the wild type, and thus its metabolome phenotypes are almost indistinguishable from those of ΔAmtB.

11 Model Improvements and Summary of Other Models

11.1 Model improvements

The following is a list of the improvements that were implemented in the models we made for this paper; most corrections relate to errors made by ourselves and by others in the past.

1. The AmtB-mediated ammonium transport kinetics is consistent with the detailed balance principle, i.e. $[NH_4^+{}_{int}]/[NH_4^+{}_{ext}] = \varphi$ at $v_{amtb} = 0$ (see Section 7). This was not explicitly considered in our previous study

[78].

2. In general, pK_a depends on the temperature. Ammonium/ammonia $pK_a = 9.25$ has been used in many studies [6, 14, 78, 79]. However, $pK_a = 9.25$ is only correct at 25 °C. We have used $pK_a = 8.95$ for Yuan's and Kim's experiments (37 ºC) and 9.09 for Radchenko's experiment (30 ºC). This difference in pK_a is important because it significantly affects the estimation of the magnitude of AmtB-mediated ammonium transport and unfacilitated NH_3 diffusion. The ammonium/ammonia futile cycling with pKa = 8.95 is more extensive than previously estimated with $pK_a = 9.25$.
3. In our previous models [14, 78], the glutamate level was 0.1 – 50 mM, which is too low to be realistic. In this paper, the glutamate level is 70 – 150 mM, which is consistent with recent studies [1, 28].
4. In our previous study [14], it was assumed that the 2-oxoglutarate level was constant upon N-change, i.e. 0.2 mM or 1.0 mM. In this paper, the 2-oxoglutarate level increases at decreasing N-availability, which is consistent with [1].
5. In our previous study [14], glutamine was ~1 mM both at 50 µM and at 1 mM ammonium. In this paper, glutamine increases some 10-fold (1 to 10 mM) upon a 10 mM ammonium-upshift, which is consistent with [1].
6. AmtB and its regulation by GlnK through both 2-oxoglutarate binding and uridylylation are modeled. This was not done in our previous studies [14, 78].
7. In this paper, a realistic cell area and cell volume are used. Assuming that *E. coli* is rod-shaped (perfectly spherical at the two ends) with a radius of 0.54 µm and a total length of 2.7 µm, we use $A_{cell} = 9.18\ \mu m^2$ and $V_{cell} = 2.15\ \mu m^3$ (see Section 8.2). In our previous studies [14, 78], a radius of 0.3 µm and a total length of 3 µM were assumed, and $A_{cell} = 5.6\ \mu m^2$ and $V_{cell} = 0.79\ \mu m^3$ were used.
8. In this paper, a realistic value of N-content of N-limited cells (N_0) is used: 3 mol-N/L-cyt (see Section 8.1). Previously, $N_0 = 5$ mol-N/L-cyt has been used by us [14, 23].
9. The rate equation of UTase used in [14] to describe uridylylation of GlnB (PII) was not entirely correct. In this paper, the right rate equation of UTase, both for GlnB and for GlnK, is employed [80].
10. In this paper, GlnK sequestration by AmtB, which prevents GlnK from being uridylylated [25], was taken into account. This was not modeled in our previous study [78].

11.2 Summary of other models

Many computational models for the *E. coli* ammonium assimilation network have been reported [1, 11, 14, 25, 78, 81-88]. Most of them focus on a certain part of the network: The metabolic reactions [81, 88], the GS-ATase monocycle [86], the GlnB-UTase monocycle with NRII [87], or the PII-UTase-GS-ATase bicycle [82, 84]. Kurata et al. [11, 83] and Mutalik et al. [85] presented mathematical models that consisted of metabolic reactions, enzyme activity modifications, and gene regulations. They investigated how enzyme activity and gene regulations cooperatively respond to changes in nitrogen availability. However, their models have not been compared with experimental data. Moreover, their models do not include the ammonium transporter AmtB. The models in this paper are based on our previous models [14, 78]. Bruggeman et al. proposed a dynamic model based on detailed enzyme kinetics [14]. Ma et al. extended the Bruggeman model to include the AmtB-mediated ammonium transport and unfacilitated NH_3 diffusion [78]. However, their model contained

an error; the set of reactions constituting ammonium transport did not comply with the detailed balance principle [89]. Although the Bruggeman and Ma models captured qualitative or semi-quantitative behaviors known to exist at the time, they have not been challenged with more recent quantitative experimental data.

Yuan developed a kinetic model of *E. coli* ammonium assimilation, which includes GDH, GOGAT, GS, and the PII-UTase-GS-ATase bicycle, but not AmtB-mediated ammonium transport or gene expression regulations [1]. Yuan reported that their model successfully reproduced the transient dynamic of glutamate, glutamine, and aspartate upon N-perturbation. We used their transient metabolome data upon N-perturbation as training data. The Yuan model is a great step toward the holistic understanding of the ammonium assimilation. However, we think their model is incomplete: (i) NH_4^+ and NH_3 were not discriminated in their model. (ii) The intracellular NH_4^+ was calculated to be as low as ~1 μM for a specific growth rate of ~0.2 h^{-1}. (iii) V_{max} of GS had a high value of 9120 mM/min. (iv) PII uridylylation state was not responsive to the N-status. (v) The number of nitrogen atoms per cell volume (N_0) was calculated to be ~6 M for N-limited *E. coli* cells. (vi) Their model did not reach a steady state before N-perturbation.

Wang et al. [88] performed flux balance analysis based on Yuan's experimental data. In contrast to our simulation that focuses on the metabolic changes after N-upshift at 180 min in Yuan's experiments (Figure 2A of [1]), their simulation was dedicated to the time range from 0 min to 180 min. They estimated V_{max} of GS as high as ~10,000 mM/min, but we think this is an overestimation: (i) Wang et al. overestimated glutamate and glutamine consumption fluxes because of a small cell volume (0.7 μm^3) and introduced a miscalculation while converting cellular compositions into consumption fluxes [they used a factor of $1/\tau$ instead of $\ln(2)/\tau$]. (ii) Their model did not contain AmtB, and thus the internal NH_4^+ can be as low as 10 μM, but still they assumed that *E. coli* at such a low internal NH_4^+ grows as fast as under N-rich condition.

Recently, Gosztolai et al. developed a concise model of *E. coli's* ammonium assimilation and investigated the role of GlnK in detail [25]. Their model consists of three variables: GlnBUMP, GlnKUMP, and GSAMP. Their model requires 2-oxoglutarate, glutamine, total GlnB, total GlnK, and total GS concentrations as model inputs and predicts posttranslational modification states of GlnB, GlnK, and GS. Their model does not include ammonium/ammonia, glutamate, GDH, GOGAT, AmtB, or metabolic reactions. Since the models in our study focus on short-term transient responses [1, 10] or steady states [6], we did not use their experimental data (long-term transient responses with changes in gene expressions) as training data in our modeling.

12 Constrained Optimization-based Approach vs. Conventional Approach

Conventionally, a parameter estimation problem is formulated as an (unconstrained) optimization problem [compare the following equations with Eqs. (3) in the main text]:

$$\text{minimize} \quad f_{conv}(\mathbf{p}), \quad \cdots\cdots \quad \text{(S12-1a)}$$

$$\text{subject to} \quad \mathbf{p}^L \le \mathbf{p} \le \mathbf{p}^U, \quad \cdots\cdots \quad \text{(S12-1b)}$$

where $\mathbf{p} = (p_1, p_2, ...)$ is the search parameter vector. f_{conv} is the (conventional) objective function that indicates badness-of-fit to training data (typically, the sum of squared residuals between experimental values and

simulated values). $\mathbf{p}^L$ and $\mathbf{p}^U$ are the lower bound and upper bound vectors, respectively. The aim of this (unconstrained) optimization problem is to minimize the badness-of-fit (i.e. to maximize the goodness-of-fit). In a few studies (e.g. [51, 90]), parameter estimation problems have been formulated as a constrained optimization problem; however, in those studies, the objective function was used as a badness-of-fit indicator as in Eqs. (S12-1), and constraint functions were used to incorporate the relationships among parameters (e.g. a certain parameter is larger than another). In contrast, in this study, we use the objective function to quantify parameter deviation from the reference values and the constraint functions to quantify the badness-of-fit.

The conventional approach based on Eqs. (S12-1) suffers from the following three problems. First, it cannot incorporate prior knowledge about parameter values efficiently: what one can do is to believe it without any doubt or not to believe it at all. If a certain parameter has been measured *in vitro*, one might fix it to the measured value during parameter estimation. However, if the parameter value *in vitro* is very different from that *in vivo*, the fixation prevents a model from fitting to training data. Indeed, parameter values *in vivo* can be very different from those *in vitro* [66]. On the other hand, if the measured parameter is searched during parameter estimation, the parameter can take any value within the lower and upper bounds without any penalties. To achieve good fitting with reasonable parameter values, the best one can do is to choose appropriate lower and upper bounds by trial and error. Our approach [Eqs. (3)] can incorporate prior knowledge about parameter values as reference values and penalty weights (λ_j). We allow parameters to change with penalties (within a wide search space).

Second, the conventional approach [Eqs. (S12-1)] provides little information about model plausibility. In our approach [Eqs. (3)], since f is formulated as the natural logarithm of the inverse of model plausibility, model plausibility can be calculated by the f value (accompanied by $\gamma = 0$).

Third, the conventional approach [Eqs. (S12-1)] often suffers from the parameter non-identifiability problem. In parameter estimation, it is important to uniquely determine a parameter set $\mathbf{p}$. If multiple parameter sets provide similar fittings (i.e. similar f_{conv} values), then the prediction based on a single parameter set will not be reliable. $\mathbf{p}$ can be uniquely determined only if the model is structurally and practically identifiable [91, 92]. If parameters compensate for each other's effect on model behavior, they cannot be uniquely determined (structurally non-identifiable). If there are fewer experimental data points than search parameters, these parameters cannot be uniquely determined (practically non-identifiable). Generally, it is difficult for the conventional approach to avoid parameter non-identifiability in realistic kinetic modeling. The conventional approach pursues good fitting only. In contrast, our constrained optimization-based approach [Eqs. (3)] not only looks for good fitting but also minimizes parameter deviation from the reference values. The latter additional requirement can help to identify a particular parameter set (see Section 13 and **Figure S6**). This technique is called "regularization" [92]. The regularization is analogous to the maximization of the objective function (e.g. biomass yield and ATP production) in flux balance analysis (FBA): Maximizing the objective function, one can determine a particular flux distribution (i.e. is identifiable). Without the objective function, the flux distribution cannot be uniquely determined (i.e. is non-identifiable). The difference between FBA and

our constrained optimization-based approach is that the former and the latter are linear and non-linear optimization problems, respectively. For FBA, optimization algorithms guarantee global optimality of solutions. However, for our constrained optimization-based approach (and the conventional approach as well), no practical optimization algorithms can do so. The best we can do is to repeatedly apply optimization algorithms and investigate whether they find the same (or at least a similar) solution.

13 Parameter Identifiability

In our constrained optimization-based approach, the quality of estimated parameter sets is expressed by both the objective function f (the quality of parameter values) and the constraint violation γ (the quality of model behavior). Thus, parameters affecting f or γ, or both, can be estimated. In the current study, in principle, class I and II parameters are identifiable while identifiability of class III parameters is not guaranteed.

Since we use the allowable error ε for constraint functions g_i (see Section 4.2), g_i can become zero for multiple parameter sets. That is, there are parameter spaces in which the γ value is zero and insensitive to changes in search parameters. Such parameter spaces are called feasible regions (the shaded areas in **Figure S6**). Indeed, for the parameter sets with $\gamma = 0$, the sensitivities of γ are almost zero ($\Delta\gamma/\Delta p_i \approx 0$, see **Table S11**). When $\gamma > 0$, the sensitivity can be non-zero **(Table S11**). The aim of using constraint functions g_i (and the constraint violation γ) is not to identify search parameters but to limit the feasible parameter space.

The sensitivity of f to search parameters (class I - III) can be analytically calculated by differentiating Eq. (S4.1-1):

$$\frac{\partial f(\mathbf{p})}{\partial p_i} = \frac{2\lambda_i}{p_i} \ln \frac{p_i}{p_i^*}, \quad \cdots\cdots (S13\text{-}1)$$

where $\mathbf{p} = (p_1, p_2, \ldots)$ is the search parameter vector, and p_i is the ith parameter. p_i^* is the reference value of the ith parameter. λ_j ($j = I, II, III$) is the class-related penalty weight for a parameter change: $\lambda_i = 1.0407, 0.1930$, and 0 for class I, II, and III, respectively. For class I and II parameters, the sensitivity [Eq. (S13-1)] is non-zero unless $p_i = p_i^*$ for all i, meaning that changes in class I and II parameters almost always cause changes in the f value. If the constraints ($g_i \leq 0$ for all i) are combined with the minimization of the objective function f, class I and II parameters are identifiable because in practice f takes the minimum at a single point in the feasible regions (see **Figure S6b**). For class III parameters, the sensitivity [Eq. (S13-1)] is zero, indicating that class III parameters do not have any effect on the f value. Thus, class III parameters are less identifiable: their values are estimated based only on their effect on the γ value (model behavior).

Even if parameters are identifiable, they cannot always be computationally identified, as will be explained below. In the problem of minimizing $f(x) = x^2$ with no constraints, $f(x) = 0$ is the unique global optimum, and x is identifiable ($x = 0$). For such a simple problem, it is easy for optimization algorithms to find the global optimum and identify x. However, this is not always the case. If x is identifiable but $f(x)$ is very complicated (e.g. nonlinear, multivariate, and having multiple local optima), it is computationally difficult to find the global optimum and identify x. For such difficult problems, population-based stochastic algorithms such as genetic

algorithms (GAs) are used. They work better than gradient-based algorithms [44, 46]; however, they do not guarantee the global optimality of solutions. Indeed, the values of class I and II parameters are slightly different for each GA trial (**Table S10**), although they are identifiable in principle as discussed above. Non-identifiable parameters cannot be identified regardless of the optimization algorithms used. For example, in the problem of minimizing $f(x)$ = max(0,x^2-1), the global optimum is $f(x) = 0$ with $-1 \le x \le 1$: x is non-identifiable and cannot be uniquely determined whatever algorithm is used.

14 Rationale for the Implementation of Model Plausibility and Objective Function

We define the objective function f as the natural logarithm of the inverse of model plausibility (MP):

$$\begin{aligned} f(\mathbf{p}) &= \ln\left[MP(\mathbf{p})^{-1}\right] \\ &= \ln\left(\left[\prod_{i=1}^{n}\exp\left(-\frac{\left[\ln(p_i/p_i^*)\right]^2}{2\sigma_i^2}\right)\right]^{-1}\right) \\ &= \sum_{i=1}^{n}\frac{\left[\ln(p_i/p_i^*)\right]^2}{2\sigma_i^2} \\ &= \sum_{i=1}^{n}\lambda_i\left(\ln\frac{p_i}{p_i^*}\right)^2 \end{aligned}, \quad \text{(S14-1)}$$

where $\mathbf{p} = (p_1, p_2, \ldots)$ is the search parameter vector, and p_i is the ith parameter. p_i^* is the reference value of the ith parameter. We use "inverse" and "natural logarithm" to simplify the objective function and to make the objective function more tractable for genetic algorithms (GAs). Importantly, the minimization of the objective function is equal to the maximization of the model plausibility.

Note that model plausibility is (normalized) likelihood widely used in the maximum likelihood estimation. We assume that $\ln(p_i/p_i^*)$ follows the normal distribution (we describe the reason below). Model likelihood (ML) is given by:

$$ML(\mathbf{p}) = \prod_{i=1}^{n} PDF_i(p_i) = \prod_{i=1}^{n}\frac{1}{\sqrt{2\pi\sigma_i^2}}\exp\left(-\frac{\left[\ln(p_i/p_i^*)\right]^2}{2\sigma_i^2}\right). \quad \text{(S14-2)}$$

Since the normalized mean μ is zero in our context [$\ln(p_i^*/p_i^*) = 0$], it does not appear in Eq. (S14-2). In the conventional maximum likelihood estimation, p_i and p_i^* are given, and σ_i are estimated by maximizing ML. In our constrained optimization-based approach, p_i^* and σ_i are given, and p_i is estimated. If $p_i = p_i^*$ for all i (i.e. all parameter values are equal to their reference values), then ML becomes:

$$ML(\mathbf{p}^*) = \prod_{i=1}^{n} PDF_i(p_i^*) = \prod_{i=1}^{n}\frac{1}{\sqrt{2\pi\sigma_i^2}}. \quad \text{(S14-3)}$$

Model plausibility (MP) is the normalized ML, i.e. the ratio of Eq. (S14-2) to Eq. (S14-3):

$$MP(\mathbf{p}) = \frac{ML(\mathbf{p})}{ML(\mathbf{p}^*)} = \prod_{i=1}^{n} \exp\left(-\frac{\left[\ln(p_i / p_i^*)\right]^2}{2\sigma_i^2} \right), \quad \text{(S14-4)}$$

Therefore, the maximization of *MP*(**p**) is equal to the maximization of *ML*(**p**). That is, our constrained optimization-based approach maximizes (model) likelihood in the same way as the conventional maximum likelihood estimation.

As mentioned above, we assumed that $\ln(p_i/p_i^*)$ follows the normal distribution. The reference values are not "true" values; however, if estimated values are far from the reference values, the estimated values can be considered less realistic. Therefore, the probability density must form a single peak at $p_i/p_i^* = 1$ and decreases with distance from p_i^*. Among simple distributions satisfying this requirement is the normal distribution for $\ln(p_i/p_i^*)$.

Even assuming different probability distributions, the main results do not change as long as they yield the single peak at $p_i/p_i^* = 1$ and decreases with distance from it. We will discuss two examples of alternative probability distributions below.

Example 1: Assuming p_i/p_i^* [instead of $\ln(p_i/p_i^*)$] follows the normal distribution, the probability density function (*PDF*), parameter plausibility (*PP*), model plausibility (*MP*), and objective function (*f*) become:

$$PDF_i(p_i) = \frac{1}{\sqrt{2\pi\sigma_i^2}} \exp\left[-\frac{\left(p_i / p_i^* - 1\right)^2}{2\sigma_i^2} \right], \quad \text{(S14-5)}$$

$$\begin{aligned} PP_i(p_i) &= \frac{PDF_i(p_i)}{PDF_i(p_i^*)} \\ &= \exp\left[-\frac{\left(p_i / p_i^* - 1\right)^2}{2\sigma_i^2} \right] \end{aligned}, \quad \text{(S14-6)}$$

$$\begin{aligned} MP(\mathbf{p}) &= \prod_{i=1}^{n} PP_i(p_i) \\ &= \prod_{i=1}^{n} \exp\left[-\frac{\left(p_i / p_i^* - 1\right)^2}{2\sigma_i^2} \right] \end{aligned}, \quad \text{(S14-7)}$$

$$\begin{aligned} f(\mathbf{p}) &= \ln\left[MP(\mathbf{p})^{-1} \right] \\ &= \sum_{i=1}^{n} \frac{\left(p_i / p_i^* - 1\right)^2}{2\sigma_i^2} \\ &= \sum_{i=1}^{n} \lambda_i \left(\frac{p_i}{p_i^*} - 1 \right)^2 \end{aligned}, \quad \text{(S14-8)}$$

where

$$\lambda_i = \frac{1}{2\sigma_i^2} \quad \text{(S14-9)}$$

We reperformed parameter estimation for the active and the passive transporter models using Eq. (S14-8). We used $\lambda_i = 0.1250$ for class I, $\lambda_i = 0.0200$ for class II, and $\lambda_i = 0$ for class III parameters. These values correspond to $\sigma_i = 2$, $\sigma_i = 5$, and $\sigma_i \rightarrow \infty$, respectively. We obtained $MP = 0.62 \pm 0.01$ and 0.22 ± 0.03 for the active and the passive transporter models, respectively ($n = 2$ and 3; ± SD): the model plausibility of the active transporter model is 2.8 times higher than that of the passive transporter model. Therefore, our conclusion holds (at least qualitatively) even if we assume p_i/p_i^* follows the normal distribution. We prefer the normal distribution for $\ln(p_i/p_i^*)$ to that for p_i/p_i^* because in the latter $p_i/p_i^* = \alpha$ and $p_i/p_i^* = \alpha^{-1}$ are differently penalized: $p_i/p_i^* = \alpha$ is more penalized than $p_i/p_i^* = \alpha^{-1}$ even though an α–fold change ($\alpha > 1$) applies to both. If $\ln(p_i/p_i^*)$ follows the normal distribution as we assume in the main text, both are equally penalized.

Example 2: If we assume p_i/p_i^* follows the gamma distribution, then the probability density function (*PDF*), parameter plausibility (*PP*), model plausibility (*MP*), and objective function (*f*) become:

$$PDF_i(p_i) = \frac{1}{\Gamma(k_i)\cdot\theta^{k_i}}\left(\frac{p_i}{p_i^*}\right)^{k_i-1}\exp\left(-\frac{p_i}{\theta_i p_i^*}\right), \quad \text{(S14-10)}$$

$$\begin{aligned} PP_i(p_i) &= \frac{PDF_i(p_i)}{PDF_i(p_i^*)} \\ &= \left(\frac{p_i}{p_i^*}\right)^{k_i-1}\exp\left[\frac{1}{\theta_i}\left(1-\frac{p_i}{p_i^*}\right)\right], \end{aligned} \quad \text{(S14-11)}$$

$$\begin{aligned} MP(\mathbf{p}) &= \prod_{i=1}^{n} PP_i(p_i) \\ &= \prod_{i=1}^{n}\left[\left(\frac{p_i}{p_i^*}\right)^{k_i-1}\exp\left[\frac{1}{\theta_i}\left(1-\frac{p_i}{p_i^*}\right)\right]\right], \end{aligned} \quad \text{(S14-12)}$$

$$\begin{aligned} f(\mathbf{p}) &= \ln\left[MP(\mathbf{p})^{-1}\right] \\ &= \ln\left[\prod_{i=1}^{n}\left(\frac{p_i}{p_i^*}\right)^{1-k_i}\right] + \sum_{i=1}^{n}\left[\frac{1}{\theta_i}\left(\frac{p_i}{p_i^*}-1\right)\right]. \end{aligned} \quad \text{(S14-13)}$$

Γ is the gamma function. k_i and θ_i are the shape and the scale parameters, respectively, for the gamma distribution. We reperformed parameter estimation for the active and the passive transporter models using Eq. (S14-13). We used $k_i = 1.6404$ and $\theta_i = 1.5615$ for class I parameters, $k_i = 1.2210$ and $\theta_i = 4.5249$ for class II parameters, and $k_i = 1$ and $\theta_i \rightarrow \infty$ for class III parameters. With these parameters, PDF_i provides the peak at $p_i/p_i^* = 1$, and the standard deviations of 2, 5 and infinity for class I, II, and III parameters, respectively. We obtained $MP = 4.1 \times 10^{-2} \pm 4.3 \times 10^{-3}$ and $7.3 \times 10^{-3} \pm 2.3 \times 10^{-4}$ for the active and the passive transporter models, respectively ($n = 4$ and 5; ± SD): the model plausibility of the active transporter model is 5.6 times higher than that of the passive transporter model. Therefore, our conclusion holds even if we assume p_i/p_i^*

follows the gamma distribution. We prefer the normal distribution for $\ln(p_i/p_i^*)$ to the gamma distribution for p_i/p_i^*: (i) the objective function for the former [Eq. (S4.1-1)] is much simpler than that for the latter [Eq. (S14-13)]. (ii) It is not straight-forward to assign reasonable values for k_i and θ_i. (iii) $p_i/p_i^* = \alpha$ and $p_i/p_i^* = \alpha^{-1}$ are differently penalized in Eq. (S14-13). The gamma distribution is defined only for positive random variables, and thus we cannot use the gamma distribution for $\ln(p_i/p_i^*)$.

References

1. Yuan, J., Doucette, C. D., Fowler, W. U., Feng, X. J., Piazza, M., Rabitz, H. A., Wingreen, N. S. & Rabinowitz, J. D. (2009) Metabolomics-driven quantitative analysis of ammonia assimilation in E. coli, *Mol Syst Biol.* **5**, 302.
2. Yuan, J., Fowler, W. U., Kimball, E., Lu, W. & Rabinowitz, J. D. (2006) Kinetic flux profiling of nitrogen assimilation in Escherichia coli, *Nature chemical biology.* **2**, 529-30.
3. Yuan, J., Bennett, B. D. & Rabinowitz, J. D. (2008) Kinetic flux profiling for quantitation of cellular metabolic fluxes, *Nature protocols.* **3**, 1328-40.
4. Bennett, B. D., Yuan, J., Kimball, E. H. & Rabinowitz, J. D. (2008) Absolute quantitation of intracellular metabolite concentrations by an isotope ratio-based approach, *Nature protocols.* **3**, 1299-311.
5. Picioreanu, C., van Loosdrecht, M. C. M. & Heijnen, J. J. (1997) Modelling the effect of oxygen concentration on nitrite accumulation in a biofilm airlift suspension reactor, *Water Science and Technology.* **36**, 147.
6. Kim, M., Zhang, Z., Okano, H., Yan, D., Groisman, A. & Hwa, T. (2012) Need-based activation of ammonium uptake in Escherichia coli, *Mol Syst Biol.* **8**, 616.
7. Radzikowski, J. L., Vedelaar, S., Siegel, D., Ortega, A. D., Schmidt, A. & Heinemann, M. (2016) Bacterial persistence is an active sigmaS stress response to metabolic flux limitation, *Mol Syst Biol.* **12**, 882.
8. Volkmer, B. & Heinemann, M. (2011) Condition-dependent cell volume and concentration of Escherichia coli to facilitate data conversion for systems biology modeling, *PLoS One.* **6**, e23126.
9. Runarsson, T. P. & Yao, X. (2000) Stochastic ranking for constrained evolutionary optimization, *IEEE Transactions on Evolutionary Computation.* **4**, 284-294.
10. Radchenko, M. V., Thornton, J. & Merrick, M. (2014) Association and dissociation of the GlnK-AmtB complex in response to cellular nitrogen status can occur in the absence of GlnK post-translational modification, *Frontiers in microbiology.* **5**, 731.
11. Kurata, H., Masaki, K., Sumida, Y. & Iwasaki, R. (2005) CADLIVE dynamic simulator: direct link of biochemical networks to dynamic models, *Genome Res.* **15**, 590-600.
12. Kurata, H., Matoba, N. & Shimizu, N. (2003) CADLIVE for constructing a large-scale biochemical network based on a simulation-directed notation and its application to yeast cell cycle, *Nucleic Acids Res.* **31**, 4071-84.
13. Kurata, H., Inoue, K., Maeda, K., Masaki, K., Shimokawa, Y. & Zhao, Q. (2007) Extended CADLIVE: a novel graphical notation for design of biochemical network maps and computational pathway analysis, *Nucleic Acids Res.* **35**, e134.
14. Bruggeman, F. J., Boogerd, F. C. & Westerhoff, H. V. (2005) The multifarious short-term regulation of ammonium assimilation of Escherichia coli: dissection using an in silico replica, *FEBS J.* **272**, 1965-85.
15. Kingdon, H. S., Shapiro, B. M. & Stadtman, E. R. (1967) Regulation of glutamine synthetase. 8. ATP: glutamine synthetase adenylyltransferase, an enzyme that catalyzes alterations in the regulatory properties of glutamine synthetase, *Proc Natl Acad Sci U S A.* **58**, 1703-10.
16. Ninfa, A. J., Jiang, P., Atkinson, M. R. & Peliska, J. A. (2000) Integration of antagonistic signals in the regulation of nitrogen assimilation in Escherichia coli, *Curr Top Cell Regul.* **36**, 31-75.
17. Jaggi, R., van Heeswijk, W. C., Westerhoff, H. V., Ollis, D. L. & Vasudevan, S. G. (1997) The two opposing activities of adenylyl transferase reside in distinct homologous domains, with intramolecular signal transduction, *EMBO J.* **16**, 5562-71.

18. Jiang, P., Peliska, J. A. & Ninfa, A. J. (1998) The regulation of Escherichia coli glutamine synthetase revisited: role of 2-ketoglutarate in the regulation of glutamine synthetase adenylylation state, *Biochemistry.* **37**, 12802-10.
19. Jiang, P., Peliska, J. A. & Ninfa, A. J. (1998) Enzymological characterization of the signal-transducing uridylyltransferase/uridylyl-removing enzyme (EC 2.7.7.59) of Escherichia coli and its interaction with the PII protein, *Biochemistry.* **37**, 12782-94.
20. Javelle, A., Severi, E., Thornton, J. & Merrick, M. (2004) Ammonium sensing in Escherichia coli. Role of the ammonium transporter AmtB and AmtB-GlnK complex formation, *J Biol Chem.* **279**, 8530-8.
21. Coutts, G., Thomas, G., Blakey, D. & Merrick, M. (2002) Membrane sequestration of the signal transduction protein GlnK by the ammonium transporter AmtB, *EMBO J.* **21**, 536-45.
22. Radchenko, M. V., Thornton, J. & Merrick, M. (2013) P(II) signal transduction proteins are ATPases whose activity is regulated by 2-oxoglutarate, *Proc Natl Acad Sci U S A.* **110**, 12948-53.
23. Boogerd, F. C., Ma, H., Bruggeman, F. J., van Heeswijk, W. C., Garcia-Contreras, R., Molenaar, D., Krab, K. & Westerhoff, H. V. (2011) AmtB-mediated NH3 transport in prokaryotes must be active and as a consequence regulation of transport by GlnK is mandatory to limit futile cycling of NH4(+)/NH3, *FEBS Lett.* **585**, 23-8.
24. van Heeswijk, W. C., Westerhoff, H. V. & Boogerd, F. C. (2013) Nitrogen assimilation in Escherichia coli: putting molecular data into a systems perspective, *Microbiol Mol Biol Rev.* **77**, 628-95.
25. Gosztolai, A., Schumacher, J., Behrends, V., Bundy, J. G., Heydenreich, F., Bennett, M. H., Buck, M. & Barahona, M. (2017) GlnK Facilitates the Dynamic Regulation of Bacterial Nitrogen Assimilation, *Biophys J.* **112**, 2219-2230.
26. Stoker, M. G. (1973) Role of diffusion boundary layer in contact inhibition of growth, *Nature.* **246**, 200-3.
27. Durand, A. & Merrick, M. (2006) In vitro analysis of the Escherichia coli AmtB-GlnK complex reveals a stoichiometric interaction and sensitivity to ATP and 2-oxoglutarate, *J Biol Chem.* **281**, 29558-67.
28. Bennett, B. D., Kimball, E. H., Gao, M., Osterhout, R., Van Dien, S. J. & Rabinowitz, J. D. (2009) Absolute metabolite concentrations and implied enzyme active site occupancy in Escherichia coli, *Nature chemical biology.* **5**, 593-9.
29. Penfound, T. & Foster, J. W. (1996) Biosynthesis and recycling of NAD in *Escherichia coli and Salmonella: cellular and molecular biology* (Neidhardt, F. C., ed) pp. 721-730, ASM Press, Washington (D. C.).
30. Andersen, K. B. & von Meyenburg, K. (1977) Charges of nicotinamide adenine nucleotides and adenylate energy charge as regulatory parameters of the metabolism in Escherichia coli, *J Biol Chem.* **252**, 4151-6.
31. Fuhrer, T. & Sauer, U. (2009) Different biochemical mechanisms ensure network-wide balancing of reducing equivalents in microbial metabolism, *J Bacteriol.* **191**, 2112-21.
32. Auriol, C., Bestel-Corre, G., Claude, J. B., Soucaille, P. & Meynial-Salles, I. (2011) Stress-induced evolution of Escherichia coli points to original concepts in respiratory cofactor selectivity, *Proc Natl Acad Sci U S A.* **108**, 1278-83.
33. Kumar, R. & Shimizu, K. (2010) Metabolic regulation of Escherichia coli and its gdhA, glnL, gltB, D mutants under different carbon and nitrogen limitations in the continuous culture, *Microb Cell Fact.* **9**, 8.
34. Soupene, E., He, L., Yan, D. & Kustu, S. (1998) Ammonia acquisition in enteric bacteria: physiological role of the ammonium/methylammonium transport B (AmtB) protein, *Proc Natl Acad Sci U S A.* **95**, 7030-4.
35. Yan, D., Lenz, P. & Hwa, T. (2011) Overcoming fluctuation and leakage problems in the quantification of intracellular 2-oxoglutarate levels in Escherichia coli, *Applied and environmental microbiology.* **77**, 6763-71.

36. Radchenko, M. V., Thornton, J. & Merrick, M. (2010) Control of AmtB-GlnK complex formation by intracellular levels of ATP, ADP, and 2-oxoglutarate, *J Biol Chem.* **285**, 31037-45.
37. Jahan, N., Maeda, K., Matsuoka, Y., Sugimoto, Y. & Kurata, H. (2016) Development of an accurate kinetic model for the central carbon metabolism of Escherichia coli, *Microb Cell Fact.* **15**, 112.
38. Vo, J., Inwood, W., Hayes, J. M. & Kustu, S. (2013) Mechanism for nitrogen isotope fractionation during ammonium assimilation by Escherichia coli K12, *Proc Natl Acad Sci U S A.* **110**, 8696-701.
39. Schneider, C. A., Rasband, W. S. & Eliceiri, K. W. (2012) NIH Image to ImageJ: 25 years of image analysis, *Nature methods.* **9**, 671-5.
40. Reitzer, L. (2003) Nitrogen assimilation and global regulation in Escherichia coli, *Annu Rev Microbiol.* **57**, 155-76.
41. Walker, M. C. & van der Donk, W. A. (2016) The many roles of glutamate in metabolism, *J Ind Microbiol Biotechnol.* **43**, 419-30.
42. Banga, J. R. (2008) Optimization in computational systems biology, *BMC Syst Biol.* **2**, 47.
43. Reali, F., Priami, C. & Marchetti, L. (2017) Optimization Algorithms for Computational Systems Biology, *Frontiers in Applied Mathematics and Statistics.* **3**.
44. Moles, C. G., Mendes, P. & Banga, J. R. (2003) Parameter estimation in biochemical pathways: a comparison of global optimization methods, *Genome Res.* **13**, 2467-74.
45. Egea, J. A., Emmanuel, V., Banga, J. R. & Martí, R. (2009) Improved scatter search for the global optimization of computationally expensive dynamic models, *J Global Optim.* **43**, 175-190.
46. Mendes, P. & Kell, D. (1998) Non-linear optimization of biochemical pathways: applications to metabolic engineering and parameter estimation, *Bioinformatics.* **14**, 869-83.
47. Sun, J. Y., Garibaldi, J. M. & Hodgman, C. (2012) Parameter Estimation Using Metaheuristics in Systems Biology: A Comprehensive Review, *IEEE/ACM Trans Comput Biol Bioinform.* **9**, 185-202.
48. Kimura, S., Sato, M. & Okada-Hatakeyama, M. (2015) An Effective Method for the Inference of Reduced S-system Models of Genetic Networks, *Inform Media Tech.* **10**, 166-174.
49. Kobayashi, S. (2009) The Frontiers of Real-coded Genetic Algorithms, *J Jpn Soc Artif Intell.* **24**, 147-162.
50. Runarsson, T. P. & Yao, X. (2005) Search biases in constrained evolutionary optimization, *IEEE Transactions on Systems, Man, and Cybernetics, Part C: Applications and Reviews.* **35**, 233-243.
51. Maeda, K., Boogerd, F. C. & Kurata, H. (2018) libRCGA: a C library for real-coded genetic algorithms for rapid parameter estimation of kinetic models, *IPSJ Transactions on Bioinformatics.* **11**, 31-40.
52. Hindmarsh, A. C., Brown, P. N., Grant, K., E., Lee, S. L., Serban, R., Shumaker, D. E. & Woodward, C. S. (2005) SUNDIALS: Suite of Nonlinear and Differential/Algebraic Equation Solvers, *ACM Trans Math Softw.* **31**, 363-96.
53. Martinelle, K. & Haggstrom, L. (1997) On the dissociation constant of ammonium: Effects of using an incorrect pK(a) in calculations of the ammonia concentration in animal cell cultures, *Biotechnology Techniques.* **11**, 549-551.
54. Lang, W., Block, T. M. & Zander, R. (1998) Solubility of NH3 and apparent pK of NH4+ in human plasma, isotonic salt solutions and water at 37 degrees C, *Clinica chimica acta; international journal of clinical chemistry.* **273**, 43-58.
55. Slonczewski, J. L., Rosen, B. P., Alger, J. R. & Macnab, R. M. (1981) pH homeostasis in Escherichia coli: measurement by 31P nuclear magnetic resonance of methylphosphonate and phosphate, *Proc Natl Acad Sci U S A.* **78**, 6271-5.

56. Slonczewski, J. L., Fujisawa, M., Dopson, M. & Krulwich, T. A. (2009) Cytoplasmic pH measurement and homeostasis in bacteria and archaea, *Adv Microb Physiol.* **55**, 1-79, 317.

57. Meek, T. D. & Villafranca, J. J. (1980) Kinetic mechanism of Escherichia coli glutamine synthetase, *Biochemistry.* **19**, 5513-9.

58. Kleiner, D. (1985) Energy expenditure for cyclic retention of NH3/NH4+ during N2 fixation by Klebsiella pneumoniae, *FEBS Lett.* **187**, 237-9.

59. Walter, A. & Gutknecht, J. (1986) Permeability of small nonelectrolytes through lipid bilayer membranes, *The Journal of membrane biology.* **90**, 207-17.

60. Mathai, J. C., Sprott, G. D. & Zeidel, M. L. (2001) Molecular mechanisms of water and solute transport across archaebacterial lipid membranes, *J Biol Chem.* **276**, 27266-71.

61. Khademi, S., O'Connell, J., 3rd, Remis, J., Robles-Colmenares, Y., Miercke, L. J. & Stroud, R. M. (2004) Mechanism of ammonia transport by Amt/MEP/Rh: structure of AmtB at 1.35 A, *Science.* **305**, 1587-94.

62. Folsom, J. P. & Carlson, R. P. (2015) Physiological, biomass elemental composition and proteomic analyses of Escherichia coli ammonium-limited chemostat growth, and comparison with iron- and glucose-limited chemostat growth, *Microbiology.* **161**, 1659-70.

63. Cayley, S., Lewis, B. A., Guttman, H. J. & Record, M. T. (1991) Characterization of the cytoplasm of Escherichia coli K-12 as a function of external osmolarity, *Journal of Molecular Biology.* **222**, 281-300.

64. Schmidt, A., Kochanowski, K., Vedelaar, S., Ahrne, E., Volkmer, B., Callipo, L., Knoops, K., Bauer, M., Aebersold, R. & Heinemann, M. (2016) The quantitative and condition-dependent Escherichia coli proteome, *Nat Biotechnol.* **34**, 104-10.

65. Bremer, H. & Dennis, P. P. (1996) Modulation of Chemical Composition and Other Parameters of the Cell by Growth Rate in *Escherichia coli and Salmonella: Cellular and Molecular Biology* (Neidhardt, F. C., ed) pp. 1553-69, ACM Press, Washington (D. C.).

66. Garcia-Contreras, R., Vos, P., Westerhoff, H. V. & Boogerd, F. C. (2012) Why in vivo may not equal in vitro - new effectors revealed by measurement of enzymatic activities under the same in vivo-like assay conditions, *FEBS J.* **279**, 4145-59.

67. Heeswijk, W. C. (1998) *The glutamine synthetase adenylylation cascade: A search for its control and regulation*, Vrije Universiteit, Amsterdam, the Netherlands.

68. van Heeswijk, W. C., Molenaar, D., Hoving, S. & Westerhoff, H. V. (2009) The pivotal regulator GlnB of Escherichia coli is engaged in subtle and context-dependent control, *FEBS J.* **276**, 3324-40.

69. Senior, P. J. (1975) Regulation of nitrogen metabolism in Escherichia coli and Klebsiella aerogenes: studies with the continuous-culture technique, *J Bacteriol.* **123**, 407-18.

70. Javelle, A., Lupo, D., Li, X. D., Merrick, M., Chami, M., Ripoche, P. & Winkler, F. K. (2007) Structural and mechanistic aspects of Amt/Rh proteins, *Journal of structural biology.* **158**, 472-81.

71. Zheng, L., Kostrewa, D., Berneche, S., Winkler, F. K. & Li, X. D. (2004) The mechanism of ammonia transport based on the crystal structure of AmtB of Escherichia coli, *Proc Natl Acad Sci U S A.* **101**, 17090-5.

72. Wacker, T., Garcia-Celma, J. J., Lewe, P. & Andrade, S. L. (2014) Direct observation of electrogenic NH4(+) transport in ammonium transport (Amt) proteins, *Proc Natl Acad Sci U S A.* **111**, 9995-10000.

73. Kleiner, D. (1985) Bacterial Ammonium Transport, *FEMS Microbiology Letters.* **32**, 87-100.

74. Blauwkamp, T. A. & Ninfa, A. J. (2003) Antagonism of PII signalling by the AmtB protein of Escherichia coli, *Mol Microbiol.* **48**, 1017-28.

75. Atkinson, M. R. & Ninfa, A. J. (1999) Characterization of the GlnK protein of Escherichia coli, *Mol Microbiol.* **32**, 301-13.

76. Davidi, D., Noor, E., Liebermeister, W., Bar-Even, A., Flamholz, A., Tummler, K., Barenholz, U., Goldenfeld, M., Shlomi, T. & Milo, R. (2016) Global characterization of in vivo enzyme catalytic rates and their correspondence to in vitro kcat measurements, *Proc Natl Acad Sci U S A.* **113**, 3401-6.

77. Bar-Even, A., Noor, E., Savir, Y., Liebermeister, W., Davidi, D., Tawfik, D. S. & Milo, R. (2011) The moderately efficient enzyme: evolutionary and physicochemical trends shaping enzyme parameters, *Biochemistry.* **50**, 4402-10.

78. Ma, H., Boogerd, F. C. & Goryanin, I. (2009) Modelling nitrogen assimilation of Escherichia coli at low ammonium concentration, *J Biotechnol.* **144**, 175-83.

79. Cueto-Rojas, H. F., Milne, N., van Helmond, W., Pieterse, M. M., van Maris, A. J. A., Daran, J. M. & Wahl, S. A. (2017) Membrane potential independent transport of NH3 in the absence of ammonium permeases in Saccharomyces cerevisiae, *BMC Syst Biol.* **11**, 49.

80. Segel, I. H. (1975) *Enzyme kinetics: Behavior and analysis of rapid equilibrium and steady-state enzyme systems*, John Wiley & Sons Inc.

81. Lodeiro, A. & Melgarejo, A. (2008) Robustness in Escherichia coli glutamate and glutamine synthesis studied by a kinetic model, *J Biol Phys.* **34**, 91-106.

82. Kidd, P. B. & Wingreen, N. S. (2010) Modeling the role of covalent enzyme modification in Escherichia coli nitrogen metabolism, *Physical biology.* **7**, 016006.

83. Masaki, K., Maeda, K. & Kurata, H. (2012) Biological Design Principles of Complex Feedback Modules in the E. coli Ammonia Assimilation System, *Artif Life.* **18**, 53-90.

84. Mutalik, V. K., Shah, P. & Venkatesh, K. V. (2003) Allosteric interactions and bifunctionality make the response of glutamine synthetase cascade system of Escherichia coli robust and ultrasensitive, *J Biol Chem.* **278**, 26327-32.

85. Mutalik, V. K. & Venkatesh, K. V. (2007) A theoretical steady state analysis indicates that induction of Escherichia coli glnALG operon can display all-or-none behavior, *Biosystems.* **90**, 1-19.

86. Hart, Y., Madar, D., Yuan, J., Bren, A., Mayo, A. E., Rabinowitz, J. D. & Alon, U. (2011) Robust control of nitrogen assimilation by a bifunctional enzyme in E. coli, *Mol Cell.* **41**, 117-27.

87. Ventura, A. C., Jiang, P., Van Wassenhove, L., Del Vecchio, D., Merajver, S. D. & Ninfa, A. J. (2010) Signaling properties of a covalent modification cycle are altered by a downstream target, *Proc Natl Acad Sci U S A.* **107**, 10032-7.

88. Wang, L., Lai, L., Ouyang, Q. & Tang, C. (2011) Flux balance analysis of ammonia assimilation network in E. coli predicts preferred regulation point, *PLoS ONE.* **6**, e16362.

89. Ma, H., Boogerd, F. C. & Goryanin, I. (2010) Corrigendum to "Modelling nitrogen assimilation of Escherichia coli at low ammonium concentration" [J. Biotechnol. 144 (2009) 175–183], *J Biotechnol.* **150**, 207-207.

90. Lee, W. P. & Hsiao, Y. T. (2012) Inferring gene regulatory networks using a hybrid GA-PSO approach with numerical constraints and network decomposition, *Inform Sciences.* **188**, 80-99.

91. Jaqaman, K. & Danuser, G. (2006) Linking data to models: data regression, *Nat Rev Mol Cell Biol.* **7**, 813-9.

92. Klipp, E., Liebermeister, W., Wierling, C., Kowald, A., Lehrach, H. & Herwig, R. (2009) *Systems Biology: A*

Textbook, Wiley-VCH, Germany.
